# Rational Design, Synthesis and Binding Affinity Studies of Anthraquinone Derivatives Conjugated to Gonadotropin-Releasing Hormone (GnRH) Analogues towards Selective Immunosuppression of Hormone-Dependent Cancer

**DOI:** 10.3390/ijms242015232

**Published:** 2023-10-16

**Authors:** Georgia Biniari, Christos Markatos, Agathi Nteli, Haralambos Tzoupis, Carmen Simal, Alexios Vlamis-Gardikas, Vlasios Karageorgos, Ioannis Pirmettis, Panagiota Petrou, Maria Venihaki, George Liapakis, Theodore Tselios

**Affiliations:** 1 Department of Chemistry, University of Patras, 26504 Rion, Greece; georgiabiniari96@gmail.com (G.B.); agathinteli@gmail.com (A.N.); haralambostz@gmail.com (H.T.); csimal@upatras.gr (C.S.); avlamis@upatras.gr (A.V.-G.); 2 Department of Pharmacology, School of Medicine, University of Crete, 71003 Heraklion, Greece; xristosmarkatos@gmail.com (C.M.); bkarageorgos@hotmail.com (V.K.); 3 Institute of Nuclear & Radiological Sciences & Technology, Energy & Safety, National Centre for Scientific Research “Demokritos”, 15341 Athens, Greece; ipirme@rrp.demokritos.gr (I.P.); pnpetrou@rrp.demokritos.gr (P.P.); 4 Department of Clinical Chemistry, School of Medicine, University of Crete, 71003 Heraklion, Greece; venycham@uoc.gr

**Keywords:** gonadotropin-releasing hormone (GnRH), mitoxantrone, hormone-dependent cancer, thioredoxin system, targeted cancer therapy

## Abstract

Gonadotropin-releasing hormone (GnRH) is pivotal in regulating human reproduction and fertility through its specific receptors. Among these, gonadotropin-releasing hormone receptor type I (GnRHR I), which is a member of the G-protein-coupled receptor family, is expressed on the surface of both healthy and malignant cells. Its presence in cancer cells has positioned this receptor as a primary target for the development of novel anti-cancer agents. Moreover, the extensive regulatory functions of GnRH have underscored decapeptide as a prominent vehicle for targeted drug delivery, which is accomplished through the design of appropriate conjugates. On this basis, a rationally designed series of anthraquinone/mitoxantrone–GnRH conjugates (**con1**–**con8**) has been synthesized herein. Their in vitro binding affinities range from 0.06 to 3.42 nM, with six of them (**con2**–**con7**) demonstrating higher affinities for GnRH than the established drug leuprolide (0.64 nM). Among the mitoxantrone based GnRH conjugates, **con3** and **con7** show the highest affinities at 0.07 and 0.06 nM, respectively, while the disulfide bond present in the conjugates is found to be readily reduced by the thioredoxin (Trx) system. These findings are promising for further pharmacological evaluation of the synthesized conjugates with the prospect of performing future clinical studies.

## 1. Introduction

Gonadotropin-releasing hormone (GnRH) or luteinizing hormone-releasing hormone (LHRH), has a fundamental role in the reproductive function in mammalian cells, constituting the initial step of hypothalamic–pituitary–gonadal axis regulation. GnRH was isolated and identified by A.V. Schally and R.C.L. Guillemin in 1971 [1] as a decapeptide (pGlu^1^-His^2^-Trp^3^-Ser^4^-Tyr^5^-Gly^6^-Leu^7^-Arg^8^-Pro^9^-Gly^10^NH_2_). It is responsible for the regulation and release of gonadotropins, namely follicle-stimulating hormone (FSH), and luteinizing hormone (LH) from the anterior pituitary [1,2,3]. In particular, GnRH binds to and activates GnRH receptors (GnRHRs) on the surface of the pituitary gonadotrope cells, thus initiating the synthesis and secretion of LH and FSH. In turn, LH and FSH act on the gonads and regulate steroidogenesis and gametogenesis [2,4]. LH induces testosterone production and supports the proliferation of the Leydig cells in males, also inducing the synthesis of androgens and hormonal precursors for estradiol production in the theca cells in the ovaries. FSH, on the other hand, acts upon the Sertoli cells of the testes, supporting the maturation of gametes and enhancing spermatogenesis, while it also stimulates the maturation of the ovarian follicles affecting granulosa cells [5,6,7,8,9].

The pulsatile secretion of GnRH (with frequencies from ~30 min to 120 min) from the hypothalamus is critical for the regulation of the reproductive cycle [10,11,12,13]. In addition to the hypothalamus, GnRHR I expression has been reported from cancer cells located in breast tissues, ovaries, endometrium, and prostate. Studies have shown that GnRHR I is expressed also in extrapituitary tissues unrelated to the reproductive system, including the lungs and the pancreas [14,15,16,17,18]. The GnRH-GnRHR I complex constitutes an important target for infertility treatments and cancer therapy [19,20,21]. Although there are crystallographic data for GnRHR I [22], many research groups focus on further structural conformation of GnRHR I in order to gain further insight into GnRH-GnRHR I dynamics and provide valuable information that can be applied to targeted treatments [23,24,25].

Several GnRH analogues, used as therapeutic agents, have been developed as agonists and antagonists [26]. Since the GnRH half-life is very short (2–4 min) [27], GnRH analogues with altered sequences have been developed. Solid-phase peptide synthesis has contributed to the rapid production of such analogues [28], introducing, namely, modifications at the *C*-terminal of GnRH. Analogues with the substitution of glycine amide (Gly-NH_2_) and/or Gly in position 6 with an ethyl amide and a D-amino acid, respectively, have demonstrated greater potency and enhanced properties against proteolysis [29,30]. Leuprolide [31,32,33], buserelin [34,35], triptorelin [36], nafarelin [37,38,39] and goserelin [40] are some of the approved GnRH agonists for the treatment of prostate and breast cancer. GnRH agonists stimulate LH and FSH expression continuously, leading to the “flare” effect and subsequently to the desensitization and downregulation of the GnRHR. After the administration of GnRH agonists, a transient deterioration of the existing symptoms is observed, which can cause discomfort in patients [41,42,43,44,45].

An encouraging concept, proposed in this paper, is the design and development of delivery systems based on GnRH analogues, conjugated with anti-cancer drugs, and targeting the GnRHR I expressed on cancer cells [26,46]. A strategy based on the selective recognition of cancer cells using a carrier molecule linked to anti-neoplastic agents is a promising approach since it limits the side-effects of chemotherapy [47]. According to the literature, conjugates with similar structures to GnRH demonstrate significantly higher affinity for GnRHR compared to the free drug. In particular, coupling an anti-cancer agent to a peptide analogue enhanced its antiproliferative efficacy compared to the administration of a standalone anti-cancer agent [48,49]. Furthermore, the toxicity levels associated with GnRH conjugates were lower in comparison to their anti-neoplastic counterparts owing to the enhanced stability of the conjugates in serum [48,50,51]. Along these lines, the GnRH agonist DLys^6^-GnRH, conjugated via a covalent bond with doxorubicin (Zoptarelin doxorubicin or AEZS-108 or AN-152), was proven to exhibit high binding affinity when tested in human breast, ovarian and endometrial cancer cells [52,53,54,55]. When tested in GnRHR(+) experimental cancers in mice, it demonstrated lower toxicity levels and improved action on growth inhibition compared to the use of the cytotoxic doxorubicin alone [56,57]. Although AEZS-108 did not meet the relevant endpoints in a phase III clinical trial, it was used to establish a new approach based on GnRH-hybrid molecules with enhanced physicochemical properties [58,59].

In this study, we present a novel approach for the treatment of hormone-related cancer based on chemical modifications of the AEZS-108 conjugate. Such modifications aim to improve drug potency via increased binding efficiency. Our strategy focuses on three key points: (i) the implementation of a GnRH peptide analogue based on the structure of leuprolide and a D-amino acid substitution at position 6; (ii) the use of a mitoxantrone analogue as a cytotoxic agent; and (iii) the covalent connection of the two aforementioned parts through a disulfide bridge, which lacks the disadvantage of the hydrolysis of previous GnRH conjugates via serum carboxylesterase enzymes [60].

## 2. Results and Discussion

### 2.1. Rational Design of Mitoxantrone–GnRH Analogues

Our aim was to design and synthesize a series of GnRH analogues, conjugated to a cytotoxic agent via a disulfide to specifically target the overexpressed GnRH receptors on the surface of cancer cells [16,61]. The implemented cytotoxic agents comprised the anthraquinone derivative mitoxantrone (Novantrone^®^) [62,63], which is a well-established anti-neoplastic agent also used for the treatment of multiple sclerosis (MS) [64,65], non-Hodgkin’s lymphoma [66,67], and breast and prostate cancer [68,69]. Mitoxantrone was chosen due to its improved pharmacological profile relative to doxorubicin and its fewer side-effects, particularly in patients with a high risk of cardiovascular complications [70,71,72,73]. Instead of using an ester bond, as in AEZS-108, we opted for a disulfide linkage in anticipation of its potential reduction via the thioredoxin (Trx) system of cellular surfaces [74]. Such a reduction would subsequently trigger the release of the cytotoxic agent selectively near the cancer cells [75]. Previously conducted studies have already shown the successful reduction of the disulfide bond via the Trx system, leading to the release of the anthraquinone moiety and its entrance into the cell [76]. The reduction of the disulfide bond could be also attained via the cytoplasmic thioredoxin system upon the binding of the GnRH conjugate to GnRHR I and the subsequent internalization of the ligand–receptor complex.

**Scheme 1:**
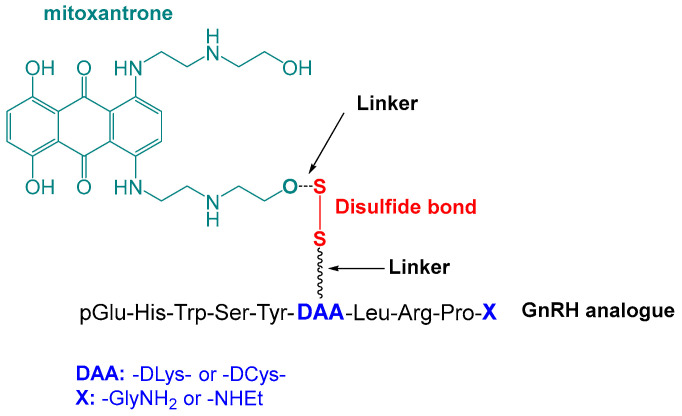
Representative structure of study mitoxantrone conjugates.

Given the high expression of GnRHR in hormone-related cancers and considering the key points outlined above, our primary objective was to develop altered peptide conjugates with selective binding to GnRHR and with the capacity to facilitate the subsequent release of the incorporated cytotoxic compound (mitoxantrone) (Scheme 1). Throughout the synthetic process, the ethyl amide counterpart, found in leuprolide (see Section S1.5, Figures S9 and S10), was retained in the structure as it increases binding affinity for GnRHR. Furthermore, DLeu^6^ was replaced by DLys^6^/DCys^6^ residues, which possess similarly sized charged side chains. In the case of the DCys^6^ conjugate, the disulfide was directly bound to mitoxantrone by modifying a terminal hydroxyl group in the drug using a thiol group. The amide moiety in the DLys^6^ conjugate allowed for the incorporation of a linker via the formation of a peptide bond. This linker was designed to feature a thiol group for the subsequent formation of the disulfide bridge with the anthraquinone analogue. To this end, an aminohexanoic acid coupled with SPDP [succinimidyl 3-(2-pyridyldithio) propionate] as a thiol precursor donor acted as such a linker, providing flexibility to the resulting molecule.

Quinizarin conjugates (**con1**, **con2**, **con4**–**6** and **con8**) were initially synthesized to establish reactions procedures and assess their binding affinities to GnRHR. After the synthesis and evaluation of these conjugates, mitoxantrone was used for the synthesis of the potent conjugates **con3** and **con7**. Both mitoxantrone and the quinizarin analogues share a di-substituted amino alkyl-amino moiety and an anthraquinone ring. These structural features have been widely investigated for their anti-neoplastic properties, which are mainly attributed to the interference of the anthraquinone ring amongst DNA nucleobase pairs and the interaction between the nitrogen groups in the alkyl chains and the negatively charged phosphorous groups of DNA [77,78,79]. Additionally, a thiol group was incorporated into the alkyl chain of the synthesized analogues in order to facilitate the formation of a disulfide bridge between the anthraquinone and the GnRH analogues.

### 2.2. Synthesis of Anthraquinone and Mitoxantrone-GnRH Analogues

#### 2.2.1. Synthesis of Anthraquinone—GnRH Conjugates **con4**; **con5**; **con6**

**Scheme 2:**
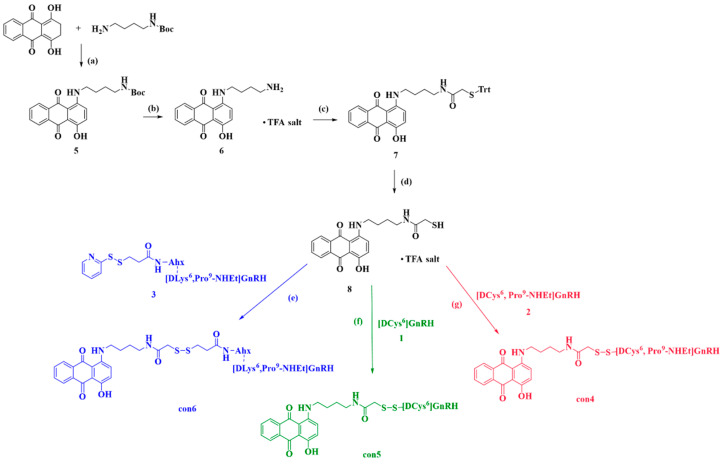
Synthetic pathway for the synthesis of anthraquinone–GnRH conjugates **con4**, **con5** and **con6**. Reagents and conditions: (**a**) (i) ethanol, Ar, from reflux for 3 h to 50 °C for 16 h; (ii) [O], room temperature (RT), 3 h (oxidation in flowing air) (Figures S11–S13); (**b**) TFA, CH_2_Cl_2_, RT, 2 h (Figures S14–S16); (**c**) TrtSCH_2_CO_2_H, DCC, DMAP, CH_2_Cl_2_, RT, 5 h (Figures S17–S19); (**d**) TFA, TES, CH_2_Cl_2_, RT, 3 h (Figures S20 and S21); (**e**) **3**, DIPEA, DMSO, RT, 4.5 h (Figures S26 and S27); (**f**) **1**, DIPEA, DMSO, RT, 52 h (Figures S24 and S25); (**g**) **2**, DIPEA, DMSO, RT, 24 h (Figures S22 and S23). The synthetic procedures for analogues **1**–**8** are described thoroughly in Supplementary Materials (see Section S2.1).

Conjugates **con4**, **con5** and **con6** were prepared according to the synthetic protocol outlined in Scheme 2. **Con4** and **con5** were synthesized via the oxidation of thiols groups; however, **con6** was prepared via a thiol–disulfide exchange reaction. The same starting material, analogue **8** (see Section S2.1.4), reacted with synthesized [DLys(Ahx-PDP)^6^, Pro^9^-NHEt]GnRH (analogue **3**; see Section S1.3, Figures S5 and S6), [DCys^6^]GnRH peptide (analogue **1**; see Section S1.1, Figures S1 and S2) and [DCys^6^, Pro^9^-NHEt]GnRH peptide (analogue **2**; see Section S1.2, Figures S3 and S4) for the synthesis of **con6**, **con5** and **con4**, respectively.

#### 2.2.2. Synthesis of Anthraquinone—GnRH Conjugates **con1**; **con2**; **con8**

**Scheme 3:**
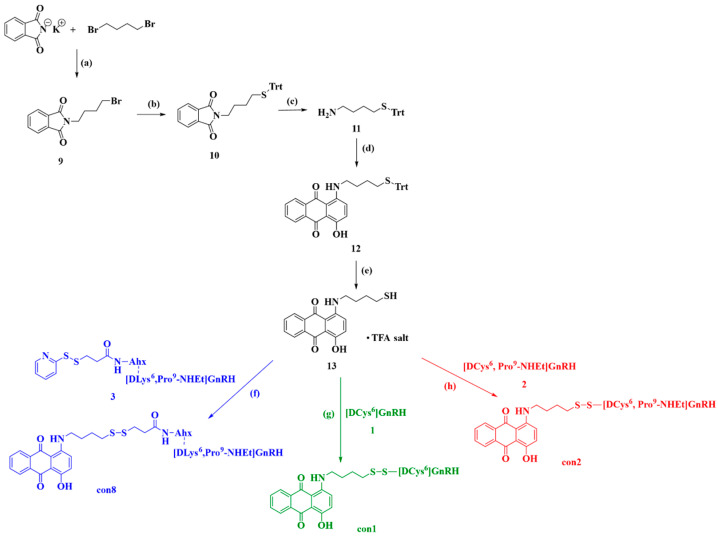
Synthetic pathway for the synthesis of anthraquinone–GnRH conjugates **con1**, **con2** and **con8**. Reagents and conditions: (**a**) DMF anhydrous, Ar, from 0 °C to RT for 16 h (Figures S28–S30); (**b**) (Ph)_3_CSH, NaH, DMF anhydrous, Ar, from 0 °C to RT for 16 h, (Figures S31 and S32); (**c**) N_2_H_4_, *n*-butanol/ethanol, reflux, 24 h (Figures S33–S35); (**d**) (i) leucoquinizarin, ethanol, from reflux for 3 h to 50 °C for 18 h; (ii) [O], RT, 3 h (oxidation in flowing air) (Figures S36–S38); (**e**) TFA, TES, CH_2_Cl_2_, RT, 5 h (Figures S39–S41); (**f**) **3**, DIPEA, DMSO, RT, 4 h; (**g**) **1**, DIPEA, DMSO, RT, 48 h; (**h**) **2**, DIPEA, DMSO, RT, 44 h. The synthetic procedures for analogues **9**–**13** are described thoroughly in Supplementary Materials (see Section S2.2).

Conjugates **con1**, **con2** and **con3** were prepared according to the synthetic protocol outlined in Scheme 3. **Con1** and **con2** were synthesized analogously to **con4** and **con5** and **con8** to **con6**. Again, the same starting substrate was used for all reactions. Thus, analogue **13** (see Section S2.2.5) reacted with analogue **3** (see Section S1.3, Figures S5 and S6), analogue **1** (see Section S1.1, Figures S1 and S2) and analogue **2** (see Section S1.2, Figures S3 and S4), to give the desired final compounds **con1**, **con2** and **con8**, respectively.

#### 2.2.3. Synthesis of Anthraquinone—GnRH Conjugates **con7**; **con3**

**Scheme 4:**
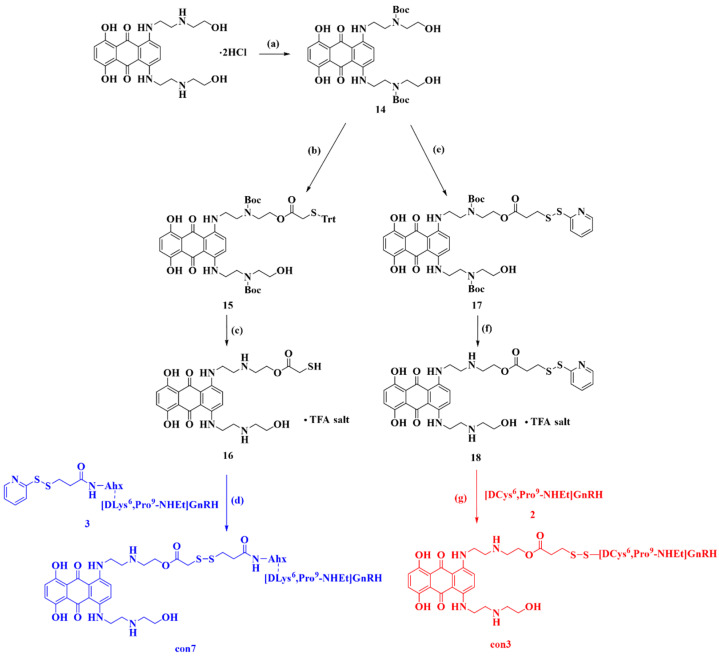
Synthetic pathway for the synthesis of mitoxantrone–GnRH conjugates **con7** and **con3**. Reagents and conditions: (**a**) Boc_2_O, anhydrous MeOH/THF, TEA, from 0 °C for 1 h to RT for 2 h (Figures S48–S50); (**b**) TrtSCH_2_CO_2_H, DMAP, HOBt, DIC, CH_2_Cl_2_, 0 °C, 24 h (Figures S51–S53); (**c**) TFA, TES, CH_2_Cl_2_, RT, 1.5 h (Figures S54–S56); (**d**) **3**, MeOH, RT, 6 h (Figures S57 and S58); (**e**) 3-(2-pyridyldithio)propanoic acid, DMAP, HOBt, DIC, CH_2_Cl_2_, 0 °C, 24 h (Figures S59 and S60); (**f**) TFA, TES, CH_2_Cl_2_, 1.5 h (Figures S61–S63); (**g**) **2**, MeOH, RT, 24 h (Figures S64 and S65). The synthetic procedures for analogues **14**–**18** are described thoroughly in Supplementary Materials (see Section S2.3).

The synthetic protocols for conjugates **con7** and **con3** are described in Scheme 4. Analogue **16** (see Section S2.3.1) reacted with synthesized analogue **3** (see Section S1.3, Figures S5 and S6), while analogue **18** (see Section S2.3.2) reacted with analogue **2** (see Section S1.2, Figures S3 and S4), to give the desired final compounds **con7** and **con3**, respectively.

### 2.3. Conformational Studies of ***con7*** and ***con3***

MD simulations provide the tools needed to analyze the conformations of analogues in aqueous solution. The analysis of RMSD values for the backbone atoms of (DLys^6^, Pro^9^-NHEt)GnRH peptide (analogue **4**, see Section S1.4, Figures S7 and S8), included in **con7**, shows that it underwent small changes from the initial peptide conformation over the MD simulation time (Figure S66A). Similarly, RMSD values for the backbone atoms in (DCys^6^, Pro^9^-NHEt)GnRH peptide (analogue **2**), included in **con3**, do not vary greatly in the first 100 ns and show a greater variability over the last 50 ns of simulation (Figure S66B). At the respective conjugates, **con7** presents small conformational changes at the start of the simulation; conversely, at 60 ns, the conjugate presents a change in its conformation that is present until the end of the simulation (Figure S66C). On the other hand, the RMSD values of **con3** are smaller than those of the respective peptide, but a steep leap is observed around 35 ns and a differentiation also appears between 55 and 70 ns (Figure S66D). The small conformational changes in analogue **4** and **con7** are mirrored in the atomic fluctuations in which the mean atomic fluctuations for each residue are similar for these two analogues (Figure S67A). The only difference is observed in the values for DLys^6^ residue in the **con7** (Figure S67A). These changes can be attributed to the modified side chain of DLys^6^ and the attachment of the mitoxantrone analogue (**con7**). In the same manner, the atomic fluctuations of analogue **2** mirror the small conformational changes of residues 1–7 and 9, while residues 8 and 10 have higher fluctuation values (Figure S67B). These changes in the residues could be attributed to the small carbon side chain of DCys^6^, which possibly provides more flexibility to the peptide compared to the analogue **4**. The atomic fluctuations of **con3** are higher than those of analogue **2**. An exception is the case of Ser^4^, which has the smallest value due to hydrogen bond formation between Ser^4^ and DCys^6^ (Figure S67B).

**Figure 1:**
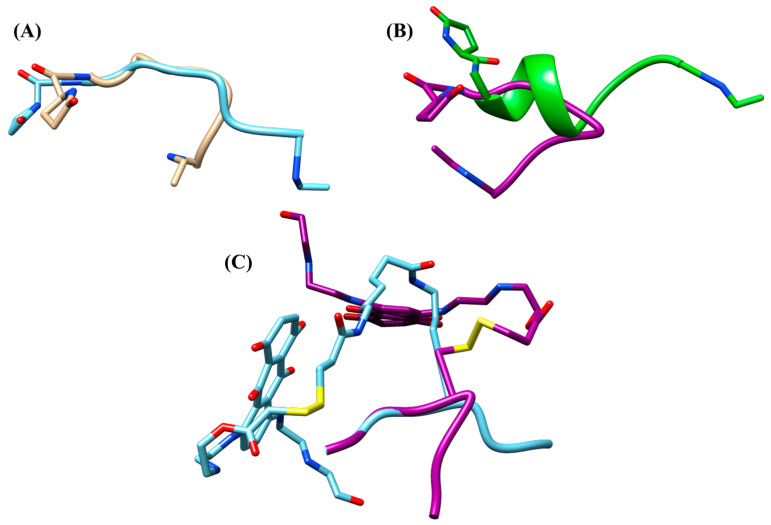
Comparison of the dominant conformations for **con7**, **con3** and their respective peptides. (**A**) Comparison of **con7** (cyan) and analogue **4** (beige); (**B**) Comparison of **con3** (purple) and analogue **2** (green); and (**C**) Comparison of **con7** (cyan) and **con3** (purple). Atoms in yellow, red and dark blue colours are sulfur (S), oxygen (O) and nitrogen (N), respectively.

The clustering analysis revealed the presence of two clusters for analogue **4** and analogue **2**, as well as three clusters for conjugates **con7** and **con3**. The dominant cluster 1 of analogue **4** was present for 71% of the time (Figure 1A). Similarly, the dominant cluster 1 of analogue **2** was present for 58% of the time (Figure 1B). On the other hand, the dominant cluster 1 of **con7** was present for 45% of the time (Figure 1A) and the dominant cluster 1 of **con3** was present for 50% of the time (Figure 1C). The dominant clusters for both peptide conjugates revealed the differentiations that occur from changes in position 6. The substitution with DLys allowed the peptide to adopt a U-conformation (Figure 1A beige), which is considered favorable for binding to GnRHR, while substitution with DCys led to a helical conformation (Figure 1B green). These conformations were changed in the conjugates. In the case of **con7** (Figure 1A cyan), the U-formation was now more extended. Conversely, **con3** (Figure 1B purple), the helical conformation, changed into a closed U-structure. In both conjugates (Figure 1C), the most important characteristic was the retention of a bent conformation in position 6, while the disulfide bridge in both cases was exposed to the solvent. The exposed positioning of the disulfide bond in both cases can potentially allow for the faster reduction of the bond via the thioredoxin system and lead to the rapid release of mitoxantrone analogue.

Further analysis of the conformational characteristics revealed the secondary structural elements of the synthesized analogues. The analogue **4** presented beta-turn features (Figure S68A) between Trp^3^ and DLys^6^ due to the presence of a hydrogen bond between Trp^3^ (i residue) and DLys^6^ (i + 3 residue), while the distance between the residues was <7 Å (5.33 Å). Additionally, **con7** also showed beta-turn (Figure S68C) features between Trp^3^ and Tyr^5^. The analogue **2** also presented beta-turn features (Figure S68B) between Trp^3^ and Leu^7^. However, as mentioned above, we found that there were α-helical characteristics shared between residues Trp^3^ and DCys^6^ (Figure 1B (green) and Figure S68B), with a hydrogen bond between Trp^3^ (i residue) and DCys^6^ (i+4 residue). On the other hand, the addition of mitoxantrone to create **con3** altered the conformation from helical to bent (Figure S68D).

The most important aspect observed during the conformational analysis was the bent conformation adopted by residues in position 6 (Figure 1C). This conformation allowed the side chains of residues in position 6 to be exposed to the solvent. Specifically, **con7** had a peptide skeleton that was more extended from the respective peptide (analogue **4**), meaning that the residues were more available to interact with the solvent (H_2_O) or other residues (e.g., GnRH receptor, proteins). On the contrary, **con3** appeared to adopt a tighter U-shape (Figure 1B), but still the residues were exposed to the solvent and thus retained the ability to readily interact with residues on GnRHR. The secondary structural characteristics of both conjugates revealed that the positioning of important residues (His^2^, Trp^3^ and Arg^8^) involved in GnRHR binding was exposed to the solvent and thus allowed the conjugates to bind strongly to the receptor (Figure S69 and Table S1) [80]. Moreover, as mentioned above, the conformation of the conjugates exposed the disulfide bond to the action of the thioredoxin system.

### 2.4. Binding Affinity Studies of Synthesized Conjugates

**Figure 2:**
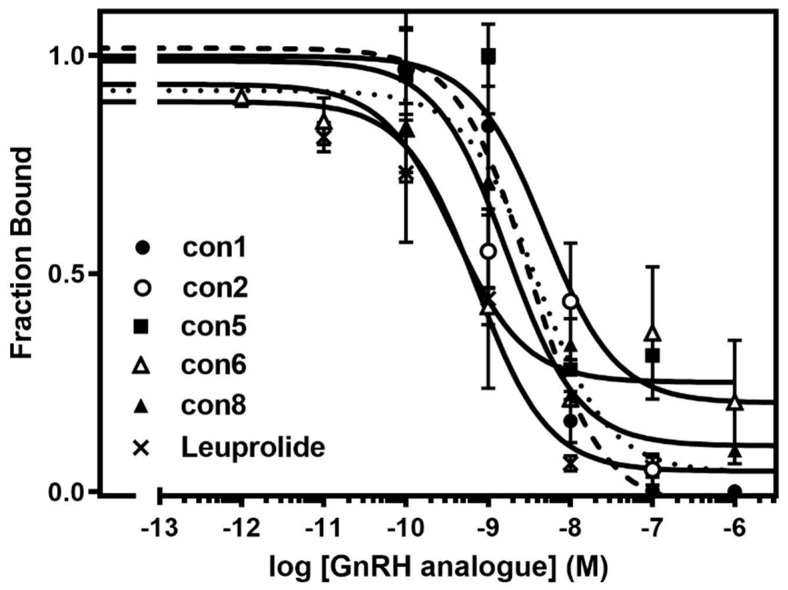
Competition binding isotherms of GnRH analogues to human GnRHR. We compared ^125^I-Tyr^6^, His^5^-GnRH-specific binding by increasing concentrations of GnRH analogues, **con1**, **con 2**, **con5**, **con6**, **con8**, or leuprolide, as described in Section 3.4, present on membranes from HEK 293 cells stably expressing human GnRHR I. The means and S.E. are shown from a representative experiment performed 3–6 times with similar results. The data were fitted to a one-site competition model via nonlinear regression and the IC_50_ values were determined as described in Section 3.4. **Con4** is not included due to solubility issues and the fact that binding affinity could not be accurately estimated.

**Figure 3:**
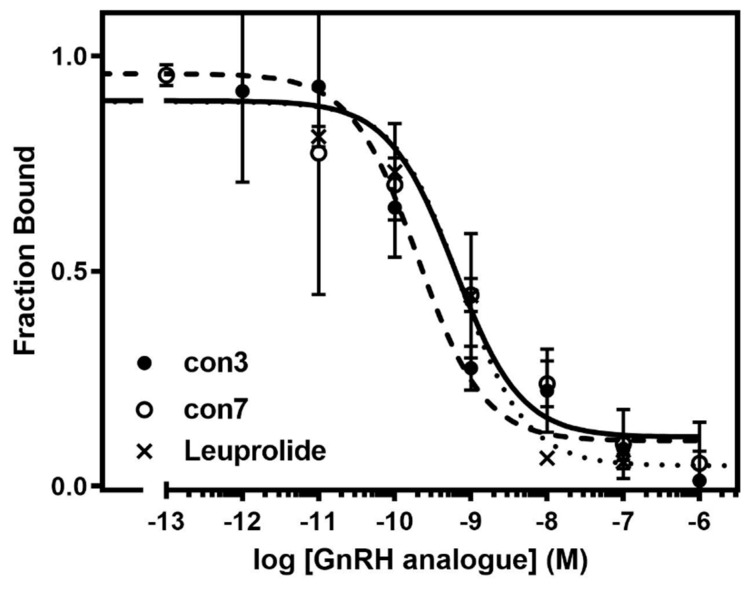
Competition binding isotherms of **con3** and **con7** to human GnRHR. Competition of ^125^I-Tyr^6^, His^5^-GnRH-specific binding by increasing concentrations of GnRH analogues, **con3**, **con7** and leuprolide was performed, as described in Section 3.4, on membranes from HEK 293 cells stably expressing human GnRHR I. The means and S.E. are shown from a representative experiment performed 3–6 times with similar results. The data were fitted to a one-site competition model via nonlinear regression and the IC_50_ values were determined as described in Section 3.4.

The apparent binding affinities (IC_50_) of the novel GnRH analogues, conjugated with anthraquinone or mitoxantrone (**con1**–**con8**) for human GnRHR I, were determined using competitive radioligand binding assays. In these experiments, membranes from HEK 293 cells, stably expressing GnRHR I, were incubated with ^125^I-Tyr^6^, His^5^-GnRH in the presence and absence of increasing concentrations of GnRH analogues or leuprolide (positive control). All GnRH analogues reduced the specific binding of ^125^I-DTyr^6^-His^5^-GnRH in a dose-dependent manner, with affinities (0.06–3.42 nM) that were higher than or similar to those of leuprolide (0.64 nM) (Figure 2 and Figure 3). The compounds with the highest binding affinities were **con3** (0.07 nM), **con6** (0.07 nM) and **con7** (0.06 nM) (Figure 2 and Figure 3). In marked contrast to GnRH conjugates, mitoxantrone did not bind to GnRHR (see Section S5, Figure S70). Analogues **con3** and **con7** were further evaluated based on the presence of the anti-cancer drug, mitoxantrone, in their structure. Moreover, analogues **4** and **2** conjugated with mitoxantrone gave **con7** and **con3**, respectively, whose binding affinities were similar to those of their parental compounds (see Section S5, Figure S71).

### 2.5. Disulfides Reduction of ***con7*** and Insulin by EcoTrx1

**Figure 4:**
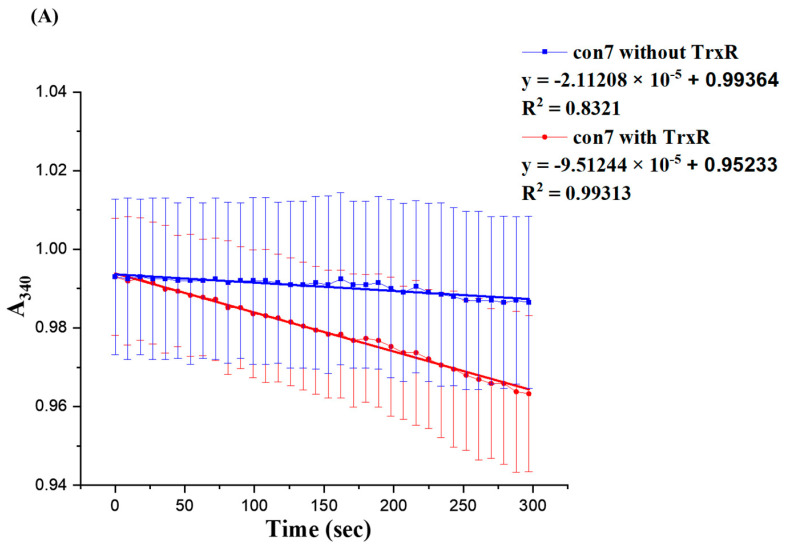

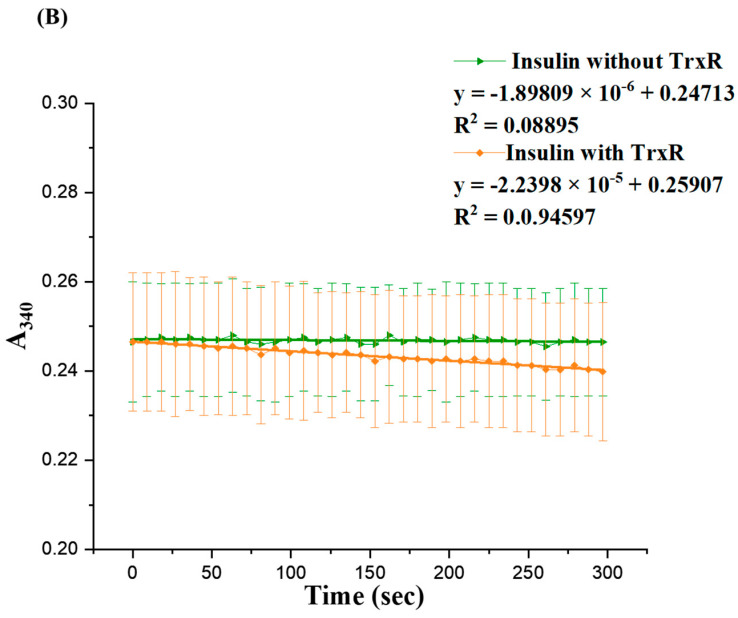
Reduction of (**A**) **con7** and (**B**) insulin by EcoTrx1. The assay mixture contains 200 mM potassium phosphate pH 7.4, 1 mM EDTA, 0.1 mg/mL BSA, 200 μM NADPH, 2 μM *Escherichia coli* Trx1, 10 nM *E. coli* TrxR, (**A**) 0.2 mM **con7** or (**B**) 0.067 mM (0.2 mM disulfides) insulin. The reaction was carried out at 25 °C by monitoring the decrease of NADPH absorbance at 340 nm for (**A**) **con7** in the absence (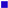) or presence of TrxR (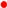) and (**B**) insulin in the absence (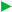) or presence of TrxR (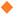).

The thioredoxin system can successfully reduce the disulfide bond of similar conjugates and release the anthraquinone moiety, as has been described in our previous studies [76]. For that reason, **con7** (one of the most promising candidates) was evaluated for the release of mitoxantrone analogue via the thioredoxin system. The reduction of **con7** was compared to the reduction of insulin containing three disulfide bonds. The reduction of **con7** and insulin from the thioredoxin system was successful, as assessed by the decrease of NADPH absorbance over time in samples containing TrxR (see Section S4). The absorbance values of **con7** were higher than those of insulin because of the presence of the anthraquinone aromatic system in the conjugate. Moreover, the reduction of **con7** was more rapid than that of insulin (Figure 4). This was most likely due to the exposure of **con7′s** disulfide bond to the solution and ability to react with the thioredoxin system, in contrast to the less exposed disulfides of insulin. In the samples that did not contain TrxR, the absorbance values saw a very slight decrease due to the oxidation of NADPH from atmospheric air (Figure 4).

## 3. Materials and Methods

All commercially available solvents and reagents were used without further purification and purchased from Merck (Darmstadt, Germany), Sigma Aldrich (St. Louis, MO, USA), Fluka (Buchs, Switzerland), Acros (Geel, Belgium), Fischer Chemical (Zurich, Switzerland), ChemBiotin (Voula, Greece), and Thermo Scientific (Waltham, MA, USA). Fmoc-protected amino acids, 2-chlorotrityl chloride (CLTR-Cl) resin (1% DVB, 200–400 mesh) and piperidine were purchased from Chemical and Biopharmaceutical Laboratories of Patras (Patras, Greece). Ethyl indole AM [3-([Ethyl-Fmoc-amino]-methyl)-1-indol-1-yl]-acetyl AM resin was purchased from Novabiochem and mitoxantrone dihydrochloride (CAS: 70476-82-3) was purchased from Fluorochem. Na^125^I was purchased from Perkin Elmer (Waltham, MA, USA); we acquired Triptorelin from Bachem (Bubendorf, Switzerland). HPLC-grade water was prepared using a Millipore Simplicity system (Burlington, MA, USA).

The purity of the synthesized analogues was determined via analytical reverse-phase high-performance liquid chromatography (RP-HPLC) (1260 Infinity, Quaternary Pump VL Agilent, Waldbronn, Germany) performed using an Agilent ZORBAX Eclipse Plus C18 column (3.5 μm, 100 × 4.6 mm, at 214 and 254 nm). The purification of products was achieved via flash column chromatography (silica gel, 200–400 mesh) or was carried out on a semi-preparative RP-HPLC system (Waters 600 solvent delivery system, combined with a Waters 996 photodiode array detector) using a Nucleosil RP-C18 column (7 μm, 250 × 10 mm, Merck, Darmstadt, Germany) and on a preparative RP-HPLC system (Waters Delta Prep, Empower Pro system, combined with a Waters 2996 photodiode array detector) using a Delta Pak RP-C18 column (15 μm, 300 × 19 mm, flow rate: 12 mL/min). Mobile phases were 0.08% TFA in H_2_O and 0.08% TFA in ACN. The radiolabeled peptide was purified via high-performance liquid chromatography (HPLC) with gradient elution (Waters 600 chromatography system, USA, coupled to a UV detector and NaI(Tl) detector in-line) using an analytical Macherey−Nagel Nucleosil RP C-18 column (250 × 4.6 mm, 5 μm, flow rate: 1 mL/min). Mobile phases were 0.05% TFA in H_2_O and 0.05% TFA in ACN.

Electrospray spray ionization mass spectrometry (ESI-MS) experiments were performed with a Waters Micromass ZQ Electrospray Platform coupled to a MassLynx2.3 data system and with a Bruker amaZon SL Ion Trap MS coupled to a TrapControl data system. Nuclear magnetic resonance spectra were obtained on 600 MHz spectrometer Bruker Avance 400 DPX, using tetramethylsilane (TMS) as reference standard.

### 3.1. Synthesis of Anthraquinone and Mitoxantrone-GnRH Analogues

#### 3.1.1. Synthesis of Anthraquinone—GnRH Conjugate **con4**

Analogue **2** (12.5 mg, 0.010 mmol, 1.0 eq) and analogue **8** (5.4 mg, 0.014 mmol, 1.4 eq) were dissolved in DMSO (0.4 mL) and DIPEA (0.015 mL). The reaction mixture was stirred in RT for 24 h and monitored via analytical RP-HPLC. Purification via semi-preparative HPLC (gradient separation 20 to 80% ACN in 40 min, flow rate 3 mL/min) provided conjugate **con4** as a purple solid (2.3 mg, 0.0015 mmol, 14.54%). M_theoritical_: 1581.83; (Μ_theoritical_ + H)^+^: 1582.83; found 1581.86; (Μ_theoritical_ + 2H^+^)/2: 791.92; found 791.77; (Μ_theoritical_ + 3H^+^)/3: 528.28; found 528.55; (Figures S22 and S23).

#### 3.1.2. Synthesis of Anthraquinone—GnRH Conjugate **con5**

Analogue **1** (19.8 mg, 0.016 mmol, 1.0 eq) and analogue **8** (8.6 mg, 0.022 mmol, 1.4 eq) were dissolved in DMSO (0.4 mL) and DIPEA (0.015 mL). The reaction mixture was stirred in RT for 52 h and monitored via analytical RP-HPLC. Purification performed using semi-preparative HPLC (gradient separation 20 to 80% ACN in 40 min, flow rate 3 mL/min) provided conjugate **con5** as a purple solid (2.7 mg, 0.0017 mmol, 10.48%). M_theoritical_: 1610.83; (Μ_theoritical_ + H)^+^: 1611.83; found 1611.66; (Μ_theoritical_ + 2H^+^)/2: 806.42; found 806.31; (Μ_theoritical_ + 3H^+^)/3: 537.94; found 538.05; (Figures S24 and S25).

#### 3.1.3. Synthesis of Anthraquinone—GnRH Conjugate **con6**

Analogue **3** (15.7 mg, 0.010 mmol, 1.0 eq) and analogue **8** (5.4 mg, 0.014 mmol, 1.4 eq) were dissolved in DMSO (0.4 mL) and DIPEA (0.015 mL). The reaction mixture was stirred in RT for 4.5 h and monitored via analytical RP-HPLC. Purification using semi-preparative HPLC (gradient separation 20 to 80% ACN in 40 min, flow rate 3 mL/min) provided conjugate **con6** as a purple solid (6.3 mg, 0.0035 mmol, 34.84%). M_theoritical_: 1808.15; (Μ_theoritical_ + H)^+^: 1809.15; found 1808.66; (Μ_theoritical_ + 2H^+^)/2: 905.08; found 904.99; (Μ_theoritical_ + 3H^+^)/3: 603.72; found 603.69; (Figures S26 and S27).

#### 3.1.4. Synthesis of Anthraquinone—GnRH Conjugate **con1**

Analogue **1** (16.6 mg, 0.014 mmol, 1.0 eq) and analogue **13** (6.5 mg, 0.020 mmol, 1.4 eq) were dissolved in DMSO (0.4 mL) and DIPEA (0.015 mL). The reaction mixture was stirred at RT for 48 h and monitored via analytical RP-HPLC. Purification using semi-preparative HPLC (gradient separation 20 to 80% ACN in 40 min, flow rate 3 mL/min) provided conjugate **con1** as a purple solid (3.7 mg, 0.0024 mmol, 17.02%). M_theoritical_: 1553.64; (Μ_theoritical_ + H)^+^: 1554.64; found 1554.08; (Μ_theoritical_ + 2H^+^)/2: 777.32; found 777.81; (Figures S42 and S43).

#### 3.1.5. Synthesis of Anthraquinone—GnRH Conjugate **con2**

Analogue **2** (9.5 mg, 0.008 mmol, 1.0 eq) and analogue **13** (3.6 mg, 0.011 mmol, 1.4 eq) were dissolved in DMSO (0.4 mL) and DIPEA (0.015 mL). The reaction mixture was stirred at RT for 44 h and monitored via analytical RP-HPLC. Purification using semi-preparative HPLC (gradient separation 20 to 80% ACN in 40 min, flow rate 3 mL/min) provided conjugate **con2** as a purple solid (2.1 mg, 0.0014 mmol, 17.21%). M_theoritical_: 1524.78; (Μ_theoritical_ + H)^+^: 1525.78; found 1524.86; (Μ_theoritical_ + 2H^+^)/2: 763.39; found 763.27; (Figures S44 and S45).

#### 3.1.6. Synthesis of Anthraquinone—GnRH Conjugate **con8**

Analogue **3** (16.4 mg, 0.011 mmol, 1.0 eq) and analogue **13** (4.9 mg, 0.015 mmol, 1.4 eq) were dissolved in DMSO (0.4 mL) and DIPEA (0.015 mL). The reaction mixture was stirred at RT for 4 h and monitored via analytical RP-HPLC. Purification using semi-preparative HPLC (gradient separation 20 to 80% ACN in 40 min, flow rate 3 mL/min) provided conjugate **con8** as a purple solid (7.5 mg, 0.0043 mmol, 38.96%). M_theoritical_: 1749.82; (Μ_theoritical_ + H)^+^: 1750.82; found 1750.80; (Μ_theoritical_ + 2H^+^)/2: 875.91; found 876.31; (Μ_theoritical_ + 3H^+^)/3: 584.27; found 585.02; (Figures S46 and S47).

#### 3.1.7. Synthesis of Mitoxantrone—GnRH Conjugate **con7**

Analogue **3** (16.1 mg, 0.011 mmol, 1.0 eq) and analogue **16** (6.0 mg, 0.012 mmol, 1.1 eq) were dissolved in a high dilution of MeOH (6 mL). The reaction mixture was stirred at RT for 6 h and monitored via analytical RP-HPLC. Purification using semi-preparative HPLC (gradient separation 30 to 60% ACN in 40 min, flow rate 3 mL/min) provided conjugate **con7** as a blue solid (17.5 mg, 0.009 mmol, 81.89%). M_theoritical_: 1942.29; (Μ_theoritical_ + H)^+^: 1943.29; found 1942.52 (Μ_theoritical_ + 2H^+^)/2: 972.15; found 971.93 (Μ_theoritical_ + 3H^+^)/3: 648.43; found 648.56; (Μ_theoritical_ + 7H^+^)/7: 278.47; found 279.67; (Figures S57 and S58).

#### 3.1.8. Synthesis of Mitoxantrone—GnRH Conjugate **con3**

Analogue **2** (23.6 mg, 0.02 mmol, 1.0 eq) and analogue **18** (13.9 mg, 0.021 mmol, 1.1 eq) were dissolved in a high dilution of MeOH (7 mL). The reaction mixture was stirred at RT for 24 h and monitored via analytical RP-HPLC. Purification using preparative HPLC (gradient separation 30 to 40% ACN in 43 min, flow rate 12 mL/min) provided conjugate **con3** as a blue solid (11.7 mg, 0.0068 mmol, 33.82%). M_theoritical_: 1730.00; (Μ_theoritical_ + H)^+^: 1731.00; found 1729.86; (Μ_theoritical_ + 2H^+^)/2: 866.00; found 865.91; (Μ_theoritical_ + 3H^+^)/3: 577.67; found 577.79; (Figures S64 and S65).

### 3.2. Conformational Studies of ***con7*** and ***con3***

The peptides and the conjugates were designed using the USF Chimera 1.16 software [81]. The template for the GnRH backbone of the conjugates was derived from a previously reported [82] GnRH MD simulation, with the relevant changes implemented at position 6. Molecular dynamics calculations were performed for the peptide conjugates **con7** and **con3** and for the peptides which are present in these conjugates (analogue **4** and analogue **2**, respectively) using AMBER14 software [83]. All structures were optimized with the GAMESS R1 software [84,85] using the density-functional theory (DFT) [86,87] and the Hartree–Fock (HF) approximation. The B3LYP/6-311G atomic basis set was applied, while the convergence criterion was set at 0.0001 [87,88]. Detailed information on the construction of the parameters is provided in Supplementary Materials (see Sections S3 and S6).

### 3.3. GnRH Receptor Binding Assay of Synthesized Analogues

Iodination of the ^125^I-DTyr^6^-His^5^-GnRH: The radioiodination of GnRH peptide was performed following the chloramine-T method. Specifically, 5 μL of a 1 mg/mL DTyr^6^-His^5^-GnRH solution was diluted with 5 μL of iodination buffer (0.25 M phosphate, pH 7.4) and then mixed with 10 μL of 0.5 mg/mL chloramine-T solution in iodination buffer and 2.2 μL of Na^125^I solution corresponding to 1 mCi. After incubation for 1 min under continuous shaking, 20 μL of 1 mg/mL sodium metabisulfite solution in iodination buffer was added to stop the reaction. The radiolabeled peptide was purified via HPLC (gradient elution 3% to 50% ACN in 15 min, flow rate 1 mL/min).

Cell culture and membrane preparation: We prepared HEK 293 cells stably expressing the GnRH-R [89] grown in DMEM/F12 (1:1) containing 3.15 g/L glucose and 10% bovine calf serum at 37 ºC and 5% CO_2_. The cells were grown in 100 mm dishes to achieve 90–100% confluency on the day of the experiment. The cells were washed using phosphate-buffered saline (PBS) (4.3 mM Na_2_HPO_4_.7H_2_0, 1.4 mM KH_2_PO_4_, 137 mM NaCl, 2.7 mM KCl, pH 7.2–7.3 at RT), briefly treated with PBS containing 2 mM EDTA (PBS/EDTA), and then dissociated in PBS/EDTA. A cell suspension was centrifuged at 1000× *g* for 5 min at room temperature, and the pellet was homogenized in 1 mL of buffer O (50 mM Tris—HCl containing 0.5 mM EDTA, 10% sucrose, 10 mM MgCl_2_, pH 7.4 at 4 °C) using a Janke & Kunkel IKA Ultra Turrax T25 homogenizer (Staufen im Breisgau, Germany), at setting ~20, for 10–15 s, at 4 °C. The homogenate was centrifuged at 250× *g* for 5 min at room temperature. The pellet was discarded, and the supernatant was centrifuged at 16,000× *g*, for 10 min, at 4 °C. The membrane pellet was resuspended in buffer B (25 mM HEPES containing 1 mM CaCl_2_, 10 mM MgCl_2_, 0.5% BSA, pH 7.4 at 4 °C) (1 mL/100 mm dish) and used for radioligand binding studies.

^125^I-DTyr^6^-His^5^-GnRH binding: Aliquots of diluted membrane suspension (50 μL) were added into low-retention tubes containing buffer B and 120,000–240,000 cpm ^125^I-DTyr^6^-His^5^-GnRH, with or without increasing concentrations of GnRH analogues, in a final volume of 0.5 mL. The mixtures were incubated at 4 °C for 16–19 h and then filtered using a Brandel cell harvester through Whatman GF/C glass fiber filters and then presoaked for 1–2 h in 0.5% polyethylenimine at 4 °C. The filters were washed 4 times with 2 mL of ice-cold 50 mM Tris—HCl, pH 7.4 at 4 °C. Filters were assessed for radioactivity in a gamma counter (LKB Wallac 1275 minigamma, 80% efficiency). The amount of membrane used was adjusted to ensure that the specific binding was always equal to or less than 10% of the total concentration of the added radioligand. Specific ^125^I-Tyr^6^-His^5^-GnRH binding was defined as total binding less nonspecific binding in the presence of 1000 nM triptorelin. Data for competition binding were analyzed via nonlinear regression analysis using GraphPad Prism 4.0 (GraphPad Software, San Diego, CA, USA). IC_50_ values were obtained by fitting the data from competition studies into a one-site competition model.

### 3.4. Disulfide Bond Reduction Assays by EcoTrx1

The assay employed was based on changes in the absorbance at 340 nm from the oxidation of NADPH using a modified version of the protocol laid out in [90]. Briefly, the decrease in A_340_ was measured in 96-well plates containing 100 μL of 200 mM potassium phosphate pH 7.4, 1 mM EDTA, 0.1 mg/mL BSA, 200 μM NADPH, 2 μM *E. coli* Trx1 and 10 nM *E. coli* TrxR for 5 min at 25 °C with insulin at a final concentration of 0.067 mM (0.2 mM disulfides) (see Table S2). The reduction of **con7** was measured at a final concentration of 0.2 mM (see Table S3). Blanks were samples without EcoTrxR.

## 4. Conclusions

The design and synthesis of the GnRH conjugates presented in this study was based on chemical modifications to the AEZS-108 conjugate. All developed conjugates featured an anthraquinone-type cytotoxic compound tethered to a leuprolide analogue for enhanced binding efficiency. AEZS-108 initially held great promise as an anti-cancer agent; however, the premature release of doxorubicin in plasma [60], along with the resultant toxicity, led to the termination of clinical trials [91,92]. For that reason, the conjugates reported in this study were meticulously designed to ensure the selective release of their cytotoxic agent only upon binding to GnRHR I to cancer cells via the thioredoxin system. The linkage between the cytotoxic agent and the GnRH peptides was achieved through either a direct disulfide bond or the addition of a linker, as exemplified in **con3** and **con7,** respectively. Mitoxantrone analogues were selected as the cytotoxic compounds due to their favorable pharmacological profile in comparison to doxorubicin.

The evaluation of the synthesized conjugates (**con1**–**con8**) for their interaction with GnRHR I showed significant enhanced binding affinities compared to leuprolide. Notably, the highest binding affinities were predominantly observed with **con3** and **con7** (Figure 3), while the release of the cytotoxic agent, facilitated by the thioredoxin system after the reduction of the disulfide bond, was estimated in **con7** (Figure 4). Molecular dynamic (MD) simulations conducted for both conjugates **con7** and **con3** revealed that the conformation of these analogues exposed the disulfide bond to the surrounding solvent environment, rendering it susceptible to the thioredoxin system within cancer cells. Additionally, the simulations proved that these conjugates adopted U-shape/bent conformations, a characteristic feature closely associated with GnRH peptides and agonistic activity, which allowed their binding to GnRHR I [42].

The findings derived from our in vitro evaluation indicated that **con3** and **con7**, both mitoxantrone analogues, exhibited robust binding affinities for GnRHR I. These results suggested that these compounds could be considered promising candidates for further comprehensive pharmacological investigations, both in vitro and in vivo, in the ongoing battle against cancer. In conclusion, the outcomes presented in this paper underscore the potential of these novel GnRH conjugates as candidates for the development of new innovative drug carriers designed to selectively target cancer cells. More specifically, novel GnRH-mitoxantrone conjugates could be used in the treatment of hormone-dependent cancers that overexpress the GnRH receptors, including ovarian and endometrial disease. Before entering clinical studies, these conjugates should be studied in vivo for their ability to inhibit the proliferation and migration of cancer cells in combination with stability, toxicology and extensive pharmacological evaluation.

## Data Availability

Data is contained within the article or Supplementary Materials.

## Supplementary Materials

The following supporting information can be downloaded at: https://www.mdpi.com/article/10.3390/ijms242015232/s1.
