# Supplementary material for: Rational Design, Synthesis and Binding Affinity Studies of Anthraquinone Derivatives Conjugated to Gonadotropin-Releasing Hormone (GnRH) Analogues towards Selective Immunosuppression of Hormone-Dependent Cancer
Source: Int J Mol Sci. 2023 Oct 16;24(20):15232. doi: 10.3390/ijms242015232 (PMC10607160; doi:10.3390/ijms242015232)
Supplement: Supplementary file 1 [file ijms-24-15232-s001.zip › ijms-2602932-supplementary.pdf]

Rational design, synthesis and binding affinity studies of anthraquinone derivatives conjugated to gonadotropin releasing hormone (GnRH) analogues towards selective immunosuppression of hormone dependent cancer

Georgia Biniari[a#], Christos Markatos[b#], Agathi Nteli[a], Haralambos Tzoupis[a], Carmen Simal[a], Alexios Vlamis-Gardikas[a], Vlasios Karageorgos[b], Ioannis Pirmettis[c], Panagiota Petrou[c], Maria Venihaki[d], George Liapakis[*, b] and Theodore Tselios[*, a]

[a]*Department of Chemistry, University of Patras, GR-26504, Rion, Greece*

[b]*Department of Pharmacology, School of Medicine, University of Crete, Heraklion, Greece.*

[c]*Institute of Nuclear & Radiological Sciences & Technology, Energy & Safety, National Centre for Scientific Research "Demokritos", Athens, Greece.*

[d]*Departmernt of Clinical Chemistry, School of Medicine, University of Crete, Heraklion, Greece.*

Corresponding authors: Theodore Tselios, ttselios@upatras,gr; George Liapakis, liapakig@uoc.gr.

Table of Contents

S.1 Peptide synthesis **4**

*S.1.1 Synthesis of peptide [DCys^{6}]GnRH (analogue **1**)* **4**

*S.1.2 Synthesis of peptide [DCys^{6}, Pro^{9}-NHEt]GnRH (analogue **2**)* **6**

*S.1.3 Synthesis of peptide [DLys(Ahx-PDP)^{6}, Pro^{9}-NHEt]GnRH (analogue **3**)* 7

*S.1.4 Synthesis of peptide [DLys^{6}, Pro^{9}-NHEt]GnRH (analogue **4**)* **9**

*S.1.5 Synthesis of peptide [DLeu^{6}, Pro^{9}-NHEt]GnRH (**Leuprolide**)* **11**

S.2 Synthesis of anthraquinone and mitoxantrone GnRH analogues **12**

*S.2.1 Synthesis of anthraquinone - GnRH conjugates (**con4; con5; con6**)* **12**

*S.2.1.1 Synthesis of anthraquinone analogue **5*** **12**

*S.2.1.2 Synthesis of anthraquinone analogue **6*** **14**

S.2.1.3 Synthesis of anthraquinone analogue 7 .... **16**

*S.2.1.4 Synthesis of anthraquinone analogue **8*** **18**

*S.2.1.5 Data of anthraquinone - GnRH conjugate **con4*** **19**

*S.2.1.6 Data of anthraquinone - GnRH conjugate **con5*** **20**

*S.2.1.7 Data of anthraquinone - GnRH conjugate **con6*** **21**

*S.2.2 Synthesis of anthraquinone - GnRH conjugates (**con1; con2; con8**)* **22**

*S.2.2.1 Synthesis of analogue **9*** **22**

*S.2.2.2 Synthesis of analogue **10*** **24**

*S.2.2.3 Synthesis of analogue **11*** **25**

*S.2.2.4 Synthesis of anthraquinone analogue **12*** **27**

*S.2.2.5 Synthesis of anthraquinone analogue **13*** **29**

*S.2.2.6 Data of anthraquinone - GnRH conjugate **con1*** **31**

*S.2.2.7 Data of anthraquinone - GnRH conjugate **con2*** **32**

*S.2.2.8 Data of anthraquinone - GnRH conjugate **con8*** **33**

*S.2.3 Synthesis of mitoxantrone - GnRH conjugate (**con7; con3**)* **34**

*S.2.3.1 Synthesis of mitoxantrone - GnRH conjugate (**con**7)* ***34***
*S.2.3.1.1 Synthesis of mitoxantrone analogue **14*** ***34***
*S.2.3.1.2 Synthesis of mitoxantrone analogue **15*** ***37***
*S.2.3.1.3 Synthesis of mitoxantrone analogue **16*** ***39***
*S.2.3.1.4 Data of mitoxantrone- GnRH conjugate **con**7* ***41***
*S.2.3.2 Synthesis of mitoxantrone - GnRH conjugate (**con3**)* ***42***
*S.2.3.2.1 Synthesis of mitoxantrone analogue **1**7* ***42***
*S.2.3.2.2 Synthesis of mitoxantrone analogue **18*** ***44***
*S.2.3.2.3 Data of mitoxantrone- GnRH conjugate **con3*** ***46***

*S.3 Conformational studies of **con**7 and **con3*** *47*

*S.4 Disulfides reduction of **con**7 and insulin by EcoTrx1* ***50***

*S.5 Binding affinity studies of mitoxantrone, [DLys^6, Pro^9-NHEt]GnRH (analogue **4**) and [DCys^6, Pro^9-NHEt]GnRH (analogue **2**)* ***52***

S.6 References ***54***

S.1 Peptide synthesis

S.1.1 Synthesis of peptide [DCys^6]GnRH (analogue 1)

[DCys^6]GnRH (analogue **1**) was prepared by conventional Solid Phase Peptide Synthesis (SPPS), following the Fmoc/*t*Bu methodology on CLTR-Cl resin (1 g). The N^{α}-9-Fluorenylmethyloxycarbonyl (Fmoc) protected linker (Fmoc-Rink$_{amide}$ linker) was firstly esterified to the resin in the presence of diisopropylethylamine (DIPEA) in CH_2Cl_2 for 1.5 h at RT. The peptide sequence was assembled by sequential couplings of Fmoc-protected amino acids in the presence of *N, N'*-diisopropylcarbodiimide (DIC) and 1-hydroxybenzotriazole (HOBt) in dimethylacetamide (DIMAC), followed by Fmoc deprotection with 25% piperidine solution in dimethylformamide (DMF). The completion of each coupling and Fmoc deprotection step was verified by Kaiser test and thin layer chromatography (TLC). Once peptide synthesis was completed, the resin was cleaved with CH_2Cl_2 / 2,2,2-trifluoroethanol (TFE) (7:3) for 2 h at RT. The removal of side chains protecting groups was accomplished by a trifluoroacetic acid (TFA) / Anisole / H_2O (95:4:1) solution for 5 h at RT. Purification and identification were achieved on semi-preparative HPLC (gradient separation from 20 to 60% ACN in 40 min; flow rate 3 ml/min) and ESI-MS, respectively and afforded analogue **1** as a white solid (10.6 mg, 0.0086 mmol).

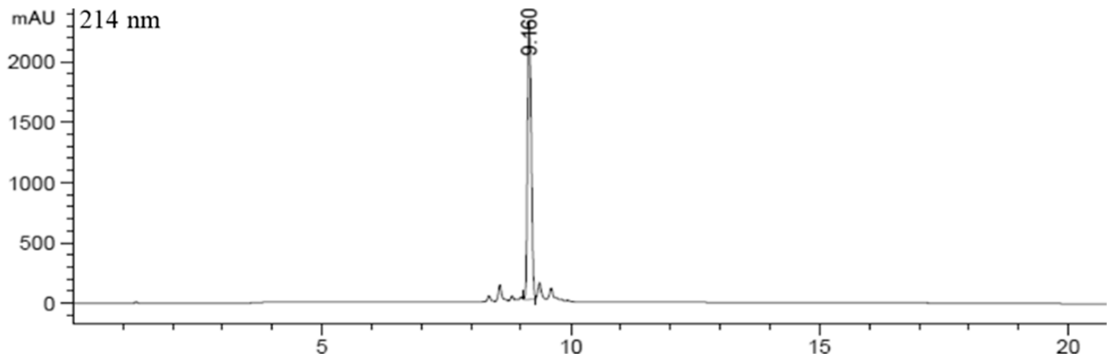

Figure S1: Analytical RP-HPLC chromatogram of synthesized analogue **1** at 214 nm.

RP-HPLC Conditions:

i) Column: Agilent ZORBAX Eclipse Plus C18 (3.5 μm, 100 x 4.6 mm), flow rate 1 ml/min,

ii) Solvents: H_2O (0.08 % TFA), ACN (0.08 % TFA),

iii) Gradient elution: from 10 % ACN to 100 % ACN in 30 min at RT,

iv) t_R: 9.160 min; Purity: 93 %.

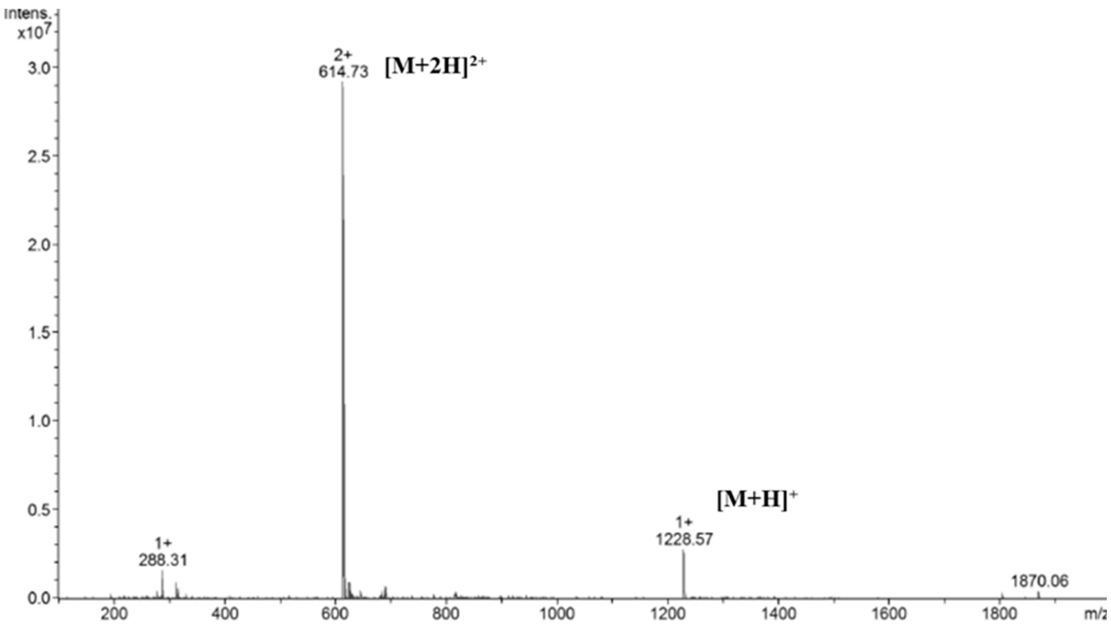

Figure S2: ESI-MS of synthesized analogue **1**. $M_{theoritical}$: 1228.40; $(M_{theoritical} + H)^+$: 1229.40; found 1228.57; $(M_{theoritical} + 2H^+)/2$: 615.20; found 614.73.

S.1.2 Synthesis of peptide [DCys^{6}, Pro^{9}-NHEt]GnRH (analogue 2)

[DCys^{6}, Pro^{9}-NHEt]GnRH (analogue **2**) was prepared by SPPS, following the Fmoc/*t*Bu methodology (see S.1.1) on Ethyl Indole AM resin (1 g). The resin was cleaved with TFA / CH_2Cl_2 (1:1) solution for 1 h at RT. The removal of side chains protecting groups was further accomplished by a TFA / CH_2Cl_2 / Anisole / H_2O (90:5:2.5:2.5) solution for 4 h at RT. Purification and identification were achieved on semi-preparative HPLC (gradient separation from 20 to 60% ACN in 40 min, flow rate 3 ml/min) and ESI-MS, respectively and afforded analogue **2** as a white solid (18.7 mg, 0.016 mmol).

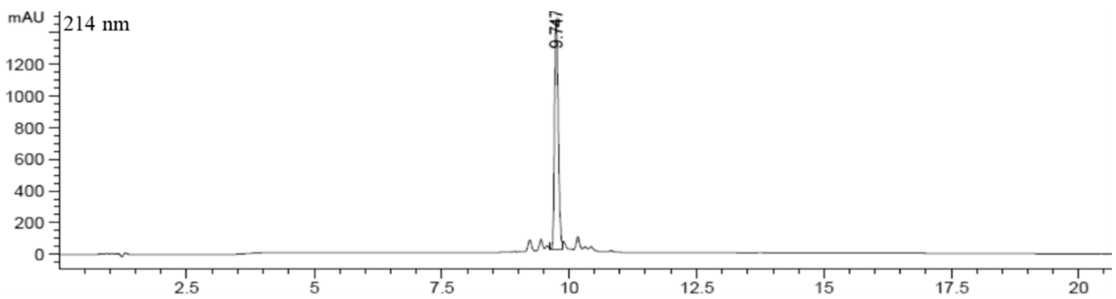

Figure S3: Analytical RP-HPLC chromatogram of synthesized analogue **2** at 214 nm.
RP-HPLC Conditions:
i) Column: Agilent ZORBAX Eclipse Plus C18 (3.5 μm, 100 x 4.6 mm), flow rate 1 ml/min,
ii) Solvents: H_2O (0.08 % TFA), ACN (0.08 % TFA),
iii) Gradient elution: from 10 % ACN to 100 % ACN in 30 min at RT,
iv) t_R: 9.747 min; Purity: 92 %.

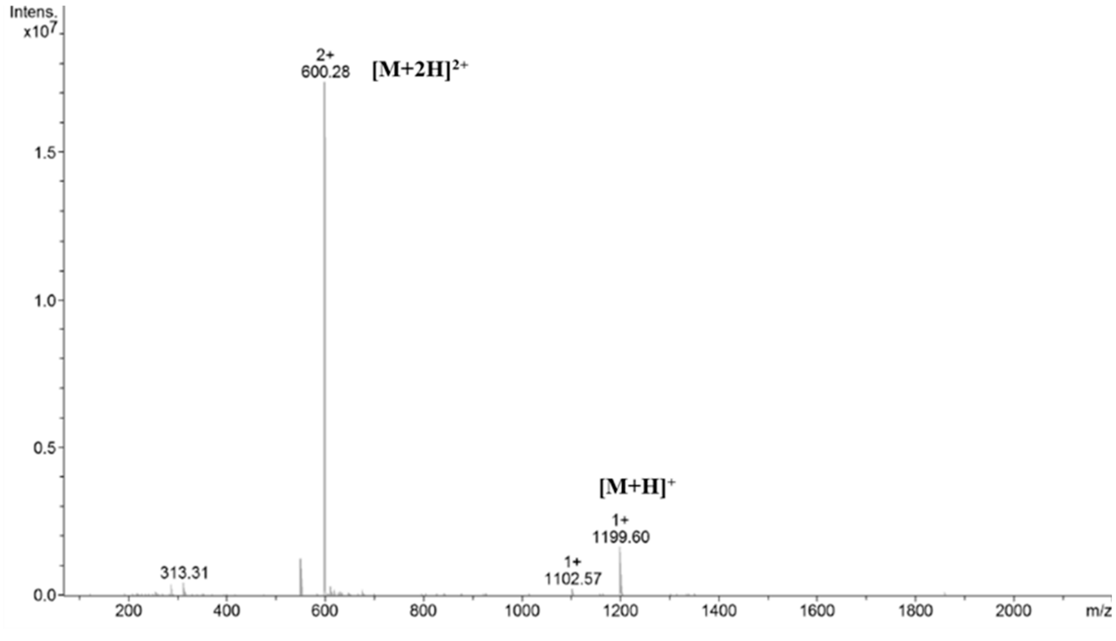

Figure S4: ESI-MS of synthesized analogue **2**. $M_{theoritical}$: 1199.40; $(M_{theoritical} + H)^{+}$: 1200.40; found 1199.60; $(M_{theoritical} + 2H^{+})/2$: 600.70; found 600.28.

S.1.3 Synthesis of peptide [DLys(Ahx-PDP)[6], Pro[9]-NHEt]GnRH (analogue 3)

[DLys(Ahx-PDP)[6], Pro[9]-NHEt]GnRH (analogue **3**) was prepared by conventional SPPS, following the Fmoc/*t*Bu methodology (see S.1.1) on Ethyl Indole AM resin (1 g). The 4-methyltrityl(Mtt)-protecting group was removed selectively from the side chain of DLys with a CH_2Cl_2 / 1,1,1,3,3,3- hexafluoro-2-propanol (HFIP) (4:1) solution. Subsequently, 6-aminohexanoic acid was coupled to succinimidyl 3-(2-pyridyldithio) propionate and this analogue was attached to the amine group of DLys, in the presence of DIC / HOBt / DIPEA in DIMAC. The coupling was monitored by Kaiser test. The resin was cleaved with TFA / CH_2Cl_2 (1:1) solution for 1 h at RT and the removal of side chains protecting groups was achieved by a TFA / CH_2Cl_2 / Anisole / H_2O (90:7:2:1) solution for 4 h at RT. Purification and identification were achieved on semi-preparative HPLC (gradient separation from 30 to 60% ACN in 40 min, flow rate 3 ml/min) and ESI-MS, respectively and afforded analogue **3** as white solid (6.0 mg, 0.0039 mmol).

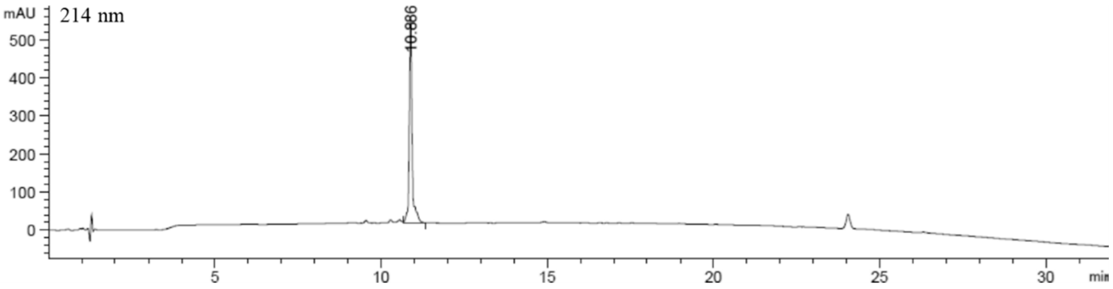

Figure S5: Analytical RP-HPLC chromatogram of synthesized analogue **3** at 214 nm.

RP-HPLC Conditions:

i) Column: Agilent ZORBAX Eclipse Plus C18 (3.5 μm, 100 x 4.6 mm), flow rate 1 ml/min,

ii) Solvents: H_2O (0.08 % TFA), ACN (0.08 % TFA),

iii) Gradient elution: from 10 % ACN to 100 % ACN in 30 min at RT,

iv) t_R: 10.886 min; Purity: 97 %.

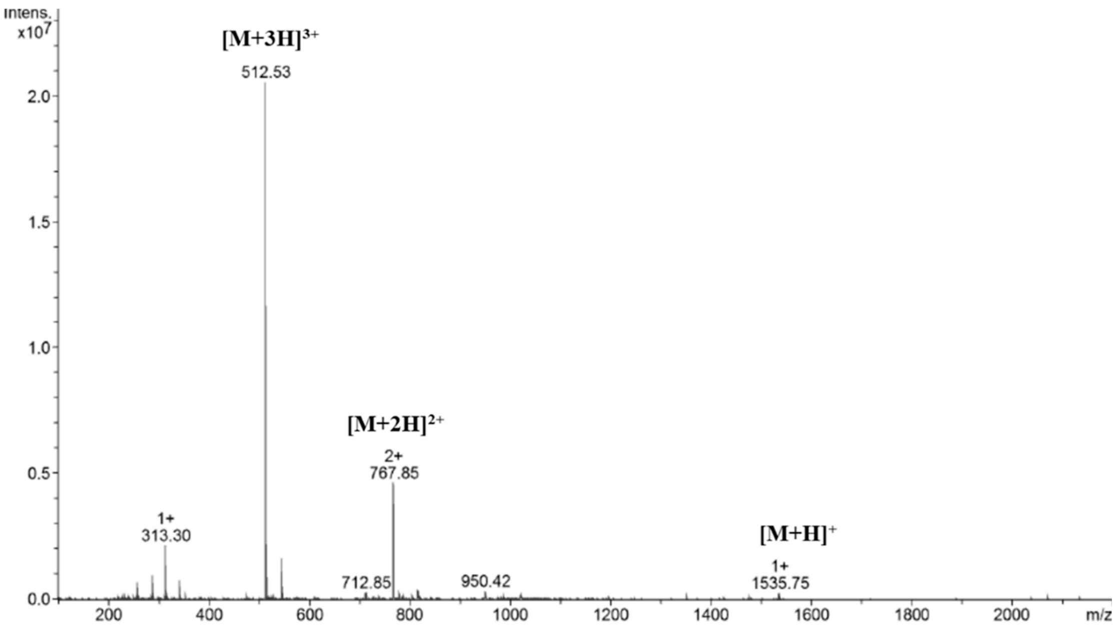

Figure S6: ESI-MS of synthesized analogue **3**. $M_{theoritical}$: 1534.87; $(M_{theoritical} + H)^+$: 1535.87; found 1535.75; $(M_{theoritical} + 2H^+)/2$: 768.44; found 767.85; $(M_{theoritical} + 3H^+)/3$: 512.62; found 512.53.

S.1.4 Synthesis of peptide [DLys^6, Pro^9-NHEt]GnRH (analogue 4)

[DLys^6, Pro^9-NHEt]GnRH (analogue **4**) was prepared by conventional SPPS, following the Fmoc/*t*Bu methodology (see S.1.1) on CLTR-Cl resin (2 g). The resin was cleaved with CH_2Cl_2 / 2,2,2-trifluoroethanol (TFE) (6:4) for 1 h at RT. Subsequently, the modification of the carboxyl-terminal was achieved in the presence of ethylamine hydrochloride ($EtNH_2 \cdot HCl$) (100 μL, 1.42 mmol, 10.0 eq), TBTU (0.14 g, 0.43 mmol, 3.0 eq) and DIPEA (0.62 ml, 3.54 mmol, 25.0 eq) in CH_2Cl_2 for 16 h at 30°C and was monitored by analytical RP-HPLC. The removal of side chains protecting groups was accomplished by a TFA / CH_2Cl_2 / Anisole / H_2O (90:7:2:1) for 5 h at RT. Purification and identification were achieved on preparative HPLC (gradient separation from 20 to 70% ACN in 43 min; flow rate 12 ml/min) and ESI-MS, respectively and afforded **analogue 4** as a white solid (53 mg, 0.043 mmol).

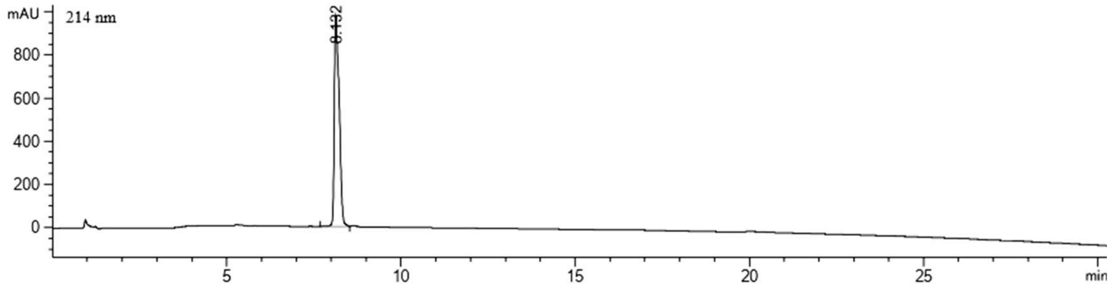

Figure S7: Analytical RP-HPLC chromatogram of synthesized analogue **4** at 214 nm.

RP-HPLC Conditions:

i) Column: Agilent ZORBAX Eclipse Plus C18 (3.5 μm, 100 x 4.6 mm), flow rate 1 ml/min,

ii) Solvents: H_2O (0.08 % TFA), ACN (0.08 % TFA),

iii) Gradient elution: from 10 % ACN to 100 % ACN in 30 min at RT,

iv) t_R: 8.132 min; Purity: 98 %.

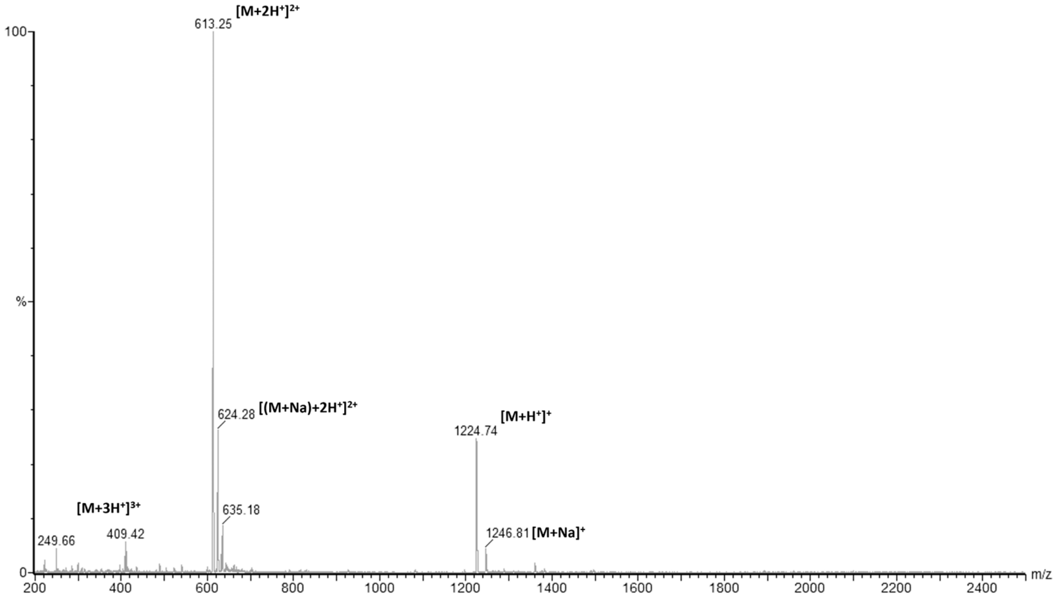

Figure S8: ESI-MS of synthesized analogue **4**. $M_{theoritical}$: 1224.44; ($M_{theoritical}$ + H)+: 1225.44; found 1224.74; ($M_{theoritical}$ + Na)+: 1247.44; found 1246.81; ($M_{theoritical}$ + 2H+)/2: 613.22; found 613.25; [($M_{theoritical}$ + Na) + 2H+]/2: 624.72; found 624.28; ($M_{theoritical}$ + 3H+)/3: 409.15; found 409.42.

S.1.5 Synthesis of peptide [DLeu^6, Pro^9-NHEt]GnRH (Leuprolide)

[DLeu^6, Pro^9-NHEt]GnRH (**Leuprolide**) was prepared by conventional SPPS, following the Fmoc/*t*Bu methodology (see S.1.1) on CLTR-Cl resin (1 g). The resin was cleaved with CH_2Cl_2 / 2,2,2-trifluoroethanol (TFE) (7:3) for 1 h at RT. Subsequently, the modification of the carboxyl-terminal was achieved in the presence of ethylamine hydrochloride ($EtNH_2 \cdot HCl$) (87.8 μL, 1.30 mmol, 10.0 eq), TBTU (0.13 g, 0.39 mmol, 3.0 eq) and DIPEA (0.57 ml, 3.24 mmol, 25.0 eq) in CH_2Cl_2 for 24 h at RT and was monitored by analytical RP-HPLC. The removal of side chains protecting groups was accomplished by a TFA / CH_2Cl_2 (1:1) solution (6 ml), 4 drops of Anisole and 2 drops of H_2O for 5 h at RT. Purification and identification were achieved on semi-preparative HPLC (gradient separation from 20 to 60% ACN in 40 min; flow rate 3 ml/min) and ESI-MS, respectively and afforded **Leuprolide** as a white solid (13.5 mg, 0.011 mmol).

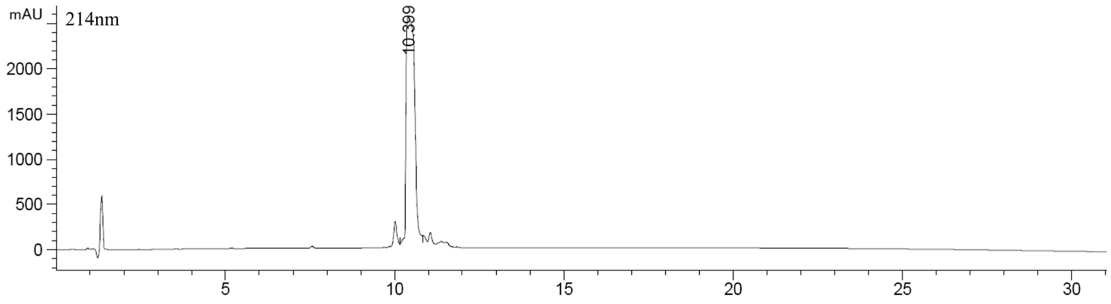

Figure S9: Analytical RP-HPLC chromatogram of synthesized Leuprolide at 214 nm.
RP-HPLC Conditions:
i) Column: Agilent ZORBAX Eclipse Plus C18 (3.5 μm, 100 x 4.6 mm), flow rate 1 ml/min,
ii) Solvents: H_2O (0.08 % TFA), ACN (0.08 % TFA),
iii) Gradient elution: from 10 % ACN to 100 % ACN in 30 min at RT,
iv) t_R: 10.399 min; Purity: 94.6 %.

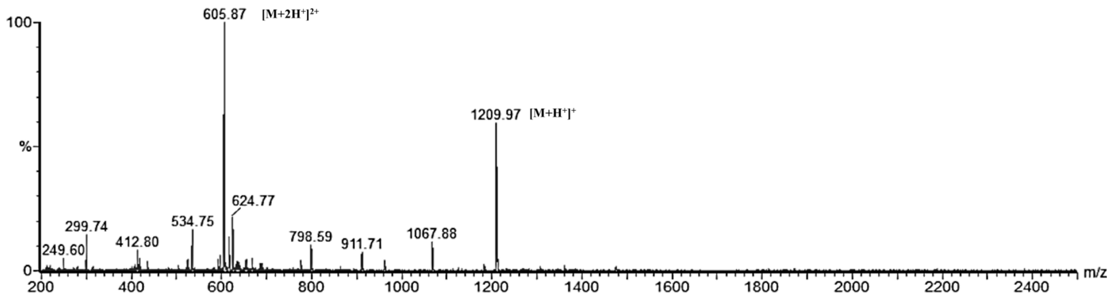

Figure S10: ESI-MS of synthesized **Leuprolide**. $M_{theoritical}$: 1209.42; $(M_{theoritical} + H)^+$: 1210.42; found 1209.97; $(M_{theoritical} + 2H^+)/2$: 605.70; found 605.87.

S.2. Synthesis of anthraquinone and mitoxantrone GnRH analogues

S.2.1 Synthesis of anthraquinone - GnRH conjugates (con4; con5; con6)

S.2.1.1 Synthesis of anthraquinone analogue 5

To a solution of commercially available leucoquinizarin (3.80 g, 15.69 mmol, 3.0 eq) in deoxygenated EtOH (70 ml), under argon at 50°C, was added dropwise a solution of commercially available *N*-Boc-1,4-butadiamine (1 ml, 5.23 mmol, 1.0 eq) in deoxygenated EtOH (10 ml) over a period of 1 h. The reaction mixture was stirred under reflux for 3 hours and at RT for a further16 h. The reaction mixture was monitored by TLC (2% MeOH in CH_2Cl_2) until completion. Then, it was left under air bubbling oxidation for 3 h. The solvent was evaporated under reduced pressure, and the crude product was precipitated by petroleum ether, and filtered under pressure. Purification by column chromatography on silica gel (2% MeOH in CH_2Cl_2) afforded starting (aminoalkyl)-anthraquinone **5** as purple solid (240 mg, 0.58 mmol, 11.16%).

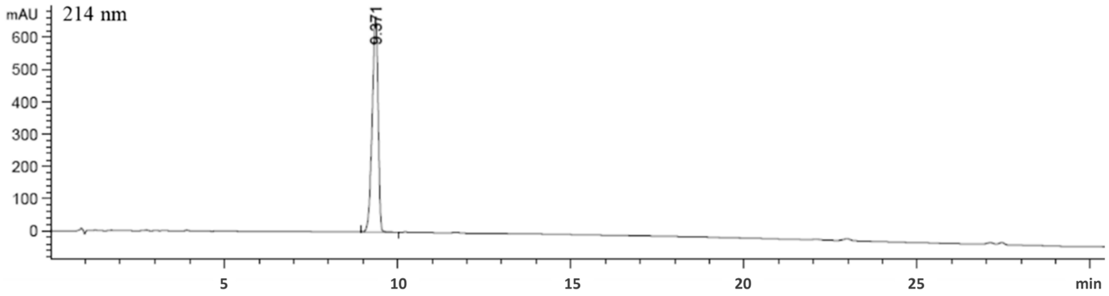

Figure S11: Analytical RP-HPLC chromatogram of synthesized analogue **5** at 214 nm.

RP-HPLC Conditions:

i) Column: Agilent ZORBAX Eclipse Plus C18 (3.5 μm, 100 x 4.6 mm), flow rate 1 ml/min,

ii) Solvents: H_2O (0.08 % TFA), ACN (0.08 % TFA),

iii) Gradient elution: from 60 % ACN to 100 % ACN in 30 min at RT,

iv) t_R: 9.371 min; Purity: 99 %.

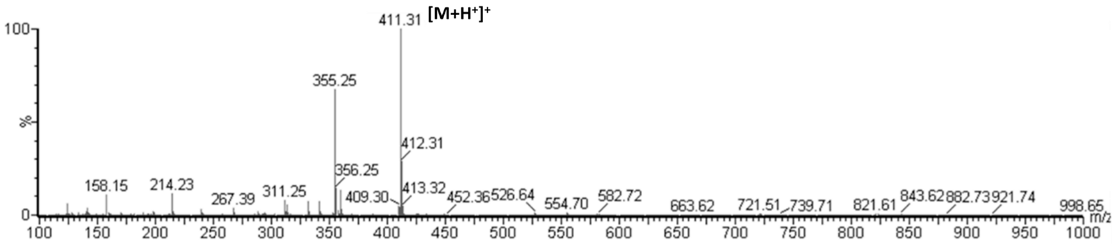

Figure S12: ESI-MS of synthesized analogue **5**. $M_{theoritical}$: 410.47; $(M_{theoritical} + H)^+$: 411.47; found 411.31.

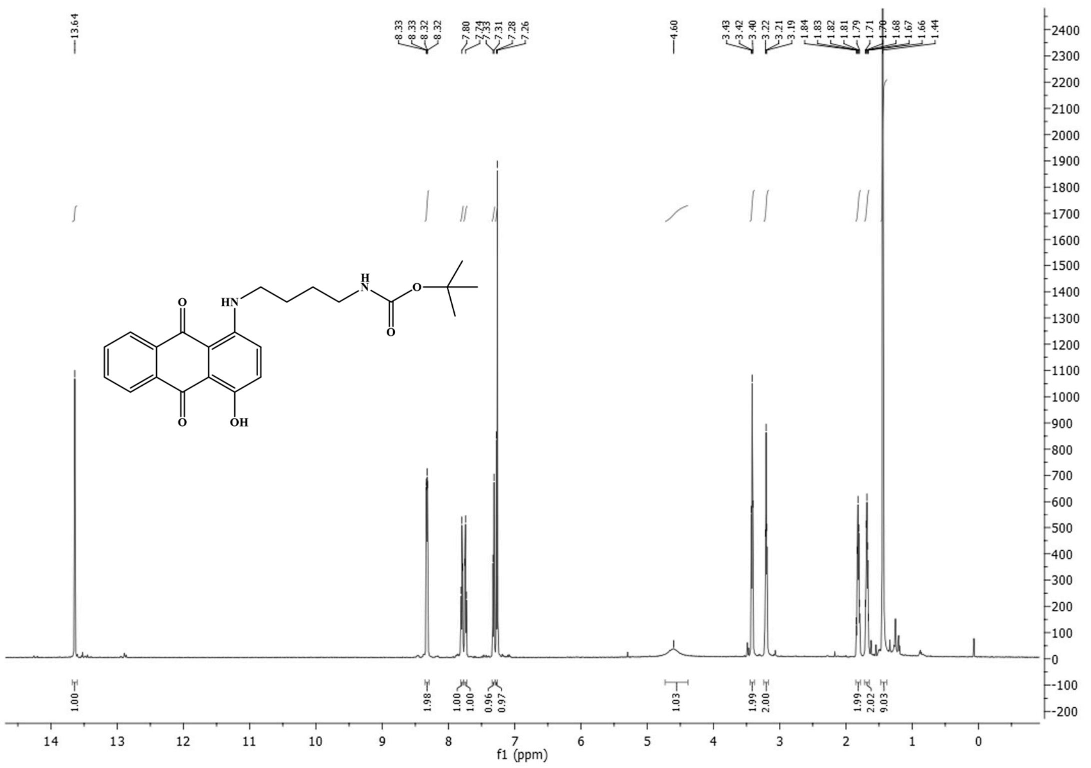

Figure S13: ^{1}H-NMR of synthesized analogue **5**. ^{1}H-NMR ($CDCl_3$, 600 MHz) δ 13.64 (s, 1H, OH), 8.33 – 8.32 (dd, *J* = 7.5, 2.0 Hz, 2H, CH^{Ar}), 7.80 (t, *J* = 7.35 Hz, 1H, CH^{Ar}), 7.74 (t, *J* = 7.35 Hz, 1H, CH^{Ar}), 7.33 (d, *J* = 9.95 Hz, 1H, CH^{Ar}), 7.28 (d, *J* = 9.95 Hz, 1H, CH^{Ar}), 4.60 (br s, 1H, NH), 3.42 (t, *J* = 6.9 Hz, 2H, CH_2NH), 3.21 (t, *J* = 6.9 Hz, 2H, CH_2NH), 1.84 – 1.79 (m, 2H, CH_2), 1.71 – 1.66 (m, 2H, CH_2), 1.44 (s, 9H, Boc).

S.2.1.2 Synthesis of anthraquinone analogue 6

Subsequently, analogue **5** (240mg) was dissolved in a mixture of TFA/ CH_2Cl_2 50:50 v:v (2 ml) and stirred in RT for 2 h for the removal of the *N*-Boc protecting group. Solvents were evaporated under reduced pressure to afford purple solid **6** as ammonium trifluoroacetate salt (140 mg, 0.45 mmol, 77.78%), which was used in the next step without further purification.

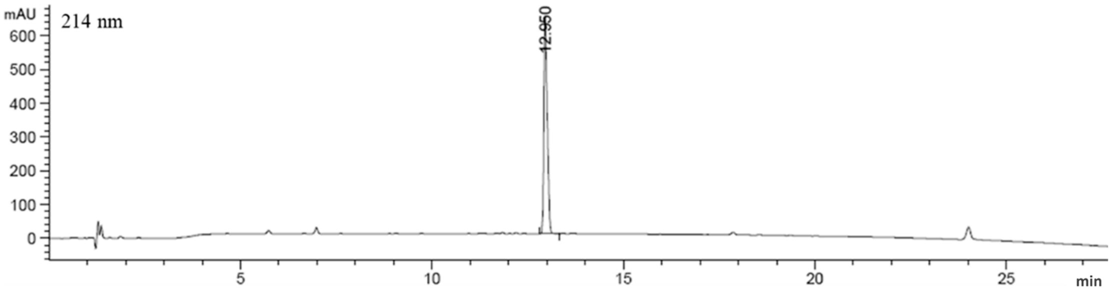

Figure S14: Analytical RP-HPLC chromatogram of synthesized analogue **6** at 214 nm.

RP-HPLC Conditions:

i) Column: Agilent ZORBAX Eclipse Plus C18 (3.5 μm, 100 x 4.6 mm), flow rate 1 ml/min,

ii) Solvents: H_2O (0.08 % TFA), ACN (0.08 % TFA),

iii) Gradient elution: from 10 % ACN to 100 % ACN in 30 min at RT,

iv) t_R: 12.950 min.

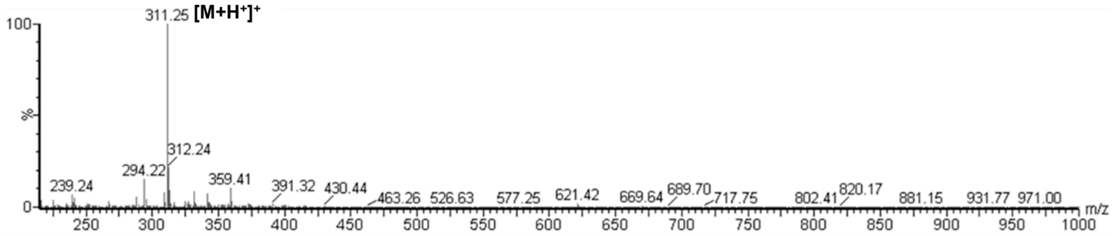

Figure S15: ESI-MS of synthesized analogue **6**. $M_{theoritical}$: 310.35; $(M_{theoritical} + H)^+$: 311.35; found 311.25.

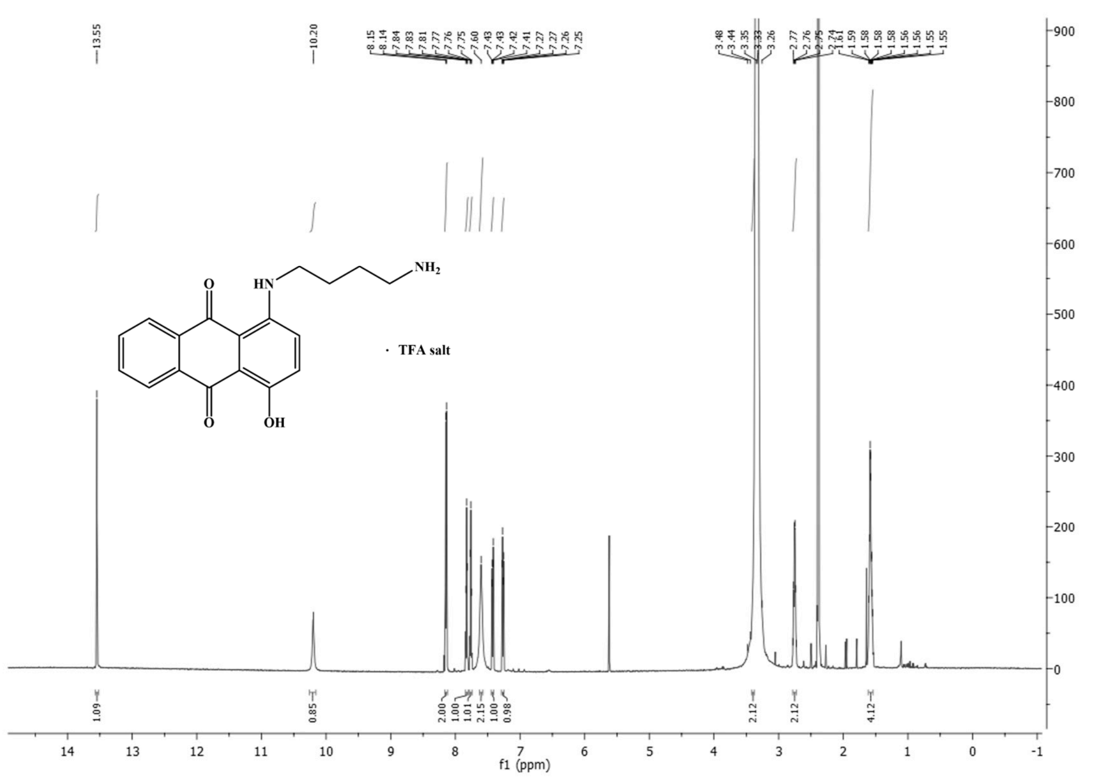

Figure S16: ^{1}H-NMR of synthesized analogue **6**. ^{1}H-NMR (d^{6}-DMSO, 600 MHz) δ 13.55 (s, 1H, OH), 10.20 (br s, 1H, NH), 8.14 (d, *J* = 7.5 Hz, 2H, CH^{Ar}), 7.82 (t, *J* = 7.5 Hz, 1H, CH^{Ar}), 7.76 (t, *J* = 7.5 Hz, 1H, CH^{Ar}), 7.60 (br s, 2H, NH_2), 7.43 – 7.41 (m, 1H, CH^{Ar}), 7.27 – 7.25 (m, 1H, CH^{Ar}), 3.48 – 3.26 (m, 2H, CH_2NH), 2.78 – 2.74 (m, 2H, CH_2NH_2), 1.61 – 1.55 (m, 4H, CH_2).

S.2.1.3 Synthesis of anthraquinone analogue 7

Analogue **6** was then treated with a solution of DCC/DMAP *in* CH_2Cl_2 in the presence of *S*-trityl-mercaptoacetic acid for the formation of an amide bond. Thus, it was initially dissolved (**6**, 100 mg, 0.33 mmol, 1.0 eq) in a round bottom flask **A** in the minimum volume of CH_2Cl_2 and then was added DMAP (44 mg, 0.36 mmol, 1.1 eq). In a round bottom flask **B** were dissolved *S*-trityl-mercaptoacetic acid (140.5 mg, 0.42 mmol, 1.3 eq), DMAP (48.9 mg, 0.40 mmol, 1.2 eq) and DCC (78.4 mg, 0.38 mmol, 1.15 eq) in CH_2Cl_2 (2 ml). The solution from **B** was added dropwise in **A** and the reaction mixture was stirred at RT for 5 h and monitored by TLC (20% MeOH-CH_2Cl_2) until completion. The solvent was evaporated under reduced pressure and purification by column chromatography on silica gel (1% MeOH-CH_2Cl_2) afforded analogue **7** as a purple solid (76 mg, 0.12 mmol, 36.75%).

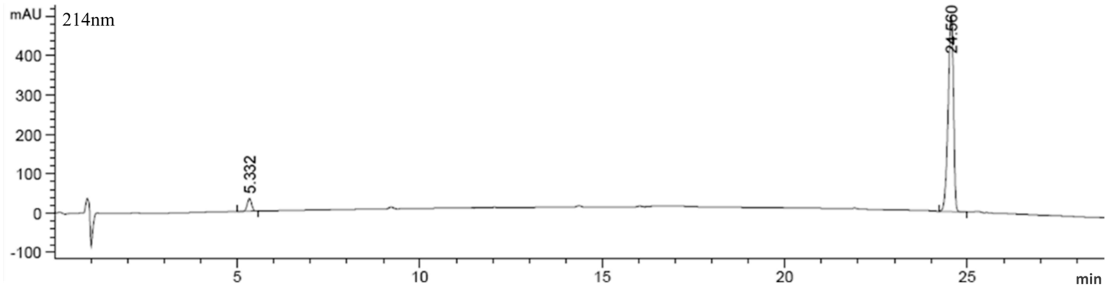

Figure S17: Analytical RP-HPLC chromatogram of synthesized analogue **7** at 214 nm.
RP-HPLC Conditions:
i) Column: Agilent ZORBAX Eclipse Plus C18 (3.5 μm, 100 x 4.6 mm), flow rate 1 ml/min,
ii) Solvents: H_2O (0.08 % TFA), ACN (0.08 % TFA),
iii) Gradient elution: from 40 % ACN to 100 % ACN in 30 min at RT,
iv) t_R: 24.560 min; Purity: 94 %.

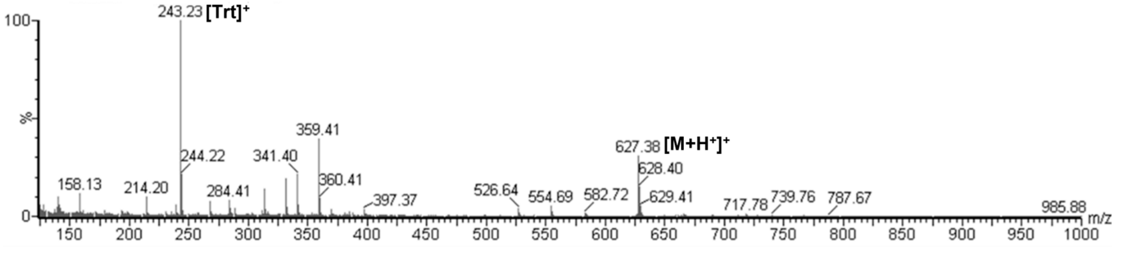

Figure S18: ESI-MS of synthesized analogue **7**. $M_{theoritical}$: 626.77; $(M_{theoritical} + H)^+$: 627.77; found 627.38; $(Trt)^+$: 243.12; found 243.23.

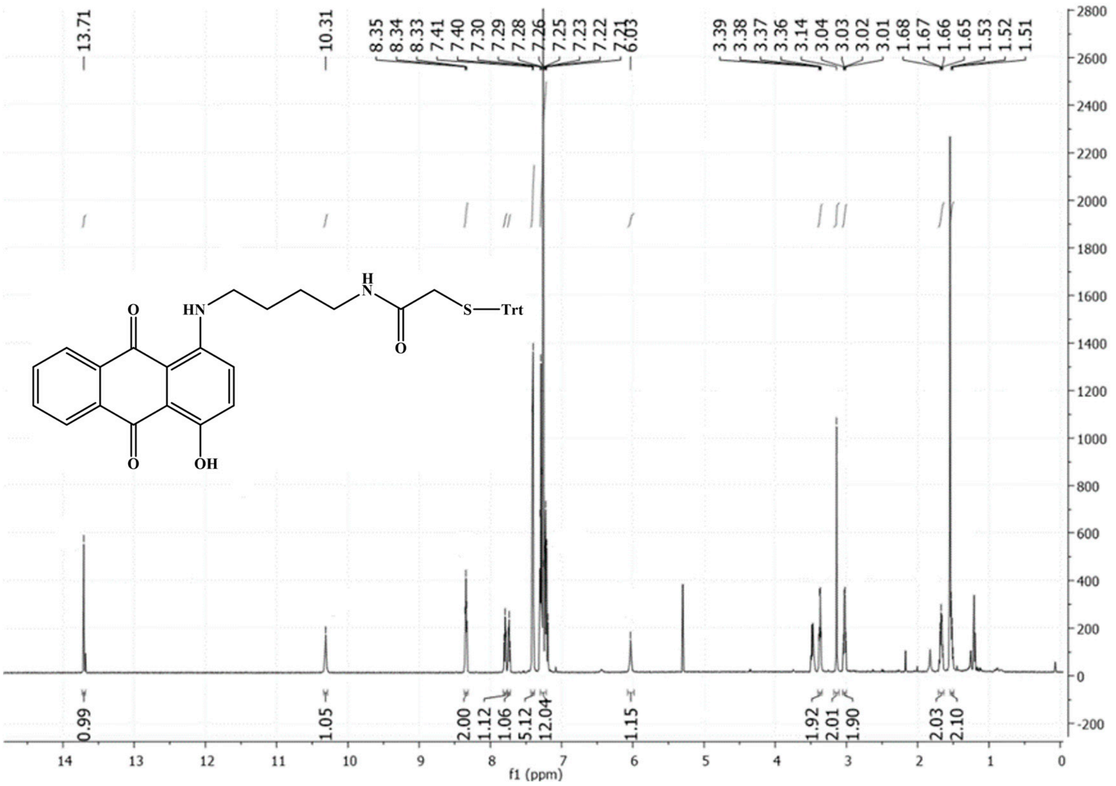

Figure S19: ^{1}H-NMR of synthesized analogue **7**. ^{1}H-NMR ($CDCl_3$, 600 MHz): δ 13.71 (s, 1H, OH), 10.31 (s, 1H, NH), 8.35 – 8.33 (m, 2H, CH^{Ar}), 7.79 (t, *J* = 6.3 Hz, 1H, CH^{Ar}), 7.74 (t, *J* = 7.1 Hz, 1H, CH^{Ar}), 7.40 (m, 5H, CH^{Ar}), 7.30 – 7.21 (m, 12H, CH^{Ar}), 6.03 (s, 1H, NH), 3.39 – 3.36 (m, 2H, CH_2), 3.14 (s, 2H, CH_2STrt), 3.04 – 3.01 (m, 2H, CH_2), 1.68 – 1.65 (m, 2H, CH_2), 1.54 – 1.51 (m, 2H, CH_2).

S.2.1.4 Synthesis of anthraquinone analogue 8

Final removal of trityl(Trt)-protecting group from analogue **7** (48 mg, 0.077 mmol) was achieved by treatment with a mixture of TFA:CH_2Cl_2:TES 94:3:3 (2 ml) at RT for 3 h. Solvents were removed under reduced pressure and the crude reaction mixture was purified by semi-preparative RP-HPLC (gradient separation from 50 to 100% ACN in 40 min, flow rate 3ml/min) to provide analogue **8** as a purple solid (25 mg, 0.065 mmol, 19.71%)

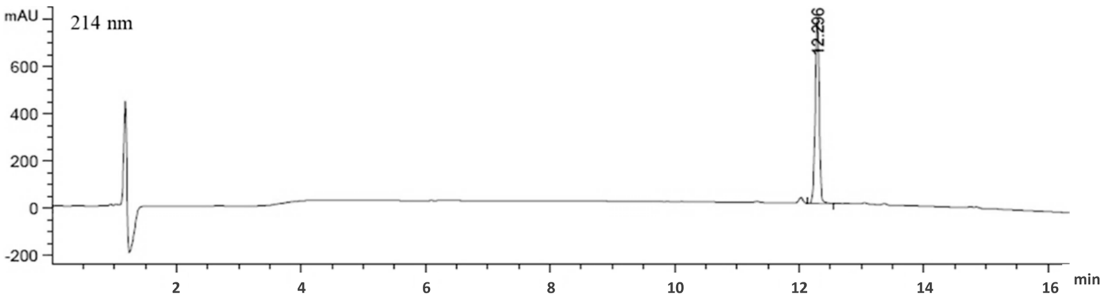

Figure S20: Analytical RP-HPLC chromatogram of synthesized analogue **8** at 214 nm.
RP-HPLC Conditions:
i) Column: Agilent ZORBAX Eclipse Plus C18 (3.5 µm, 100 x 4.6 mm), flow rate 1 ml/min,
ii) Solvents: H_2O (0.08 % TFA), ACN (0.08 % TFA),
iii) Gradient elution: from 10 % ACN to 100 % ACN in 15 min at RT,
iv) t_R: 12.296 min; Purity: 97 %.

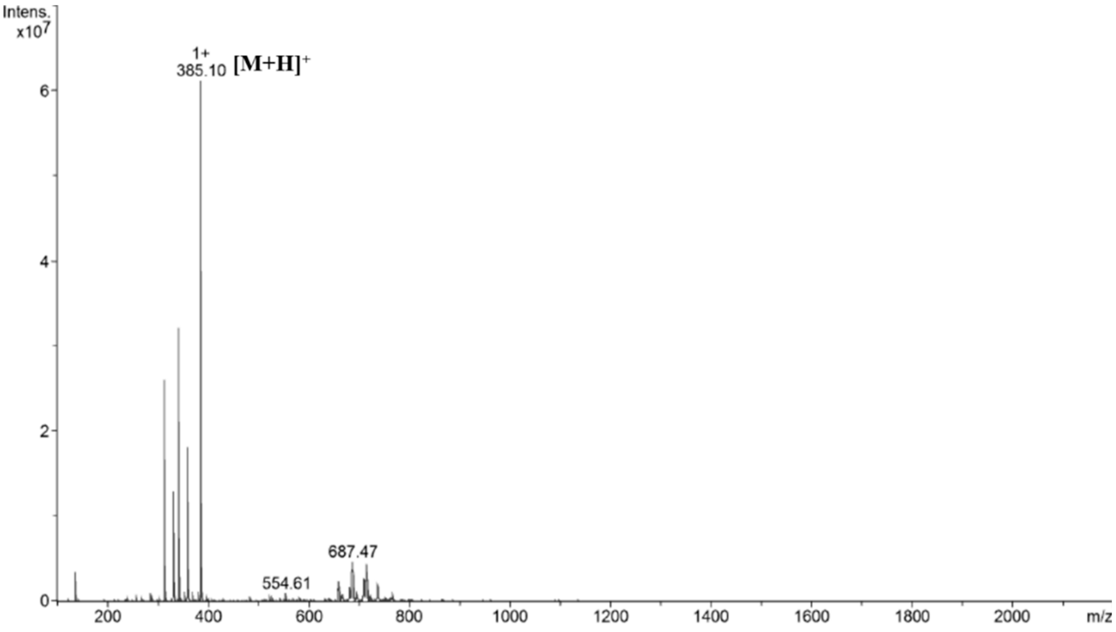

Figure S21: ESI-MS of synthesized analogue **8**. $M_{theoritical}$: 384.45; $(M_{theoritical} + H)^{+}$: 385.45; found 385.10.

S.2.1.5 Data of anthraquinone - GnRH conjugate con4

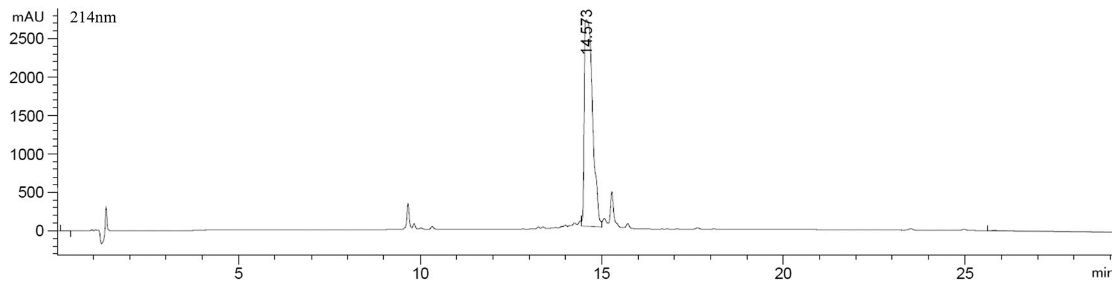

Figure S22: Analytical RP-HPLC chromatogram of synthesized conjugate **con4** at 214 nm.

RP-HPLC Conditions:

i) Column: Agilent ZORBAX Eclipse Plus C18 (3.5 µm, 100 x 4.6 mm), flow rate 1 ml/min,

ii) Solvents: H_2O (0.08 % TFA), ACN (0.08 % TFA),

iii) Gradient elution: from 10 % ACN to 100 % ACN in 30 min at RT,

iv) t_R: 14.573 min; Purity: 94 %.

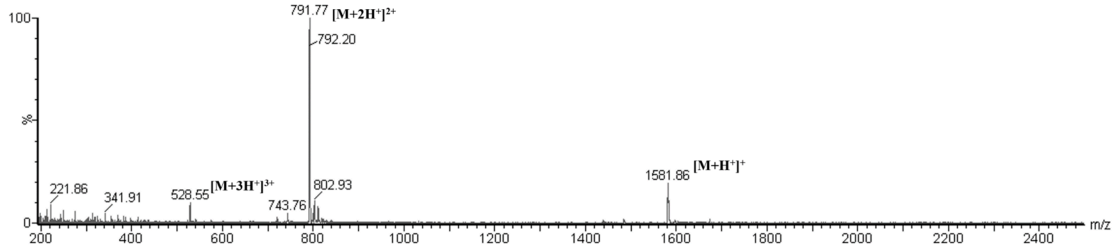

Figure S23: ESI-MS of synthesized conjugate **con4**. $M_{theoritical}$: 1581.83; $(M_{theoritical} + H)^+$: 1582.83; found 1581.86; $(M_{theoritical} + 2H^+)/2$: 791.92; found 791.77; $(M_{theoritical} + 3H^+)/3$: 528.28; found 528.55.

S.2.1.6 Data of anthraquinone - GnRH conjugate con5

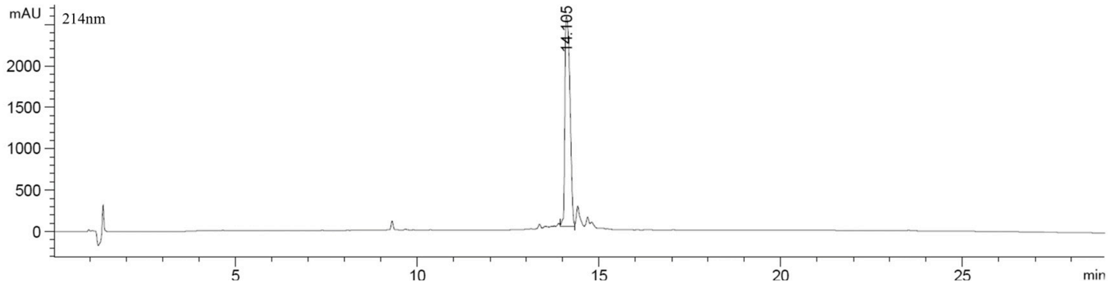

Figure S24: Analytical RP-HPLC chromatogram of synthesized conjugate **con5** at 214 nm.

RP-HPLC Conditions:

i) Column: Agilent ZORBAX Eclipse Plus C18 (3.5 µm, 100 x 4.6 mm), flow rate 1 ml/min,

ii) Solvents: H_2O (0.08 % TFA), ACN (0.08 % TFA),

iii) Gradient elution: from 10 % ACN to 100 % ACN in 30 min at RT,

iv) t_R: 14.105 min; Purity: 95 %.

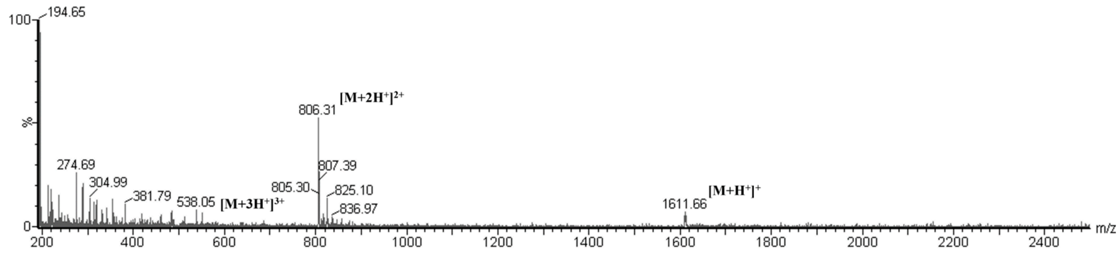

Figure S25: ESI-MS of synthesized conjugate **con5**. $M_{theoritical}$: 1610.83; $(M_{theoritical} + H)^+$: 1611.83; found 1611.66; $(M_{theoritical} + 2H^+)/2$: 806.42; found 806.31; $(M_{theoritical} + 3H^+)/3$: 537.94; found 538.05.

S.2.1.7 Data of anthraquinone - GnRH conjugate con6

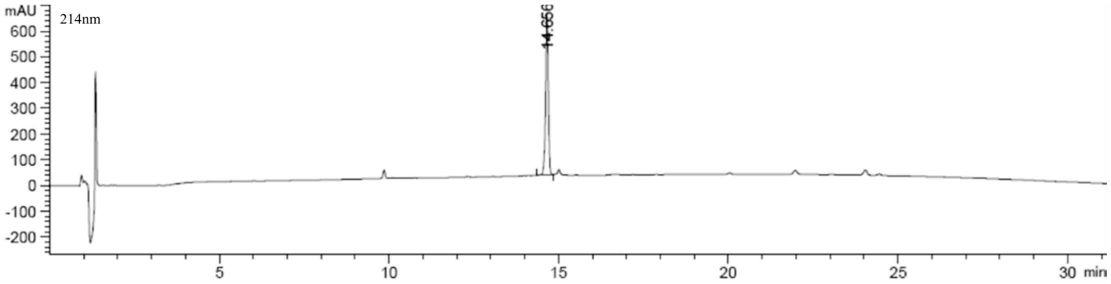

Figure S26: Analytical RP-HPLC chromatogram of synthesized conjugate **con6** at 214 nm.

RP-HPLC Conditions:

i) Column: Agilent ZORBAX Eclipse Plus C18 (3.5 µm, 100 x 4.6 mm), flow rate 1 ml/min,

ii) Solvents: H_2O (0.08 % TFA), ACN (0.08 % TFA),

iii) Gradient elution: from 10 % ACN to 100 % ACN in 30 min at RT,

iv) t_R: 14.656 min; Purity: 97 %.

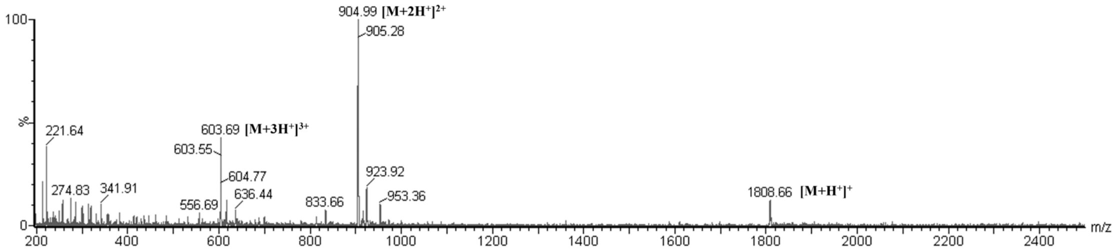

Figure S27: ESI-MS of synthesized conjugate **con6**. $M_{theoritical}$: 1808.15; $(M_{theoritical} + H)^+$: 1809.15; found 1808.66; $(M_{theoritical} + 2H^+)/2$: 905.08; found 904.99; $(M_{theoritical} + 3H^+)/3$: 603.72; found 603.69.

S.2.2 Synthesis of anthraquinone - GnRH conjugates (con1; con2; con8)

S.2.2.1 Synthesis of analogue 9

The synthesis of primary amine **11** was achieved through the Gabriel synthesis by using the commercially available potassium phthalimide and 1,4-dibromobutane in anhydrous DMF, as starting materials. First, to a 1,4-dibromobutane (0.14 ml, 1.19 mmol, 1.1 eq) solution in anhydrous DMF (12 ml), under argon at 0°C was added dropwise a solution of potassium phthalimide (0.20 g, 1.08 mmol, 1.0 eq) in DMF (100 ml) and after that was left at RT. The reaction mixture was monitored by TLC (40% EtOAc-Hex) until completion (~16 h). Solvents were removed under reduced pressure and purification by column chromatography on silica gel (20% EtOAc-Hex) provided analogue **9** as white solid (172.5 mg, 2.45 mmol, 56.82%).

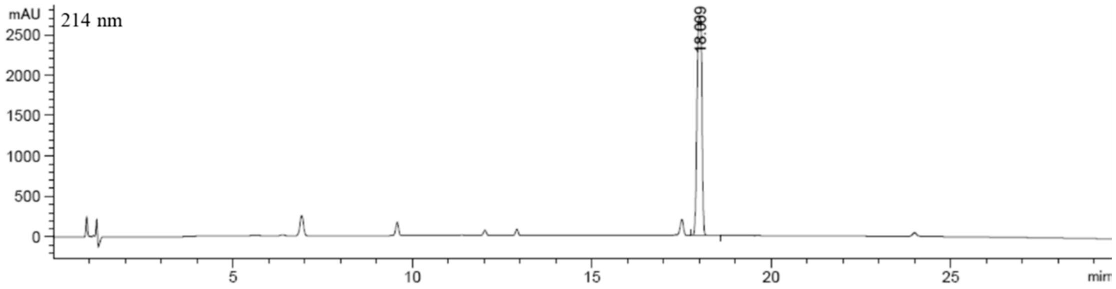

Figure S28: Analytical RP-HPLC chromatogram of synthesized analogue **9** at 214 nm.
RP-HPLC Conditions:
i) Column: Agilent ZORBAX Eclipse Plus C18 (3.5 μm, 100 x 4.6 mm), flow rate 1 ml/min,
ii) Solvents: H_2O (0.08 % TFA), ACN (0.08 % TFA),
iii) Gradient elution: from 10 % ACN to 100 % ACN in 30 min at RT,
iv) t_R: 18.009 min; Purity: 85%.

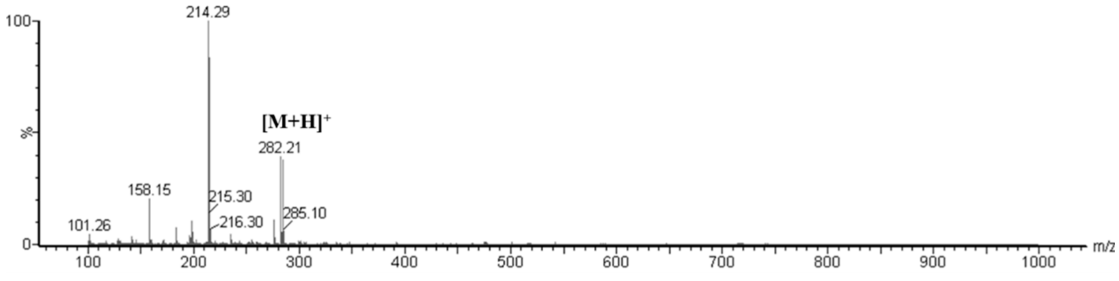

Figure S29: ESI-MS of synthesized analogue **9**. $M_{theoritical}$: 281.14; $(M_{theoritical} + H)^+$: 282.14; found 282.21.

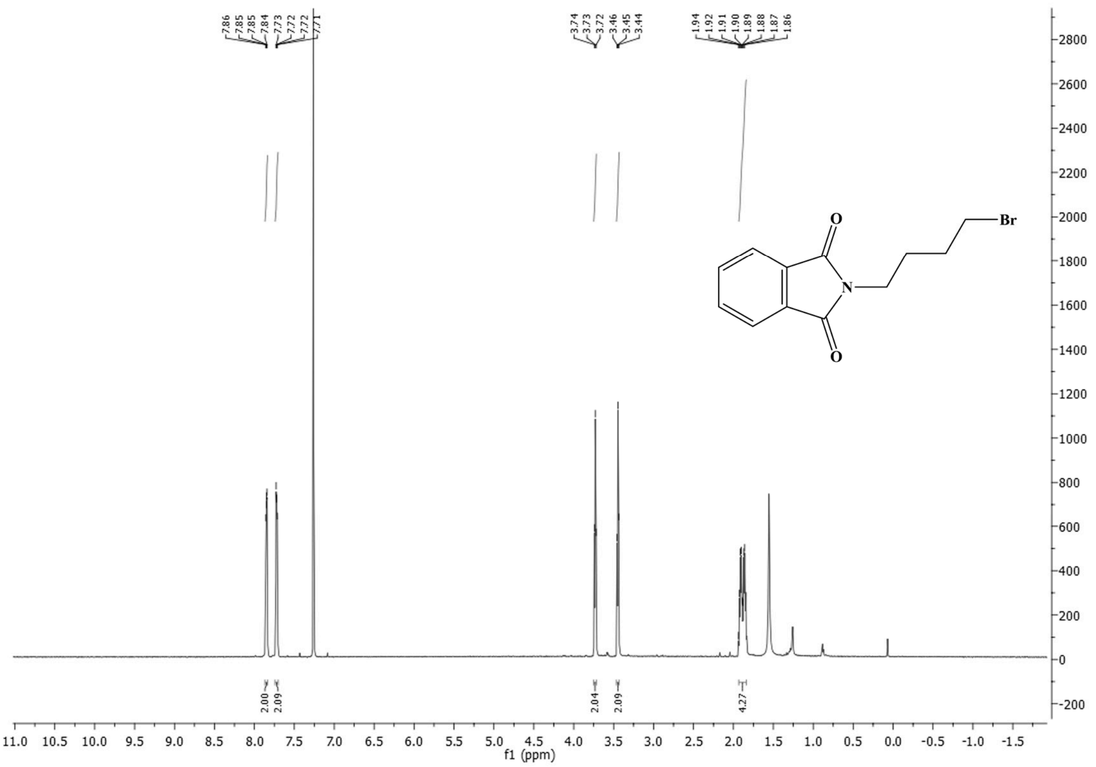

Figure S30: ^{1}H-NMR of synthesized analogue **9**. ^{1}H-NMR ($CDCl_3$, 600 MHz) δ 7.86 – 7.84 (m, 2H, CH^{Ar}), 7.73 – 7.71 (m, 2H, CH^{Ar}), 3.73 (t, J = 6.8 Hz, 2H, CH_2N), 3.45 (t, J = 6.4 Hz, 2H, CH_2Br), 1.94 – 1.86 (m, 4H, CH_2).

S.2.2.2 Synthesis of analogue 10

To a solution of triphenylmethanethiol (476.3 mg, 2.7 mmol, 1.1 eq) in anhydrous DMF (50 ml), under argon at 0°C was added NaH (147.1 mg, 6.13 mmol, 2.5 eq). After 30 min, was added dropwise a solution of analogue **9** (690.8 mg, 2.45 mmol, 1.0 eq) in anhydrous DMF (14 ml). The reaction mixture was monitored by TLC (40% EtOAc-Hex) until completion (~16 h). Then, it was diluted with the same volume of sat. NaCl solution, washed with EtOAc (3 × 30 ml) and the combined organic layer was dried over Na_2SO_4. Solvents were removed under reduced pressure to give **10** as pale-yellow solid (769 mg, 1.61 mmol, 65.72%), which was used in the next step without further purification.

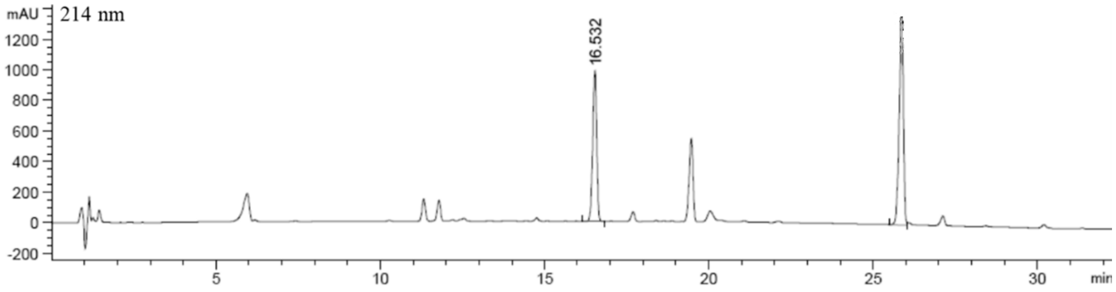

Figure S31: Analytical RP-HPLC chromatogram of synthesized analogue **10** at 214 nm.
RP-HPLC Conditions:
i) Column: Agilent ZORBAX Eclipse Plus C18 (3.5 μm, 100 x 4.6 mm), flow rate 1 ml/min,
ii) Solvents: H_2O (0.08 % TFA), ACN (0.08 % TFA),
iii) Gradient elution: from 30 % ACN to 100 % ACN in 30 min at RT,
iv) t_R: 16.532 min.

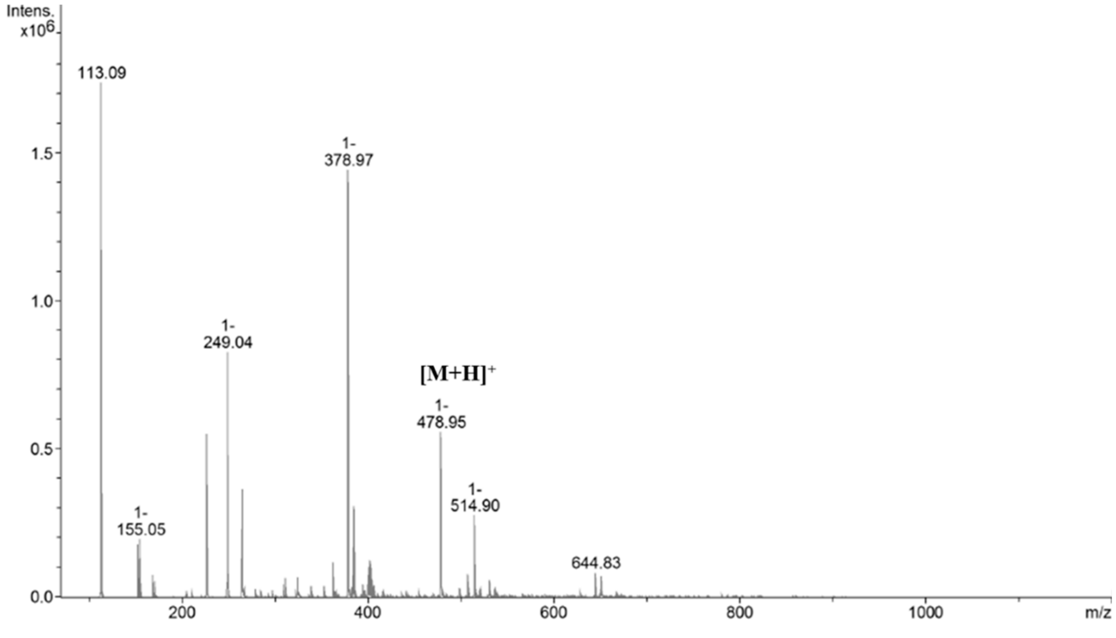

Figure S32: ESI-MS of synthesized analogue **10**. $M_{theoritical}$: 477.62; $(M_{theoritical} + H)^+$: 478.62; found 478.95.

S.2.2.3 Synthesis of analogue 11

Subsequent hydrazinolysis of **10** released the primary amine **11**. For this, analogue **10** (768.7 mg 1.61 mmol, 1.0 eq) was dissolved in *n*-BuOH-EtOH solution 5:1 (25 ml) and hydrazine (N_2H_4) was then added (0.36 ml, 11.27 mmol, 7.0 eq). The reaction mixture was stirred under reflux for 24 h and was monitored by TLC (15% MeOH-CH_2Cl_2). Purification by column chromatography on silica gel (15% MeOH-CH_2Cl_2) provided **11** as a white solid (550 mg, 1.58 mmol, 98.30%).

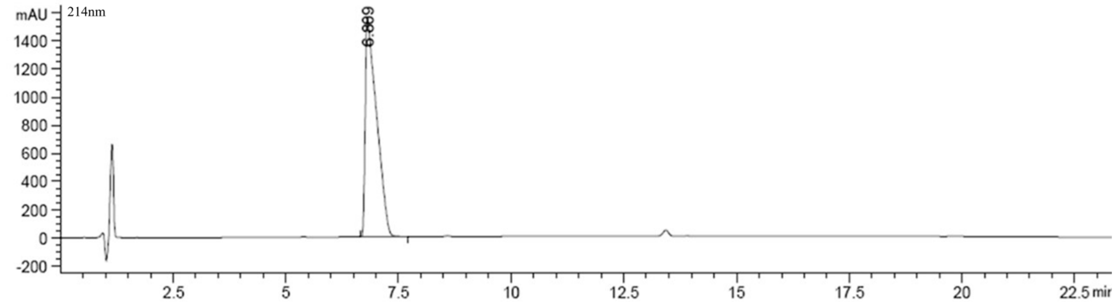

Figure S33: Analytical RP-HPLC chromatogram of synthesized analogue **11** at 214 nm.

RP-HPLC Conditions:

i) Column: Agilent ZORBAX Eclipse Plus C18 (3.5 µm, 100 x 4.6 mm), flow rate 1 ml/min,

ii) Solvents: H_2O (0.08 % TFA), ACN (0.08 % TFA),

iii) Gradient elution: from 40 % ACN to 100 % ACN in 30 min at RT,

iv) t_R: 6.809 min; Purity: 98%.

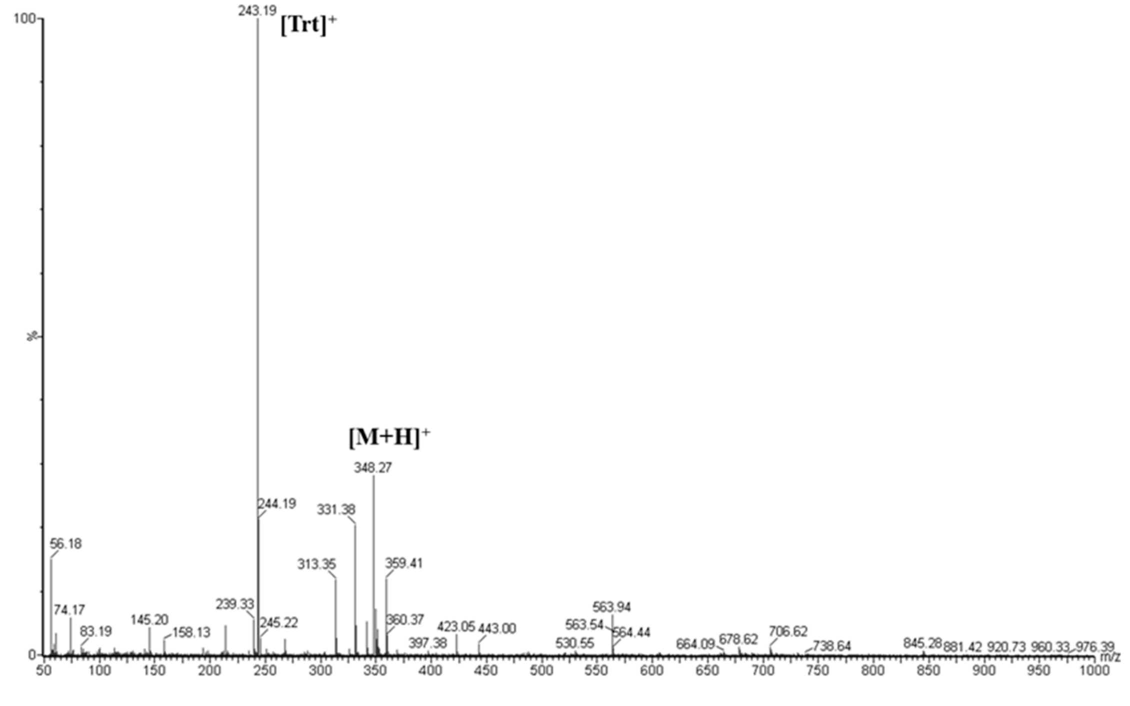

Figure S34: ESI-MS of synthesized analogue **11**. $M_{theoritical}$: 347.52; $(M_{theoritical} + H)^+$: 348.52; found 348.27; $(Trt)^+$: 243.12; found 243.19.

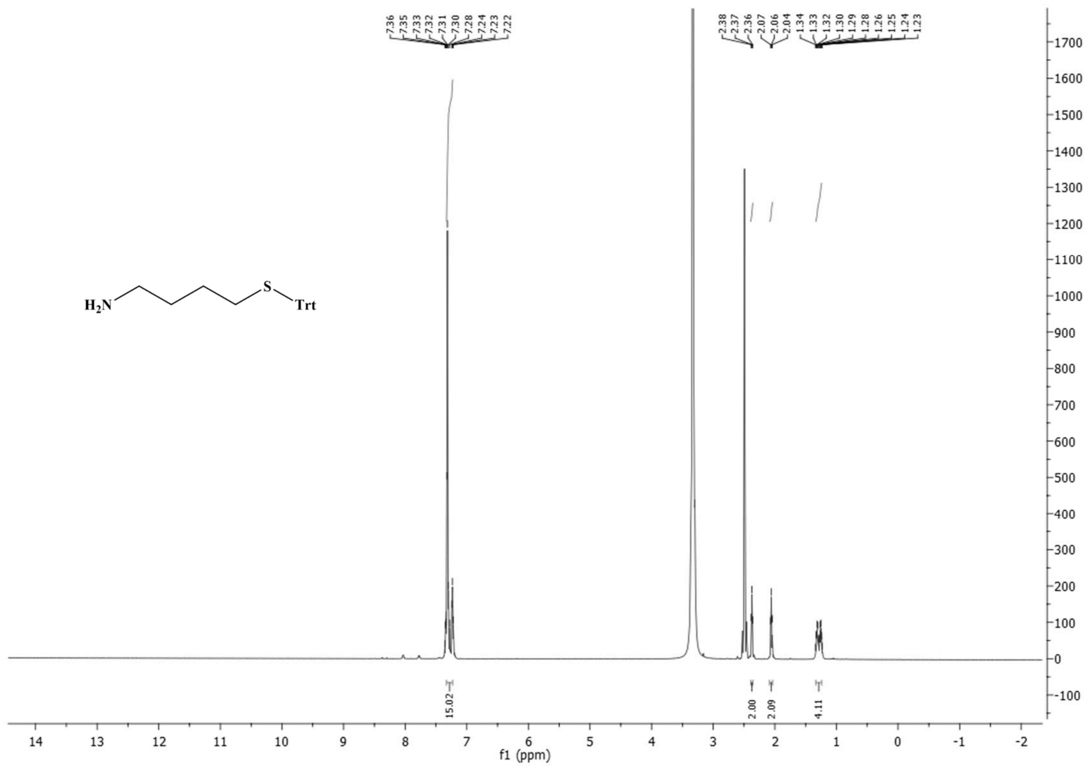

Figure S35: ^{1}H-NMR of synthesized analogue **11**. ^{1}H-NMR (d^6-DMSO, 600 MHz) δ 7.36 – 7.22 (m, 15H, CH^{Ar}), 2.37 (t, J = 6.8 Hz, 2H, CH_2NH_2), 2.06 (t, J = 7.2 Hz, 2H, CH_2STrt), 1.34 – 1.23 (m, 4H, CH_2).

S.2.2.4 Synthesis of anthraquinone analogue 12

Then, analogue **11** reacted with the commercially available leucoquinizarin, followed by air oxidation (aromatization) of the anthraquinone ring to obtain analogue **12**. Thus, to a solution of leucoquinizarin (85.3 mg, 0.352 mmol, 2.0 eq) in deoxygenated EtOH (5 ml) was added dropwise a solution of analogue **11** (60.9 mg, 0.176 mmol, 1.0 eq) in deoxygenated EtOH (2.5 ml) over a period of 1 h. The reaction mixture was stirred under reflux conditions for 3 h and after at 50°C for 18 h. Then, it was left under air bubbling oxidation for 3 h. The reaction mixture was monitored by TLC (50% Hex- CH_2Cl_2 and 10% MeOH-CH_2Cl_2) until completion. The solvent was evaporated under reduced pressure and purification by column chromatography on silica gel (50% Hex- CH_2Cl_2) afforded analogue **12** as a purple solid (19 mg, 0.033 mmol, 18.95%).

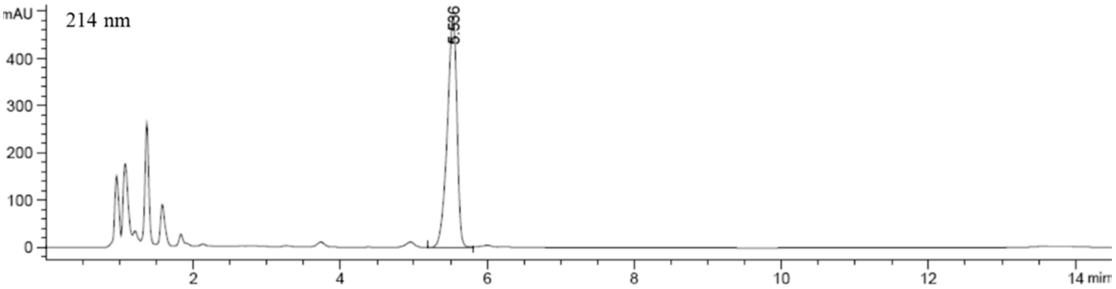

Figure S36: Analytical RP-HPLC chromatogram of synthesized analogue **12** at 214 nm.

RP-HPLC Conditions:

i) Column: Agilent ZORBAX Eclipse Plus C18 (3.5 µm, 100 x 4.6 mm), flow rate 1 ml/min,

ii) Solvents: H_2O (0.08 % TFA), ACN (0.08 % TFA),

iii) Gradient elution: from 95% ACN to 100 % ACN in 15 min at RT,

iv) t_R: 5.536 min; Purity: 80%.

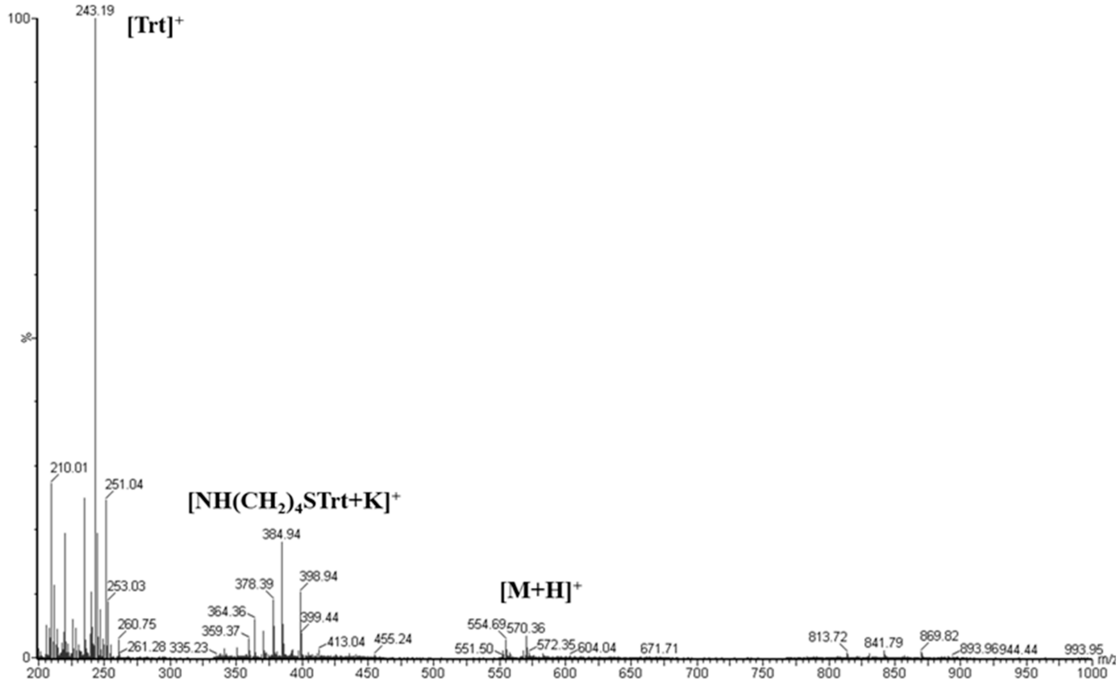

Figure S37: ESI-MS of synthesized analogue **12** $M_{theoritical}$: 569.71; $(M_{theoritical} + H)^+$: 570.71; found 570.36; $(NH(CH_2)_4STrt + K)^+$: 385.13; found 384.94; $(Trt)^+$: 243.12; found 243.19.

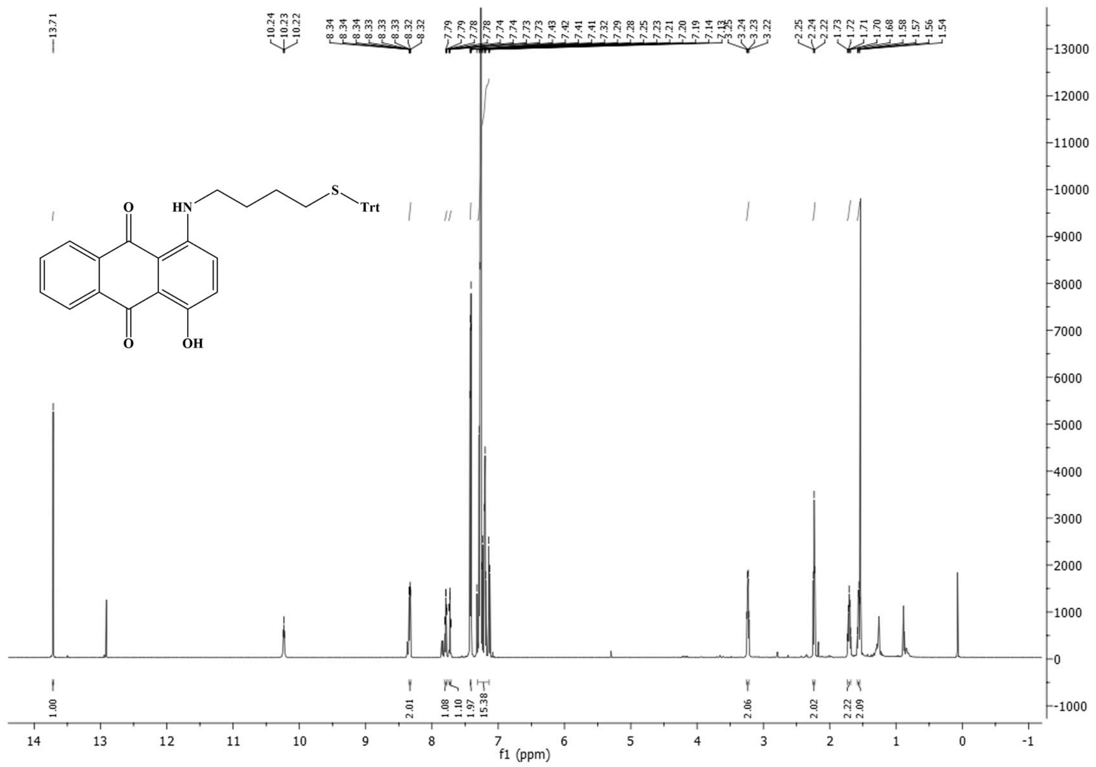

Figure S38: ^{1}H-NMR of synthesized analogue **12**. ^{1}H-NMR ($CDCl_3$, 600 MHz) δ 13.71 (s, 1H, OH), 10.23 (t, J = 5.0 Hz, 1H, NH), 8.34 – 8.32 (m, 2H, CH^{Ar}), 7.79 – 7.78 (m, 1H, CH^{Ar}), 7.73 (td, J = 7.2, 1.2 Hz, 1H, CH^{Ar}), 7.43 – 7.41 (m, 2H, CH^{Ar}), 7.32 – 7.13 (m, 15H, CH^{Ar}), 3.25 – 3.22 (m, 2H, CH_2NH), 2.24 (t, J = 7.2 Hz, 2H, CH_2STrt), 1.73 – 1.68 (m, 2H, CH_2), 1.58 – 1.54 (m, 2H, CH_2).

S.2.2.5 Synthesis of anthraquinone analogue 13

Eventually, the removal of the trityl-protecting group of analogue **12** (30 mg) was achieved by treatment with a mixture of TFA/ CH_2Cl_2/ TES 94:3:3 (2 ml) at RT for 3 h. Solvents were removed under reduced pressure and afforded purple solid **13** as ammonium trifluoroacetate salt (16.5 mg, 0.050 mmol, 95.71%), which was used in the next step without further purification.

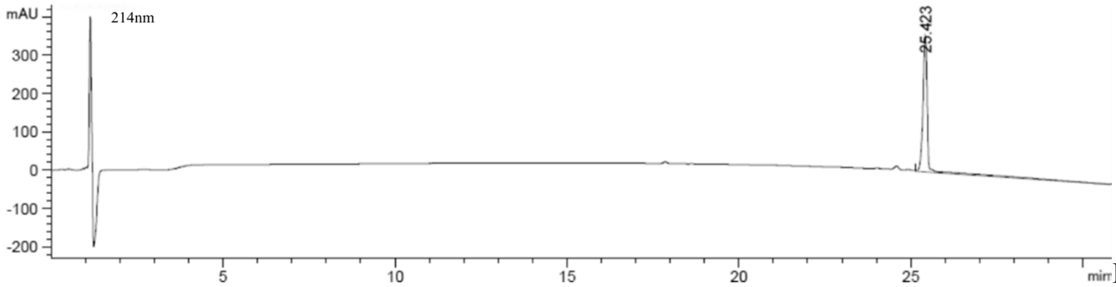

Figure S39: Analytical RP-HPLC chromatogram of synthesized analogue **13** at 214 nm.

RP-HPLC Conditions:

i) Column: Agilent ZORBAX Eclipse Plus C18 (3.5 µm, 100 x 4.6 mm), flow rate 1 ml/min,

ii) Solvents: H_2O (0.08 % TFA), ACN (0.08 % TFA),

iii) Gradient elution: from 10% ACN to 100 % ACN in 30 min at RT,

iv) t_R: 25.423 min.

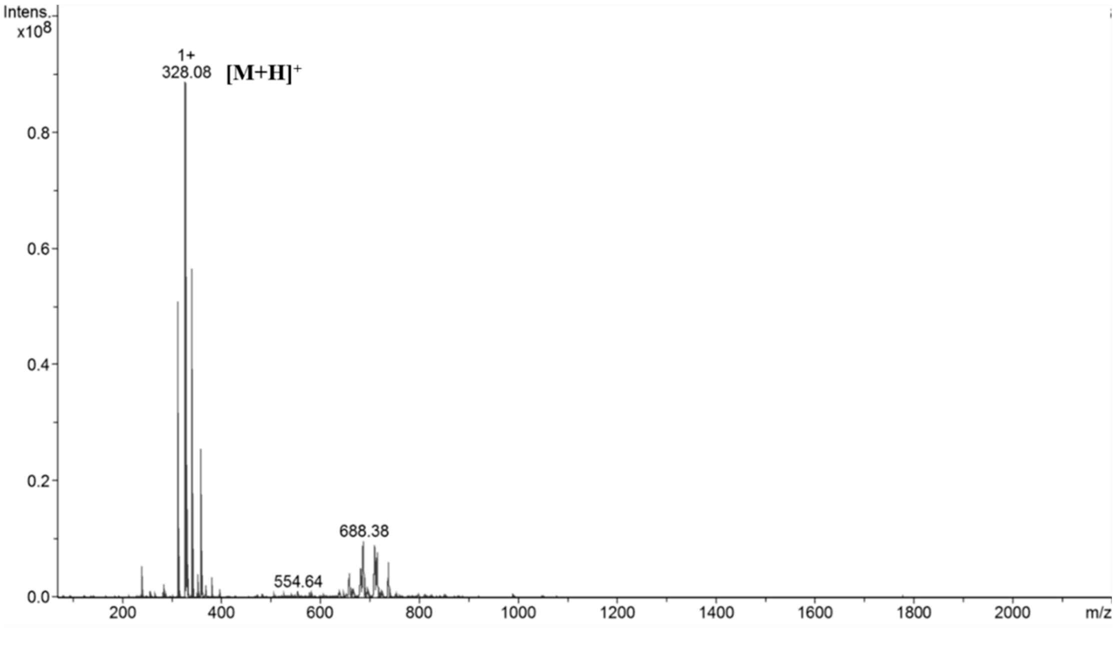

Figure S40: ESI-MS of synthesized analogue **13**. $M_{theoritical}$: 327.40; $(M_{theoritical} + H)^+$: 328.40; found 328.08.

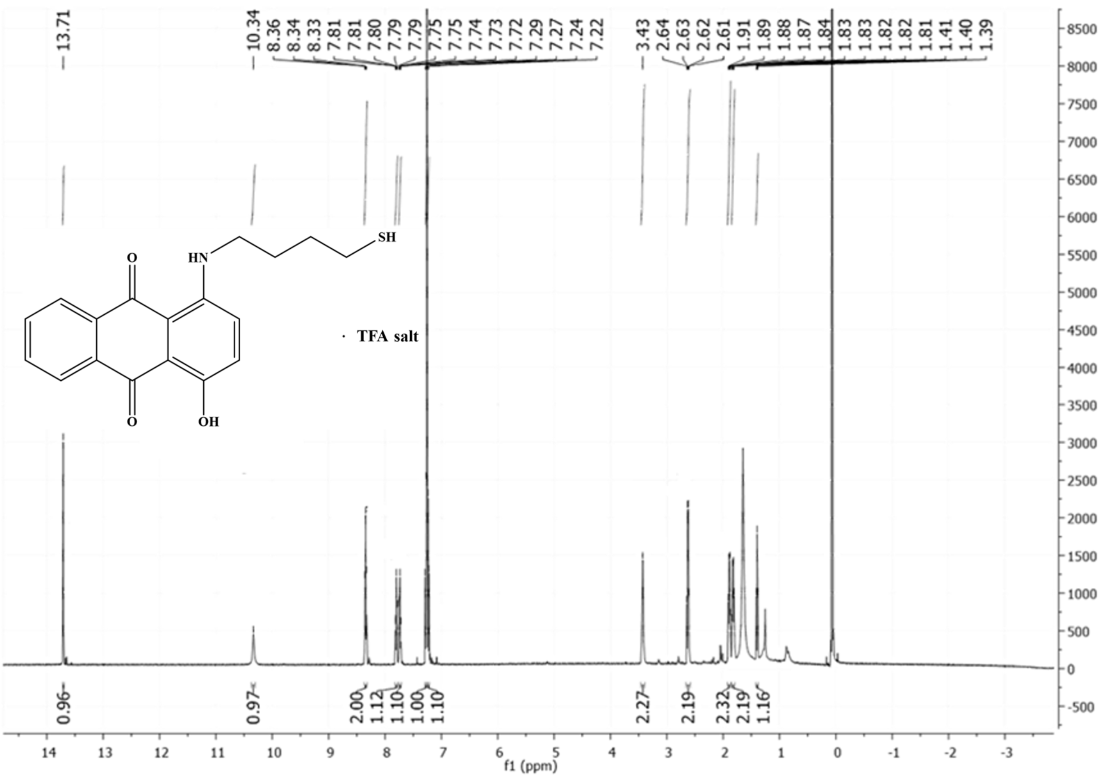

Figure S41: ^{1}H-NMR of synthesized analogue **13**: ^{1}H-NMR ($CDCl_3$, 600 MHz) δ 13.71 (s, 1H, OH), 10.34 (s, 1H, NH), 8.34 (t, J = 8.7 Hz, 2H, CH^{Ar}), 7.82 – 7.78 (m, 1H, CH^{Ar}), 7.76 – 7.71 (m, 1H, CH^{Ar}), 7.28 (d, J = 9.5 Hz, 1H, CH^{Ar}), 7.23 (d, J = 9.5 Hz, 1H, CH^{Ar}), 3.43 (m, 2H, CH_2NH), 2.64 – 2.61 (m, 2H, CH_2SH), 1.91 – 1.87 (m, 2H, CH_2), 1.84 – 1.81 (m, 2H, CH_2), 1.40 (t, J = 7.9 Hz, 1H, SH).

S.2.2.6 Data of anthraquinone - GnRH conjugate con1

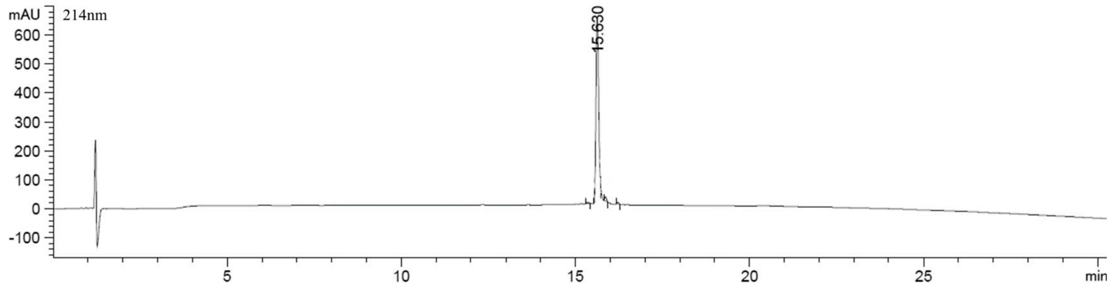

Figure S42: Analytical RP-HPLC chromatogram of synthesized conjugate **con1** at 214 nm.

RP-HPLC Conditions:

i) Column: Agilent ZORBAX Eclipse Plus C18 (3.5 μm, 100 x 4.6 mm), flow rate 1 ml/min,

ii) Solvents: H_2O (0.08 % TFA), ACN (0.08 % TFA),

iii) Gradient elution: from 10% ACN to 100 % ACN in 30 min at RT,

iv) t_R: 15.630 min; Purity: 97%.

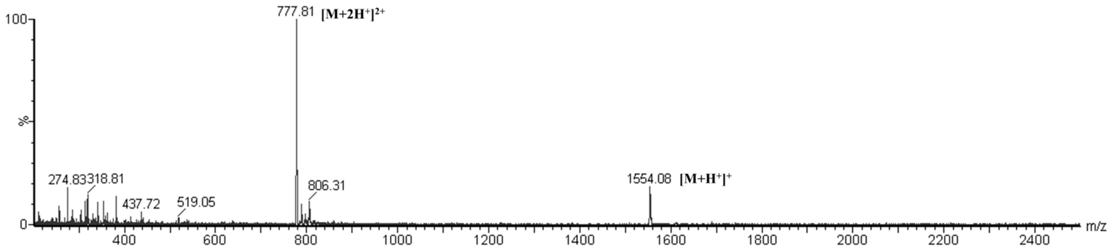

Figure S43: ESI-MS of synthesized conjugate **con1**. $M_{theoritical}$: 1553.64; $(M_{theoritical} + H)^+$: 1554.64; found 1554.08; $(M_{theoritical} + 2H^+)/2$: 777.32; found 777.81.

S.2.2.7 Data of anthraquinone - GnRH conjugate con2

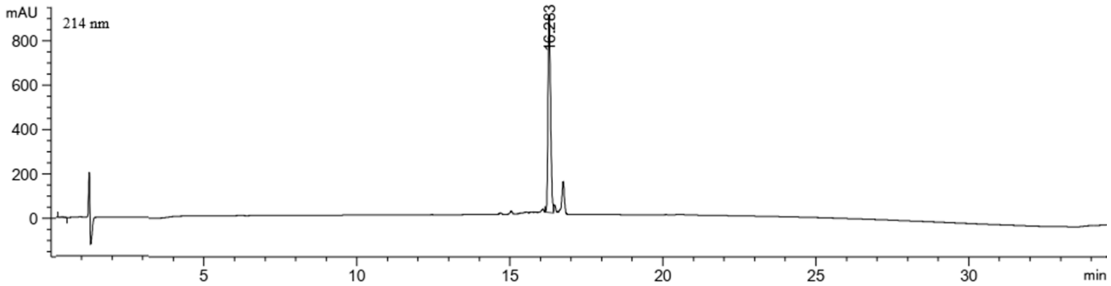

Figure S44: Analytical RP-HPLC chromatogram of synthesized conjugate **con2** at 214 nm.

RP-HPLC Conditions:

i) Column: Agilent ZORBAX Eclipse Plus C18 (3.5 µm, 100 x 4.6 mm), flow rate 1 ml/min,

ii) Solvents: H_2O (0.08 % TFA), ACN (0.08 % TFA),

iii) Gradient elution: from 10% ACN to 100 % ACN in 30 min at RT,

iv) t_R: 16.283 min; Purity: 95%.

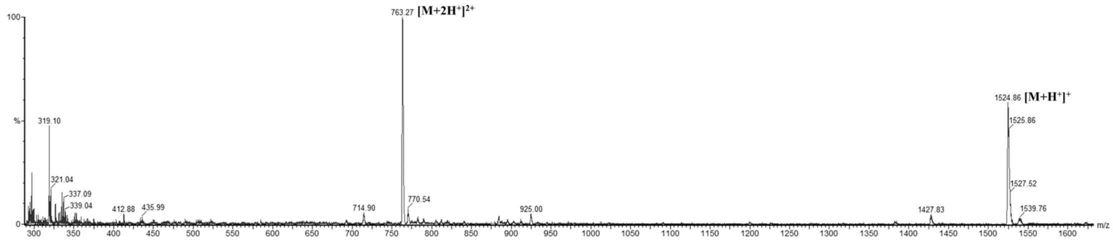

Figure S45: ESI-MS of synthesized conjugate **con2**. $M_{theoritical}$: 1524.78; $(M_{theoritical} + H)^{+}$: 1525.78; found 1524.86; $(M_{theoritical} + 2H^{+})/2$: 763.39; found 763.27.

S.2.2.8 Data of anthraquinone - GnRH conjugate con8

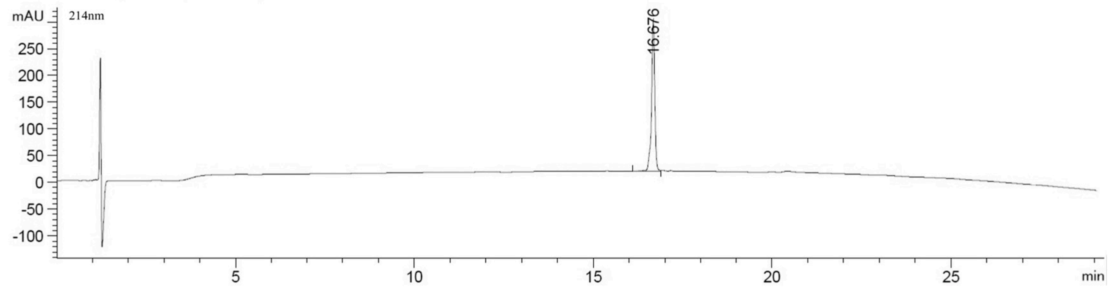

Figure S46: Analytical RP-HPLC chromatogram of synthesized conjugate **con8** at 214 nm.

RP-HPLC Conditions:

i) Column: Agilent ZORBAX Eclipse Plus C18 (3.5 µm, 100 x 4.6 mm), flow rate 1 ml/min,

ii) Solvents: H_2O (0.08 % TFA), ACN (0.08 % TFA),

iii) Gradient elution: from 10% ACN to 100 % ACN in 30 min at RT,

iv) t_R: 16.676 min; Purity: 99%.

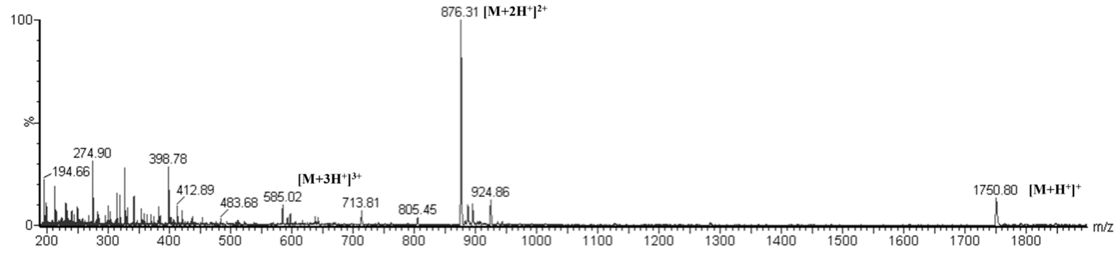

Figure S47: ESI-MS of synthesized conjugate **con8**. $M_{theoritical}$: 1749.82; ($M_{theoritical}$ + H)$^+$: 1750.82; found 1750.80; ($M_{theoritical}$ + 2H$^+$)/2: 875.91; found 876.31; ($M_{theoritical}$ + 3H$^+$)/3: 584.27; found 585.02.

S.2.3 Synthesis of mitoxantrone - GnRH conjugate (con7; con3)

S.2.3.1 Synthesis of mitoxantrone - GnRH conjugate (con7)

S.2.3.1.1 Synthesis of mitoxantrone analogue 14

To a solution of commercially available mitoxantrone dihydrochloride (100.0 mg, 0.193 mmol, 1.0 eq) in anhydrous MeOH (200 ml), under argon at 0°C was added TEA (8 ml). After 1 h, was added dropwise a solution of di-*tert*-butyl dicarbonate (0.53 ml, 2.32 mmol, 8.0 eq) in anhydrous THF (8ml) for protection of aliphatic amines to take place. The reaction mixture was monitored by analytical RP-HPLC until completion (~ 2 h). Solvents were removed under reduced pressure and the residue was dissolved in EtOAc (60 ml), washed with sat. K_2CO_3 solution (3 × 50 ml) and the combined organic layer was dried over Na_2SO_4. Solvents were removed under reduced pressure and purification by preparative HPLC (gradient separation 40 to 100% ACN in 43 min, flow rate 12 ml/min) provided analogue **14** as blue solid (33 mg, 0,051 mmol, 26.52%).

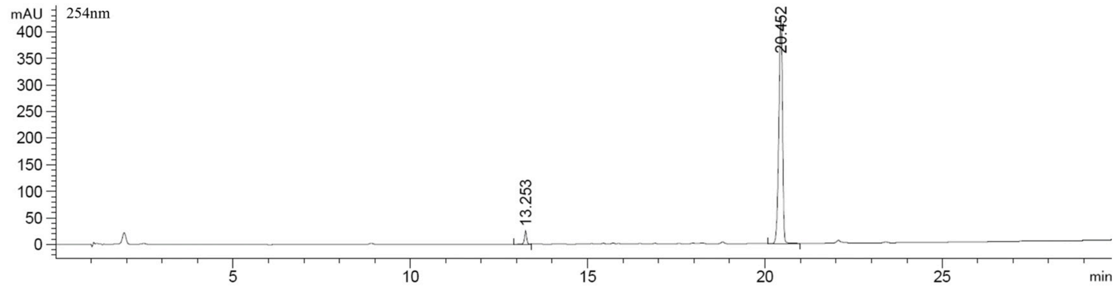

Figure S48: Analytical RP-HPLC chromatogram of synthesized analogue **14** at 254 nm.

RP-HPLC Conditions:

i) Column: Agilent ZORBAX Eclipse Plus C18 (3.5 μm, 100 x 4.6 mm), flow rate 1 ml/min,

ii) Solvents: H_2O (0.08 % TFA), ACN (0.08 % TFA),

iii) Gradient elution: from 10% ACN to 100 % ACN in 30 min at RT,

iv) t_R: 20.452 min; Purity: 97%.

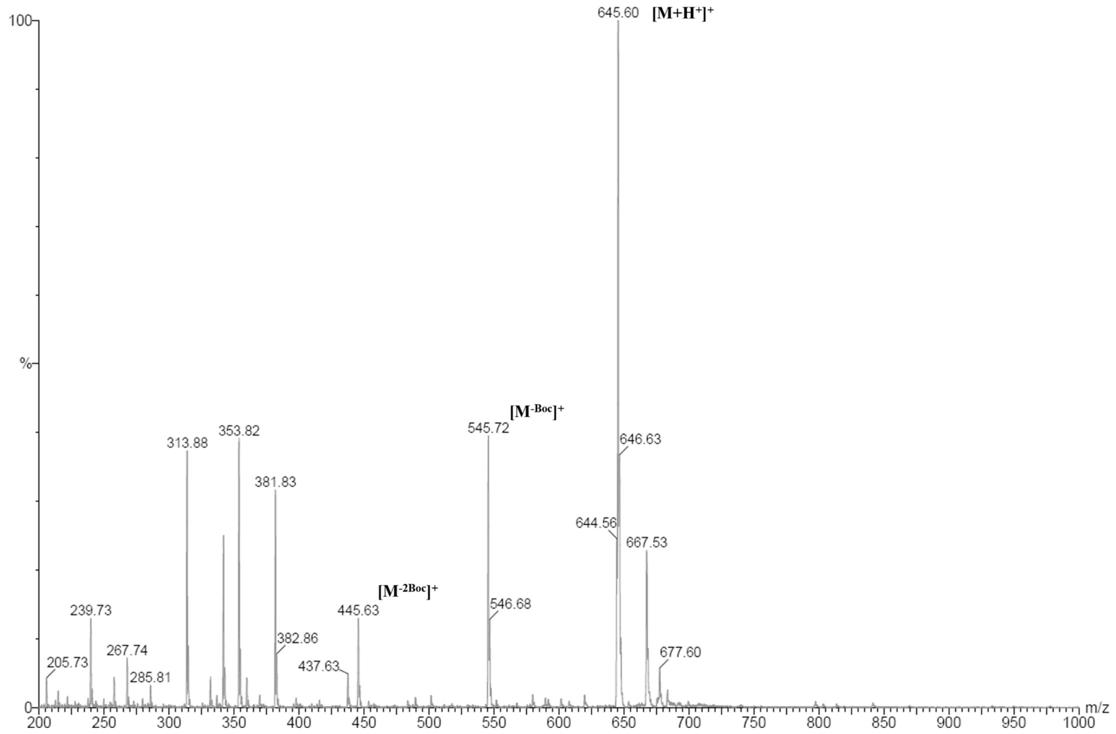

Figure S49: ESI-MS of synthesized analogue **14**. $M_{theoritical}$: 644.72; $(M_{theoritical} + H)^+$: 645.72; found 645.60; $(M_{theoritical} – Boc)^+$: 543.60; found 545.72; $(M_{theoritical} – 2Boc)^+$: 442.48; found 445.63.

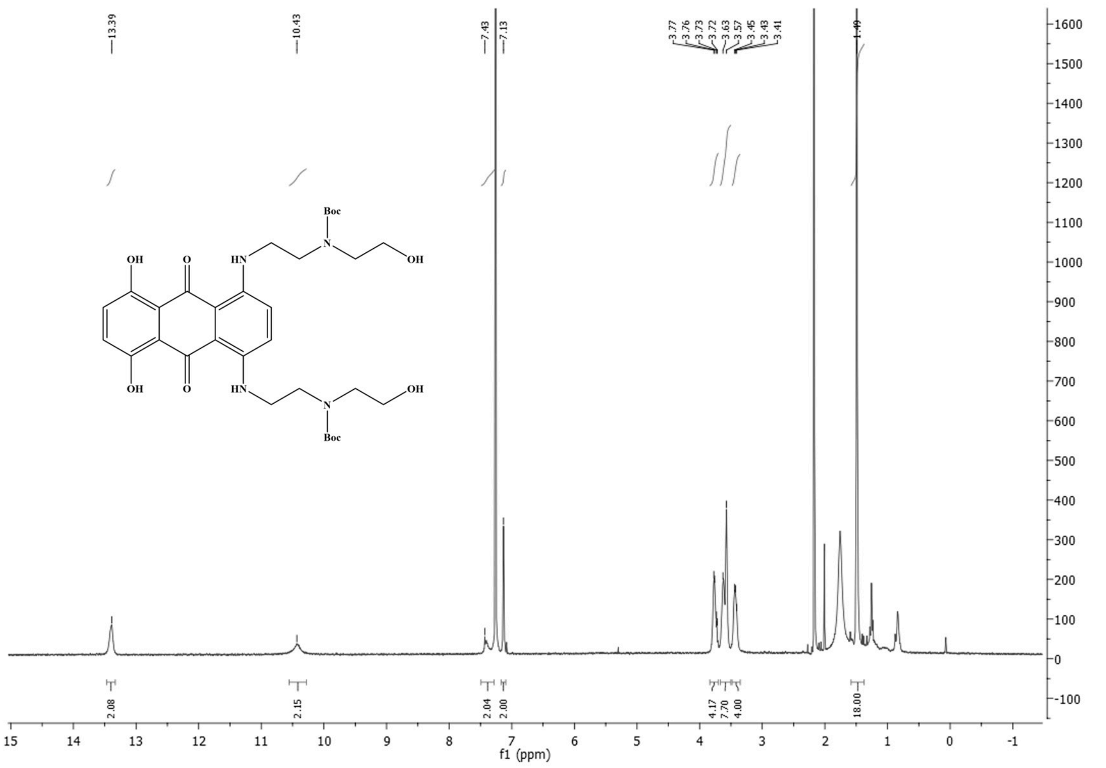

Figure S50: ^{1}H-NMR of synthesized analogue **14**. ^{1}H-NMR ($CDCl_3$, 600 MHz) δ 13.39 (s, 2H, OH), 10.43 (br s, 2H, NH), 7.43 (s, 2H, CH^{Ar}), 7.13 (s, 2H, CH^{Ar}), 3.77-3.72 (m, 4H, CH_2N), 3.63 – 3.57 (m, 8H, CH_2OH, CH_2N), 3.45 – 3.41 (m, 4H, CH_2), 1.49 (s, 18H, Boc).

S.2.3.1.2 Synthesis of mitoxantrone analogue 15

Subsequently, the formation of an ester bond in one of the side chains of analogue **14** took place via a modified Steglich esterification. Thus, in a round bottom flask **A** were dissolved *S*-trityl-mercaptoacetic acid (22.7 mg, 0.067 mmol, 1.2 eq), DMAP (20.7 mg, 0.17 mmol, 3.0 eq), HOBt (23.1 mg, 0.17 mmol, 3.0 eq) and DIC (17.6 μL, 0.11 mmol, 2.0 eq) in CH_2Cl_2 (3 ml) and the reaction mixture was left stirred at 0°C for 1 h. In parallel, analogue **14** (36.5 mg, 0.057 mmol, 1.0 eq) was dissolved in round bottom flask **B** in CH_2Cl_2 (200 ml) and was left stirred at 0°C. The solution in **A** was additionally diluted with CH_2Cl_2 (5 ml) and was added dropwise in **B** over 30 min. The reaction mixture was stirred at 0°C for 24 h and was monitored by analytical RP-HPLC. The solvent was evaporated under reduced pressure to provide analogue **15** as a blue solid (110 mg, 0.11mmol, 66.69%), which was used in the next step without further purification.

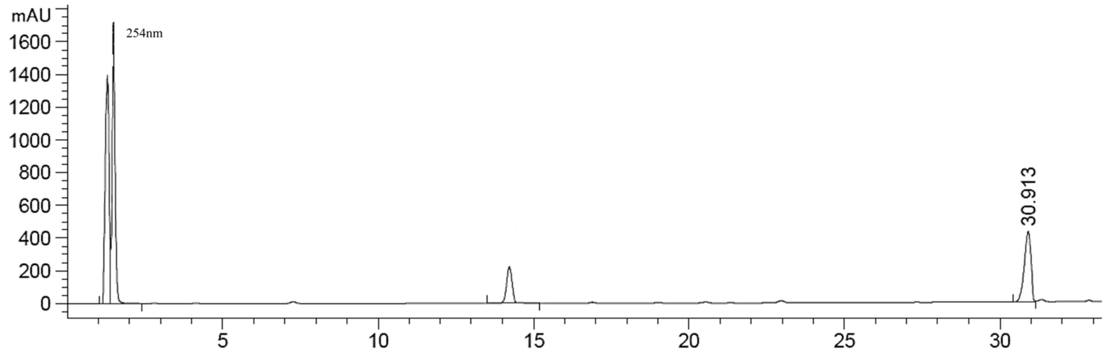

Figure S51: Analytical RP-HPLC chromatogram of synthesized analogue **15** at 254 nm.
RP-HPLC Conditions:
i) Column: Agilent ZORBAX Eclipse Plus C18 (3.5 μm, 100 x 4.6 mm), flow rate 1 ml/min,
ii) Solvents: H_2O (0.08 % TFA), ACN (0.08 % TFA),
iii) Gradient elution: from 40% ACN to 100 % ACN in 30 min at RT,
iv) t_R: 30.913 min.

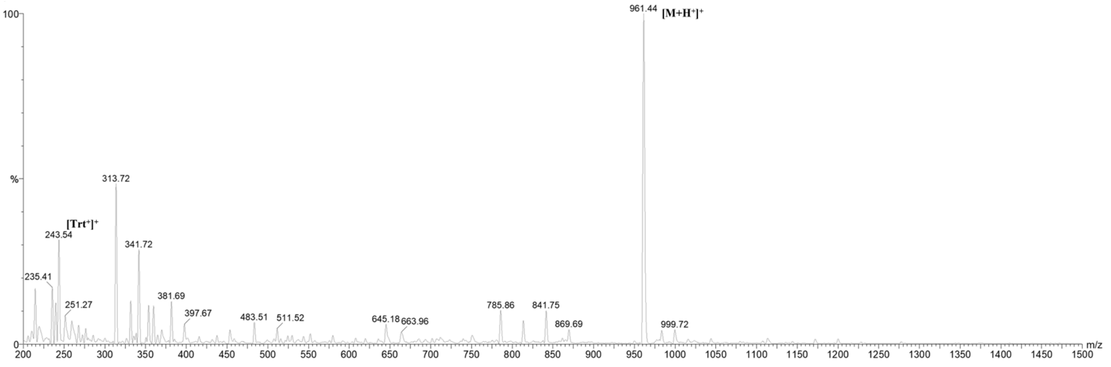

Figure S52: ESI-MS of synthesized analogue **15**. $M_{theoritical}$: 960.14; $(M_{theoritical} + H)^+$: 961.14; found 961.44; $(Trt)^+$: 243.12; found 243.54.

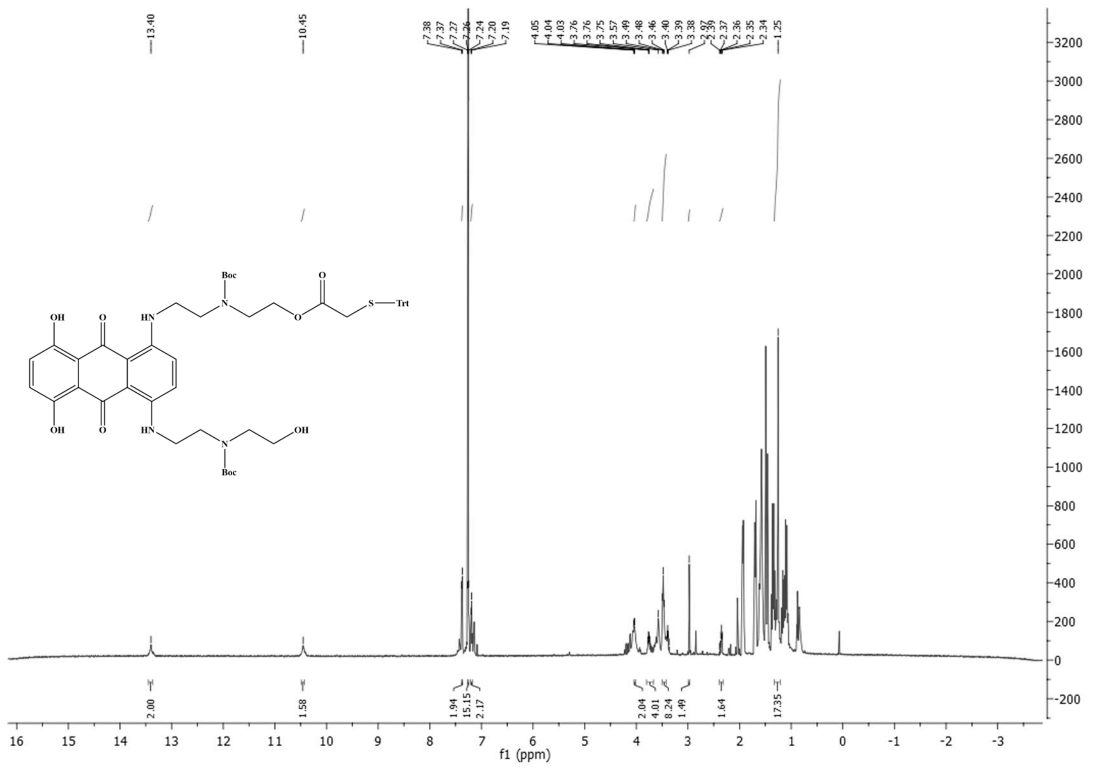

Figure S53: ^{1}H-NMR of synthesized analogue **15**. ^{1}H-NMR ($CDCl_3$, 600 MHz) δ 13.40 (s, 2H, OH), 10.45 (s, 2H, NH), 7.38 (d, *J* = 7.6 Hz, 2H, CH^{Ar}), 7.27 – 7.24 (m, 15H, Trt), 7.20 (d, *J* = 7.3 Hz, 2H, CH^{Ar}), 4.05 – 4.03 (m, 2H, CH_2O), 3.78 – 3.74 (m, 4H, CH_2N), 3.57 – 3.38 (m, 8H, CH_2OH, CH_2N), 2.97 (s, 2H, CH_2S), 2.39-2.34 (m, 2H, CH_2N), 1.25 (s, 18H, Boc).

S.2.3.1.3 Synthesis of mitoxantrone analogue 16

The final removal of trityl- and Boc-protecting groups from analogue **15** (110 mg) was achieved by treatment with a mixture of TFA:CH_2Cl_2 1:1 (6 ml) and 3 drops of TES at RT for 1.5 h. Solvents were removed under reduced pressure and afforded blue solid, which was washed with MeOH (3 × 4 ml). Methanol was then removed under reduced pressure and the crude was purified by preparative HPLC (gradient separation 10 to 70% ACN in 43 min, flow rate 12 ml/min) to provide analogue **16** as blue solid (14 mg, 0.027 mmol, 23.57%).

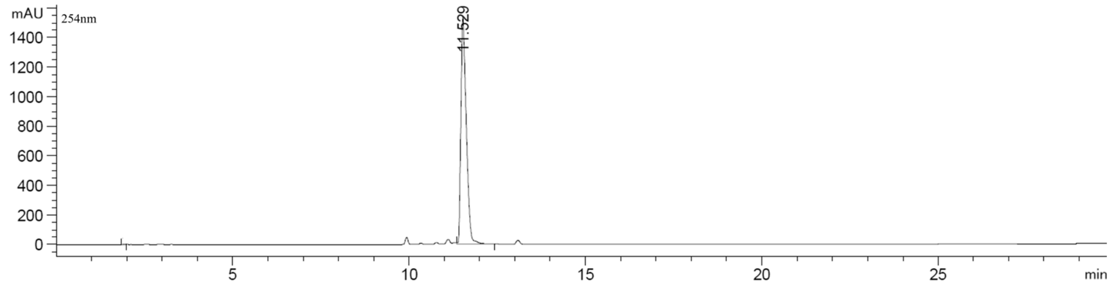

Figure S54: Analytical RP-HPLC chromatogram of synthesized analogue **16** at 254 nm.
RP-HPLC Conditions:
i) Column: Agilent ZORBAX Eclipse Plus C18 (3.5 µm, 100 x 4.6 mm), flow rate 1 ml/min,
ii) Solvents: H_2O (0.08 % TFA), ACN (0.08 % TFA),
iii) Gradient elution: from 10% ACN to 100 % ACN in 30 min at RT,
iv) t_R: 11.529 min; Purity: 96%.

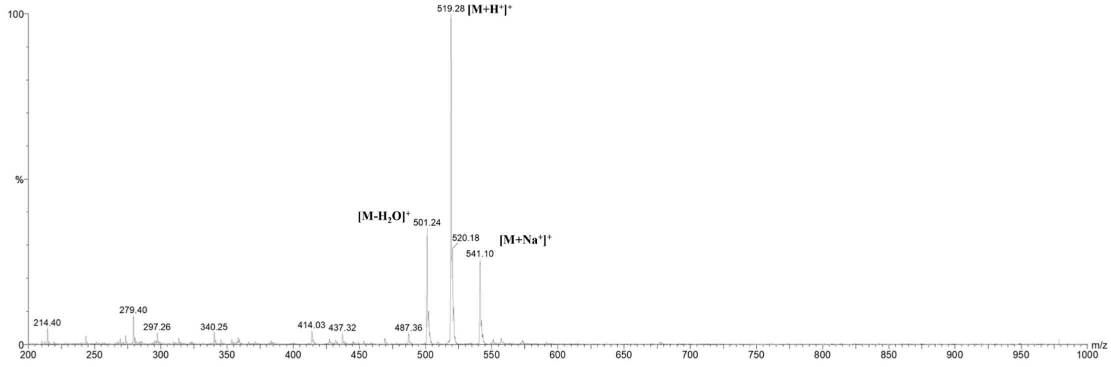

Figure S55: ESI-MS of synthesized analogue **16**. $M_{theoritical}$: 518.59; $(M_{theoritical} + H)^+$: 519.59; found 519.28; $(M_{theoritical} + Na^+)^+$: 541.59; found 541.10; $(M_{theoritical} - H_2O)^+$: 500.59; found 501.24.

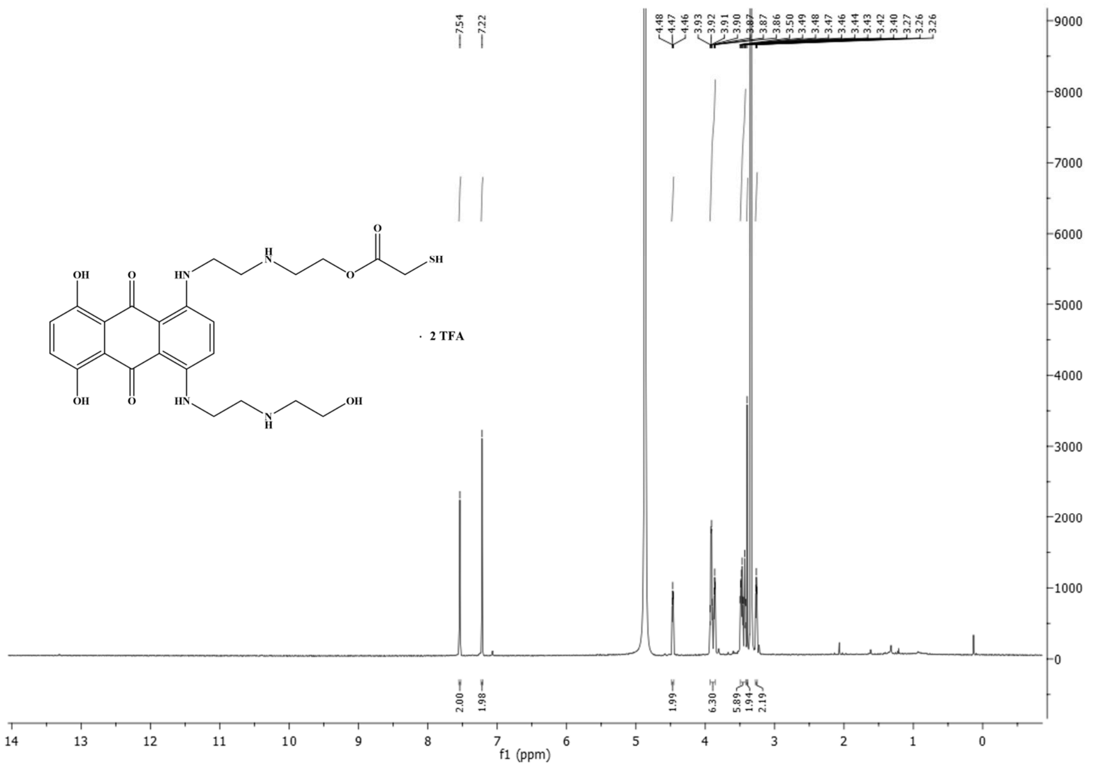

Figure S56: ^{1}H-NMR of synthesized analogue **16**. ^{1}H-NMR (d^4-MeOD, 600 MHz) δ 7.54 (s, 2H, CH^{Ar}), 7.22 (s, 2H, CH^{Ar}), 4.48 – 4.46 (m, 2H, CH_2O), 3.93 – 3.86 (m, 6H, CH_2N, CH_2OH), 3.50 – 3.42 (m, 6H, CH_2N), 3.40 (s, 2H, CH_2S), 3.27 – 3.26 (m, 2H, CH_2N).

S.2.3.1.4 Data of mitoxantrone- GnRH conjugate con7

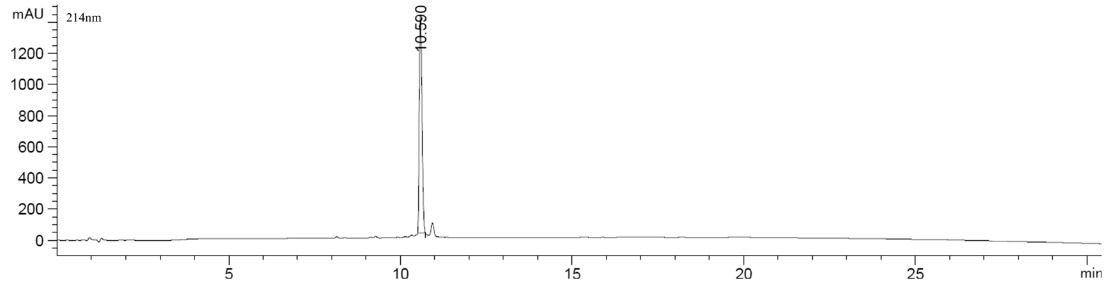

Figure S57: Analytical RP-HPLC chromatogram of synthesized conjugate **con7** at 214 nm.

RP-HPLC Conditions:

i) Column: Agilent ZORBAX Eclipse Plus C18 (3.5 µm, 100 x 4.6 mm), flow rate 1 ml/min,

ii) Solvents: H_2O (0.08 % TFA), ACN (0.08 % TFA),

iii) Gradient elution: from 10% ACN to 100 % ACN in 30 min at RT,

iv) t_R: 10.590 min; Purity: 97.4%.

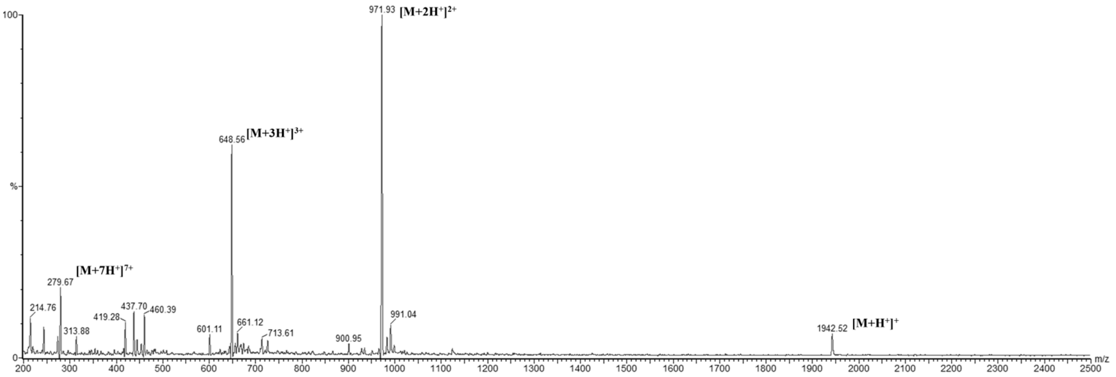

Figure S58: ESI-MS of synthesized conjugate **con7**. $M_{theoritical}$: 1942.29; $(M_{theoritical} + H)^+$: 1943.29; found 1942.52 $(M_{theoritical} + 2H^+)/2$: 972.15; found 971.93 $(M_{theoritical} + 3H^+)/3$: 648.43; found 648.56; $(M_{theoritical} + 7H^+)/7$: 278.47; found 279.67.

S.2.3.2 Synthesis of mitoxantrone - GnRH conjugate (con3)

S.2.3.2.1 Synthesis of mitoxantrone analogue 17

In a round bottom flask **A** were dissolved 3-(2-pyridyldithio)-propanoic acid (15.5 mg, 0.072 mmol, 1.2 eq), DMAP (22.0 mg, 0.18 mmol, 3.0 eq), HOBt (24.3 mg, 0.18 mmol, 3.0 eq) and DIC (18.7 µL, 0.12 mmol, 2.0 eq) in CH_2Cl_2 (3 ml) and was left stirred at 0°C for 1 h. Analogue **14** (38.7 mg, 0.060 mmol, 1.0 eq) was dissolved in round bottom flask **B** in CH_2Cl_2 (200 ml) and was left stirred at 0°C. The solution in **A** was additionally diluted with CH_2Cl_2 (3 ml) and was added dropwise in **B** over 30 min. The reaction mixture was stirred at 0°C for 24 h and was monitored by analytical RP-HPLC. The solvent was evaporated under reduced pressure to provide analogue **17** as a blue solid (69.3 mg, 0.082 mmol). The crude reaction mixture was purified by preparative HPLC (gradient separation 60 to 100% ACN in 45 min, flow rate 12 ml/min) to provide analogue **17** as blue solid (8.5 mg, 0.010 mmol, 16.83%).

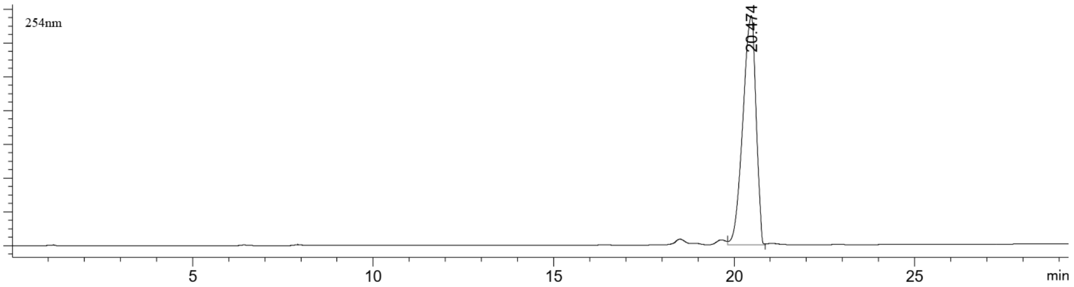

Figure S59: Analytical RP-HPLC chromatogram of synthesized analogue **17** at 254 nm.
RP-HPLC Conditions:
i) Column: Agilent ZORBAX Eclipse Plus C18 (3.5 µm, 100 x 4.6 mm), flow rate 1 ml/min,
ii) Solvents: H_2O (0.08 % TFA), ACN (0.08 % TFA),
iii) Gradient elution: from 40% ACN to 100 % ACN in 30 min at RT,
iv) t_R: 20.474 min; Purity: 98%.

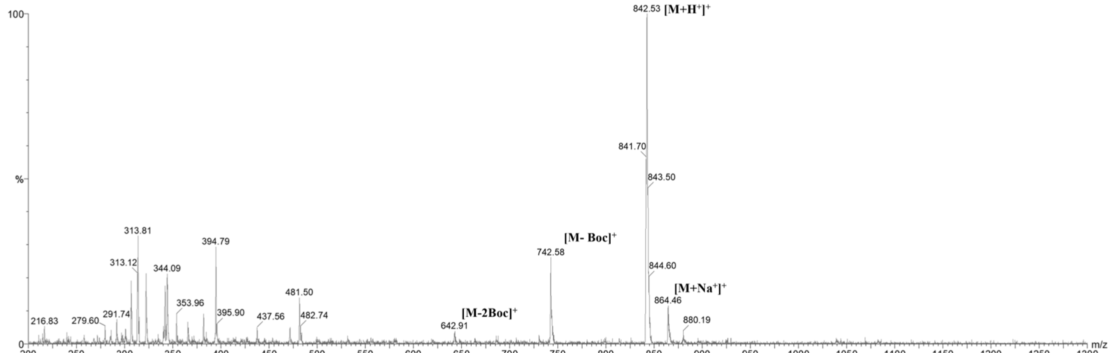

Figure S60: ESI-MS of synthesized analogue **17**. $M_{theoritical}$: 841.99; $(M_{theoritical} + H)^+$: 842.99; found 842.53; $(M_{theoritical} + Na^+)^+$: 864.99; found 864.46; $(M_{theoritical} - Boc)^+$: 740.86; found 742.58; $(M_{theoritical} - 2Boc)^+$: 639.73; found 642.91.

S.2.3.2.2 Synthesis of mitoxantrone analogue 18

Eventually, the removal of Boc-protecting groups of analogue **17** (8.5 mg) was achieved by treatment with a mixture of TFA/ CH_2Cl_2 1:1 (1 ml) and 3 drops of TES at RT for 1.5 h. Solvents were removed under reduced pressure and purification by preparative HPLC (gradient separation 20 to 70% ACN in 43 min, flow rate 12 ml/min) provided analogue **18** as blue solid (5.3 mg, 0.011 mmol, 82.59%).

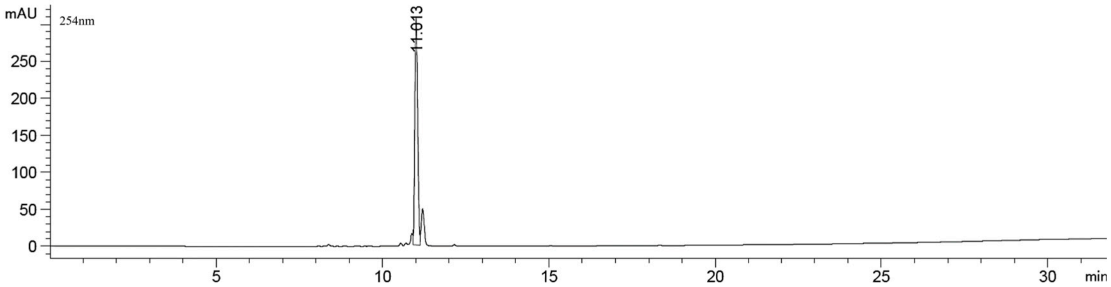

Figure S61: Analytical RP-HPLC chromatogram of synthesized analogue **18** at 254 nm.
RP-HPLC Conditions:
i) Column: Agilent ZORBAX Eclipse Plus C18 (3.5 µm, 100 x 4.6 mm), flow rate 1 ml/min,
ii) Solvents: H_2O (0.08 % TFA), ACN (0.08 % TFA),
iii) Gradient elution: from 10% ACN to 100 % ACN in 30 min at RT,
iv) t_R: 11.013 min; Purity: 85.7%.

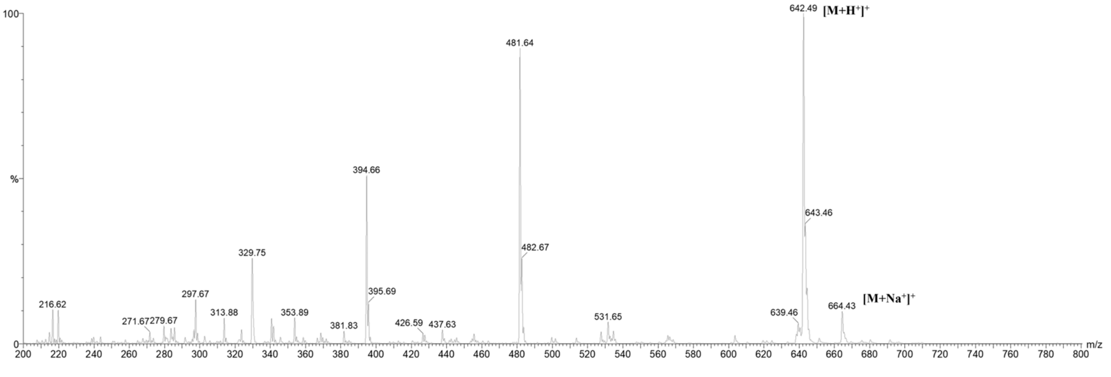

Figure S62: ESI-MS of synthesized analogue **18**. $M_{theoritical}$: 641.76; $(M_{theoritical} + H)^+$: 642.76; found 642.49; $(M_{theoritical} + Na^+)^+$: 665.76; found 664.43.

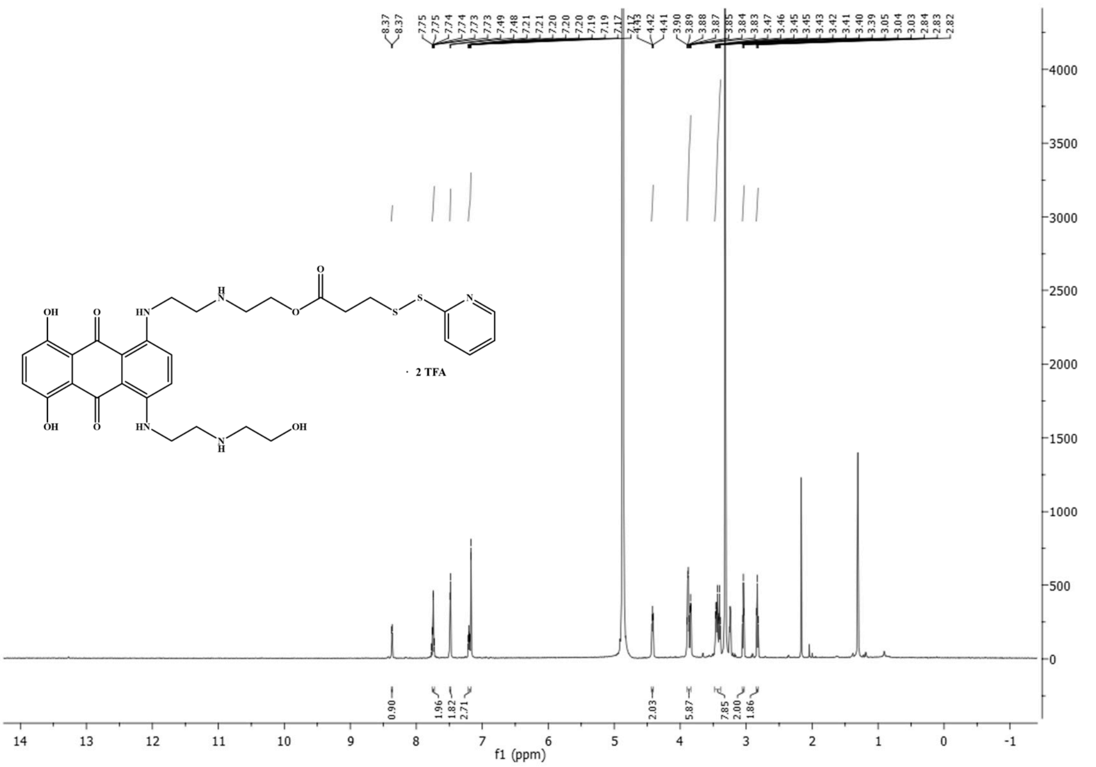

Figure S63: ^{1}H-NMR of synthesized analogue **18**. ^{1}H-NMR (d^4-MeOD, 600 MHz) δ 8.37 – 8.36 (m, 1H, CH^{Ar}), 7.77 – 7.73 (m, 2H, CH^{Ar}), 7.49 – 7.48 (m, 2H, CH^{Ar}), 7.21 – 7.17 (m, 3H, CH^{Ar}), 4.43 – 4.40 (m, 2H, CH_2O), 3.89 – 3.83 (m, 6H, CH_2OH, CH_2N), 3.47 – 3.39 (m, 8H, CH_2N), 3.04 (t, J = 6.9 Hz, 2H, CH_2), 2.83 (t, J = 6.9 Hz, 2H, CH_2).

S.2.3.2.3 Data of mitoxantrone- GnRH conjugate con3

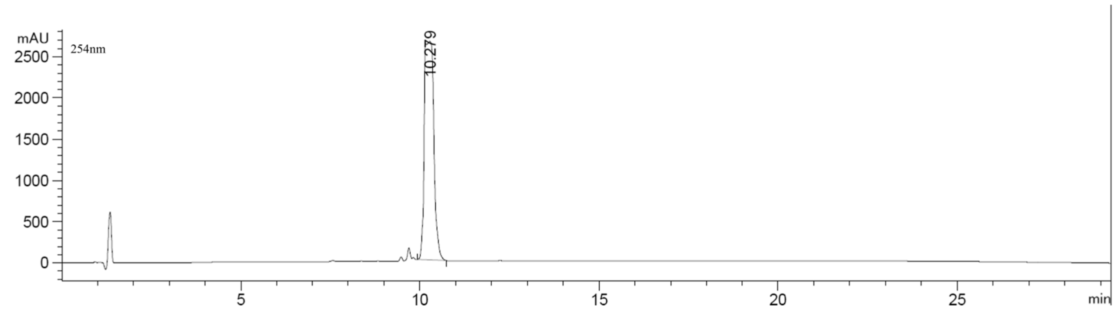

Figure S64: Analytical RP-HPLC chromatogram of synthesized conjugate **con3** at 214 nm.

RP-HPLC Conditions:

i) Column: Agilent ZORBAX Eclipse Plus C18 (3.5 µm, 100 x 4.6 mm), flow rate 1 ml/min,

ii) Solvents: H_2O (0.08 % TFA), ACN (0.08 % TFA),

iii) Gradient elution: from 10% ACN to 100 % ACN in 30 min at RT,

iv) t_R: 10.279 min; Purity: 98.2%.

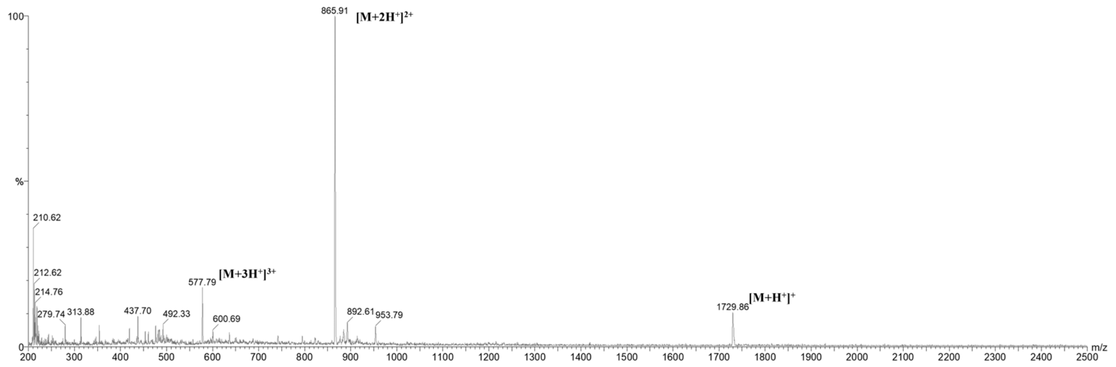

Figure S65: ESI-MS of synthesized conjugate **con3**. $M_{theoritical}$: 1730.00; $(M_{theoritical} + H)^+$: 1731.00; found 1729.86; $(M_{theoritical} + 2H^+)/2$: 866.00; found 865.91; $(M_{theoritical} + 3H^+)/3$: 577.67; found 577.79.

S.3 Conformational studies of con7 and con3

All structures were optimized with the GAMESS software [1,2], employing the density functional theory (DFT) [3,4] and the Hartree - Fock (HF) approximation. Additionally, the B3LYP/6-311G atomic basis set was used and the convergence criterion was set at 0.0001 [4,5]. The force fields which were used are ff14SB for proteins and GAFF (General Amber Force Fields) for organic molecules. All analogues were dissolved in water according to the TIP3P water model in an octahedral cube with a 10Å edge. The overall charge of the analogues was neutralized by the addition of Cl^- ions.

Initially, the energy minimization of the systems was carried out in two steps. In the first step, a force constant for restraint was used in the peptide backbone (10 kcal mol^{-1} $Å^{-2}$), while in the second step, the force constraint was removed. The minimization for the first 5000 steps of each stage was performed using the "steepest descent" method, while in the subsequent steps of each stage, the minimization was performed using the "conjugate gradient" method.

The first phase of the MD was the gradual heating from 0 K to 300 K using the Langevin thermostat with a time step of 0.002 ps for a total time of 0.5 ns. The second phase involved the pressure equilibration of the system at 1 atm with a time step of 0.002 ps for a total time of 0.5 ns. A force constant of (10 kcal mol^{-1} $Å^{-2}$) was applied to the peptide backbone. The third phase of the MD is the equilibration of the system at the desired temperature and pressure (NPT ensemble) without a force constant restraint and a total time of 1 ns. The last step of MD is the production phase, where the system is simulated at constant temperature and pressure for 150 ns. Throughout the simulation, the temperature was kept stable using the Langevin thermostat and the SHAKE algorithm was employed to constrain the motion of bonds involving hydrogen.

All conformations were analyzed for the determination of RMSD, Atomic fluctuation, Clustering and Hydrogen Bond interactions using the cpptraj module of the AMBER14. The clustering analysis was based on the hierarchical approach with an RMSD cutoff of 4.0Å. The RMSD values for all analogues were calculated for the peptide backbone atoms in reference to the initial conformation of every analogue.

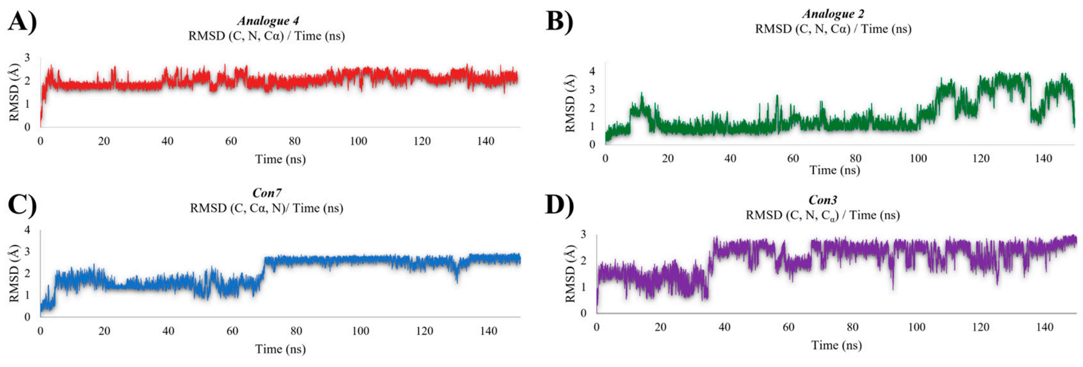

Figure S66: RMSD values for the backbone atoms (C, N, C_α) of A) analogue **4**, B) analogue **2**, C) **con7** and D) **con3**, compared to their initial conformation over the MD simulation time (150 ns).

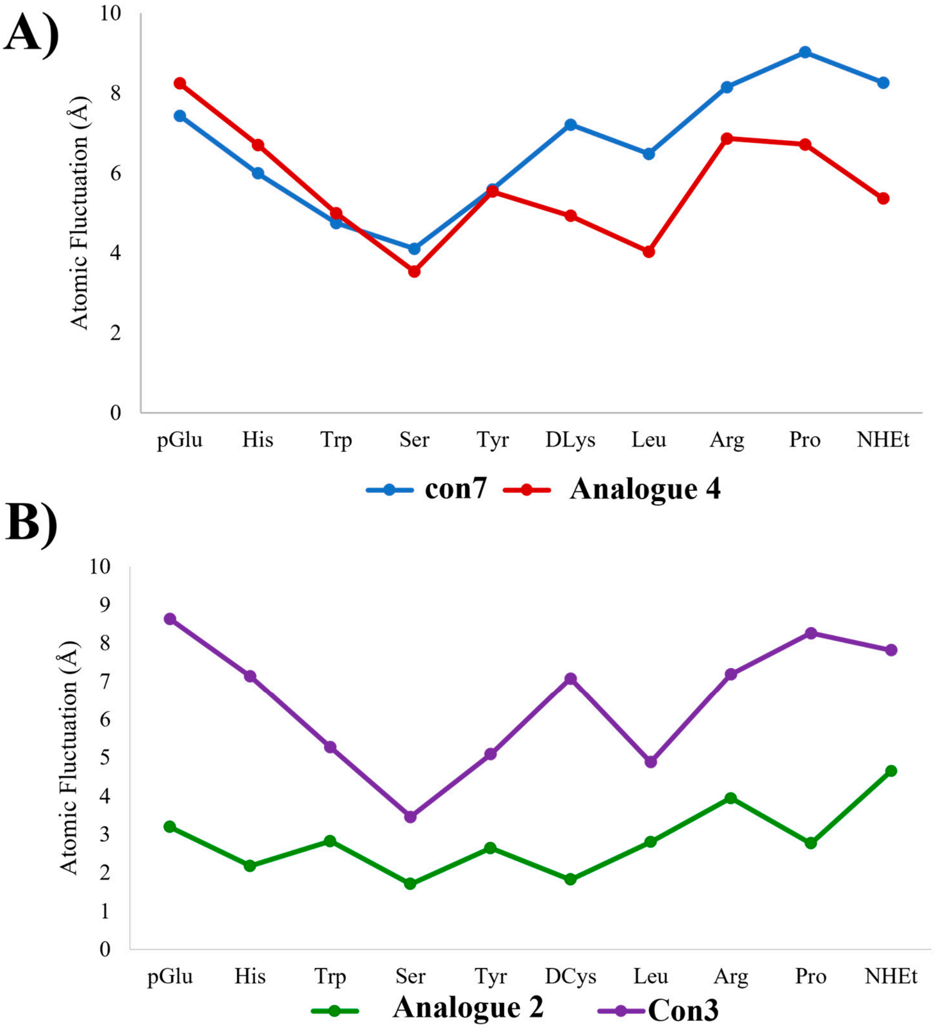

Figure S67: Atomic fluctuations (in Å) for the backbone atoms (C, N, C_α) of A) **con7** and analogue **4**, and B) **con3** and analogue **2.**

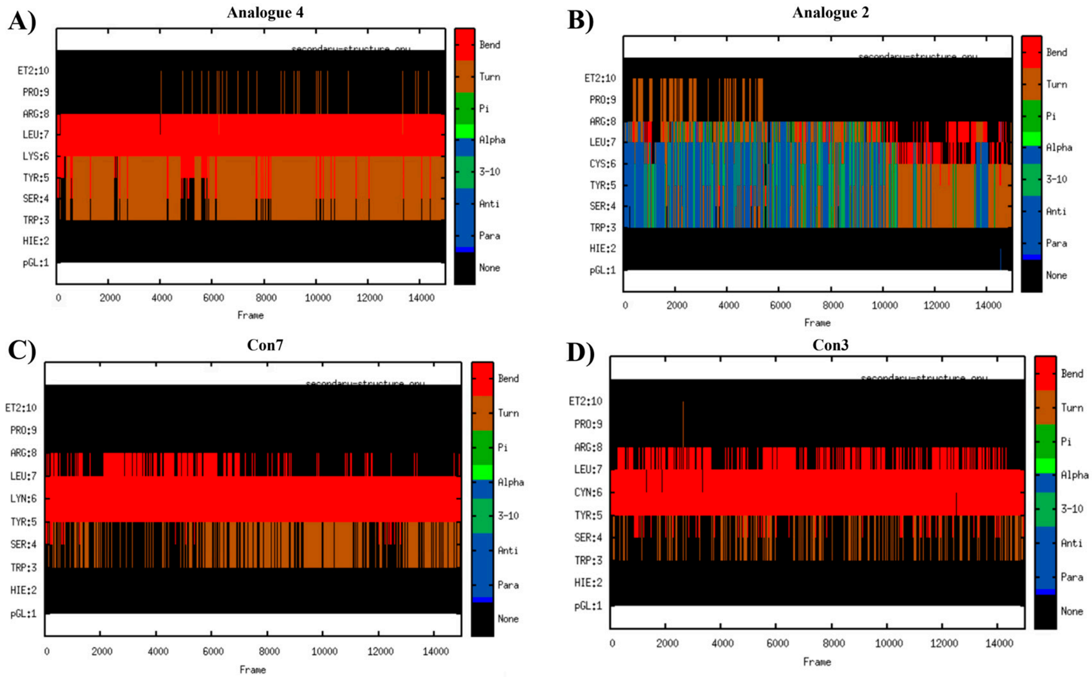

Figure S68: Secondary structure features revealed for A) analogue **4**, B) analogue **2**, C) **con7** and D) **con3** over the MS simulation time.

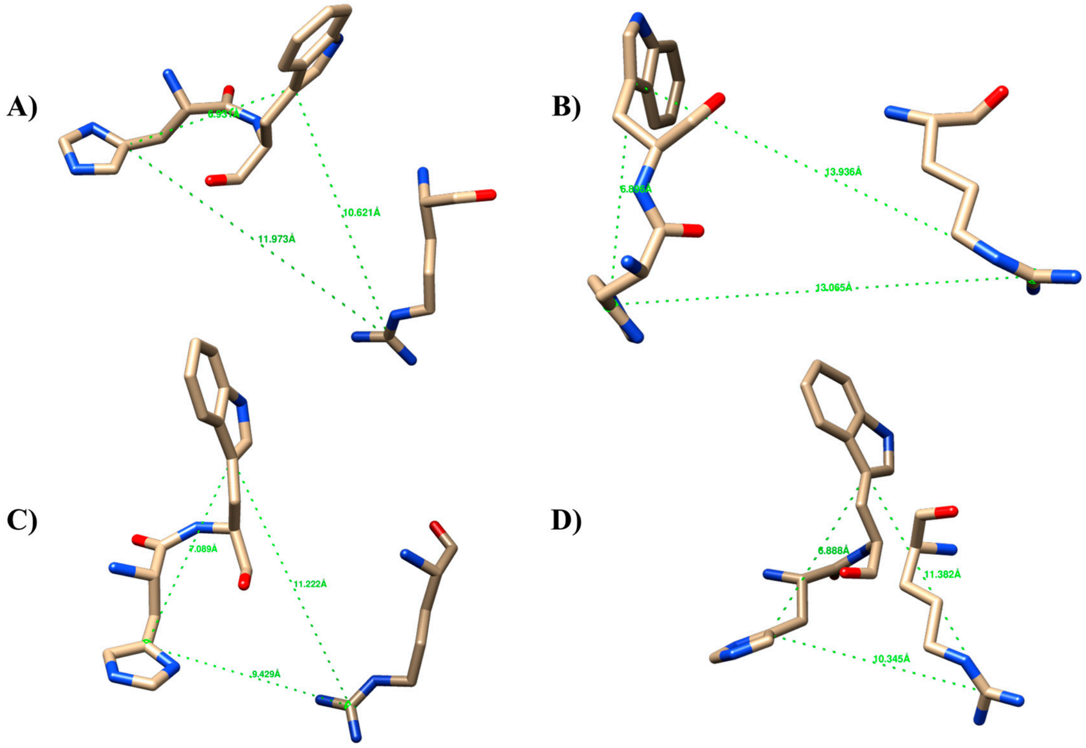

Figure S69: Triangles formed by C_γ of His[2] and Trp[3] and C_ζ of Arg[8] for the putative bioactive conformation of A) analogue **4**, B) analogue **2**, C) **con7** and D) **con3**.

Table S1: Geometric triangles formed by C_γ of His[2] and Trp[3] and C_ζ of Arg[8] for the putative bioactive conformation of analogue **4**, analogue **2**, **con7** and **con3**, compared to **Leuprolide** [6–8].

**Distances**	Analogue **4**	Analogue **2**	**con7**	**con3**	**Leuprolide** [6–8]
d (His^2 – Trp^3)	6.93 Å	6.90 Å	7.09 Å	6.89 Å	6.21 Å
d (His^2 – Arg^8)	11.97 Å	13.07 Å	9.43 Å	10.35 Å	9.43 Å
d (Trp^3 – Arg^8)	10.62 Å	13.94 Å	11.22 Å	11.38 Å	12.03 Å

S.4 Disulfides reduction of con7 and insulin by EcoTrx1

Table S2: Absorbance values from disulfide bond reduction assay by EcoTrx1 for **insulin** for 5 min (300 sec). $A_{sample1}$ and $A_{sample2}$ contain **insuline** and TrxR. $A_{sample3}$ and $A_{sample4}$ contain **insulin** without TrxR.

**Time (sec)**	$A_{sample1}$	$A_{sample2}$	**Average**$_{1,2}$	$A_{sample33}$	$A_{sample4}$	**Average**$_{3,4}$
0	0.236	0.257	0.247	0.237	0.256	0.247
9	0.236	0.257	0.247	0.238	0.256	0.247
18	0.236	0.257	0.247	0.239	0.256	0.248
27	0.235	0.257	0.246	0.238	0.256	0.247
36	0.236	0.256	0.246	0.239	0.256	0.248
45	0.235	0.256	0.246	0.238	0.256	0.247
54	0.235	0.255	0.245	0.238	0.256	0.247
63	0.235	0.256	0.246	0.239	0.257	0.248
72	0.235	0.255	0.245	0.238	0.255	0.247
81	0.233	0.254	0.244	0.237	0.255	0.246
90	0.235	0.255	0.245	0.237	0.256	0.247
99	0.234	0.254	0.244	0.238	0.256	0.247
108	0.234	0.255	0.245	0.239	0.256	0.248
117	0.235	0.253	0.244	0.238	0.255	0.247
126	0.234	0.253	0.244	0.238	0.256	0.247
135	0.235	0.253	0.244	0.239	0.256	0.248
144	0.234	0.253	0.244	0.237	0.255	0.246
153	0.232	0.252	0.242	0.237	0.255	0.246
162	0.233	0.253	0.243	0.240	0.256	0.248
171	0.233	0.252	0.243	0.238	0.255	0.247
180	0.233	0.252	0.243	0.238	0.256	0.247
189	0.232	0.252	0.242	0.239	0.255	0.247
198	0.233	0.252	0.243	0.237	0.256	0.247
207	0.232	0.252	0.242	0.238	0.256	0.247
216	0.233	0.252	0.243	0.239	0.256	0.248
225	0.232	0.252	0.242	0.238	0.256	0.247
234	0.232	0.252	0.242	0.238	0.256	0.247
243	0.231	0.251	0.241	0.238	0.255	0.247
252	0.231	0.251	0.241	0.238	0.255	0.247
261	0.230	0.250	0.240	0.237	0.254	0.246
270	0.230	0.250	0.240	0.238	0.255	0.247
279	0.231	0.251	0.241	0.238	0.256	0.247
288	0.230	0.250	0.240	0.238	0.255	0.247
297	0.229	0.250	0.240	0.238	0.255	0.247

Table S3: Absorabnces values from disulfide bond reduction assay by EcoTrx1 for **con7** for 5 min (300 sec). $A_{sample1}$ and $A_{sample2}$ contain **con7** and TrxR. $A_{sample3}$ and $A_{sample4}$ contain **con7** without TrxR.

Time (sec)	$A_{sample1}$	$A_{sample2}$	$Average_{1,2}$	$A_{sample3}$	$A_{sample4}$	$Average_{3,4}$
0	1.004	0.982	0.993	0.979	1.007	0.986
9	1.004	0.980	0.992	0.978	1.007	0.985
18	1.004	0.981	0.992	0.979	1.007	0.986
27	1.003	0.980	0.991	0.978	1.007	0.985
36	1.002	0.978	0.990	0.978	1.007	0.984
45	1.000	0.979	0.989	0.978	1.006	0.984
54	1.000	0.977	0.988	0.977	1.007	0.983
63	0.999	0.977	0.988	0.978	1.006	0.983
72	0.999	0.976	0.987	0.978	1.007	0.983
81	0.998	0.973	0.985	0.977	1.006	0.981
90	0.997	0.974	0.985	0.978	1.006	0.982
99	0.996	0.972	0.984	0.977	1.007	0.980
108	0.996	0.971	0.983	0.977	1.007	0.980
117	0.995	0.971	0.983	0.977	1.006	0.980
126	0.994	0.970	0.982	0.976	1.006	0.979
135	0.992	0.968	0.980	0.976	1.006	0.978
144	0.991	0.967	0.979	0.976	1.007	0.978
153	0.990	0.966	0.978	0.975	1.007	0.977
162	0.990	0.966	0.978	0.977	1.008	0.978
171	0.989	0.964	0.977	0.976	1.006	0.976
180	0.989	0.965	0.977	0.976	1.006	0.977
189	0.989	0.964	0.977	0.976	1.007	0.976
198	0.988	0.962	0.975	0.974	1.006	0.975
207	0.986	0.961	0.974	0.973	1.005	0.973
216	0.987	0.960	0.974	0.975	1.006	0.974
225	0.985	0.959	0.972	0.973	1.005	0.973
234	0.984	0.957	0.971	0.972	1.005	0.971
243	0.984	0.955	0.970	0.972	1.004	0.971
252	0.982	0.954	0.968	0.971	1.003	0.969
261	0.982	0.952	0.967	0.971	1.003	0.969
270	0.980	0.952	0.966	0.972	1.002	0.969
279	0.981	0.951	0.966	0.971	1.002	0.968
288	0.979	0.949	0.964	0.972	1.002	0.968
297	0.978	0.949	0.963	0.971	1.002	0.967

S.5 Binding affinity studies of mitoxantrone, [DLys^6, Pro^9-NHEt]GnRH (analogue 4) and [DCys^6, Pro^9-NHEt]GnRH (analogue 2)

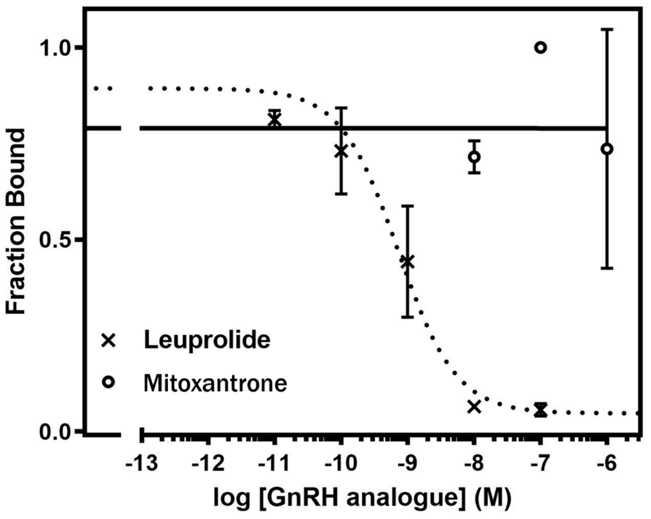

Figure S70: Competition binding of leuprolide and mitoxantrone to human GnRHR. Competition of ^{125}I-Tyr^6, His^5-GnRH specific binding by increasing concentrations of leuprolide and mitoxantrone were performed, as described in section 3.4, on membranes from HEK 293 cells stably expressing the human GnRHR I. The means and S.E. are shown from a representative experiment performed 1-3 times with similar results. The data were fitted to a one-site competition model by nonlinear regression and the IC50 values were determined as described in section 3.4. The mean IC50 values leuprolide and mitoxantrone were 0,64 and >1000 nM, respectively.

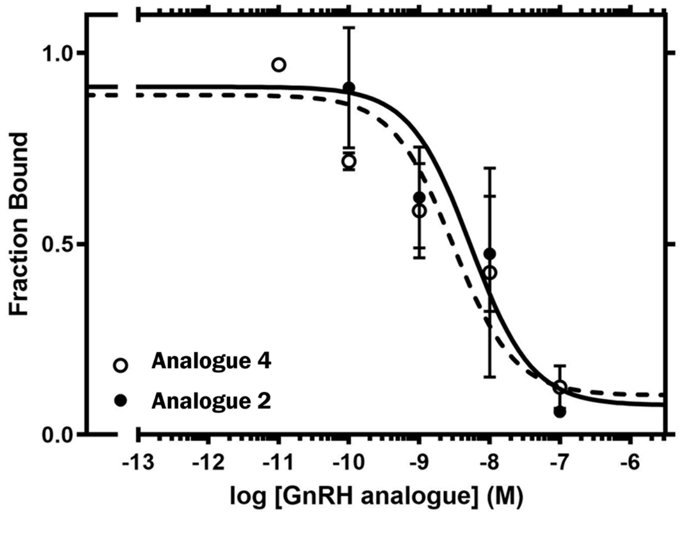

Figure S71: Competition binding isotherms of GnRH analogues to human GnRHR. Competition of ^{125}I-Tyr^6, His^5-GnRH specific binding by increasing concentrations of GnRH analogues, analogue **4** (the peptide that is included in **con7**) and analogue **2** (the peptide that is included in **con3**) was performed, as described in section 3.4, on membranes from HEK 293 cells stably expressing the human GnRHR I. The means and S.E. are shown from a representative experiment performed 2-3 times with similar results. The data were fitted to a one-site competition model by nonlinear

regression and the IC_{50} values were determined as described in section 3.4. The mean IC_{50} values for analogue **4** and analogue **2** were 2.5 and 0.7 nM, respectively.

S.6 References

1. Schmidt, M.W.; Baldridge, K.K.; Boatz, J.A.; Elbert, S.T.; Gordon, M.S.; Jensen, J.H.; Koseki, S.; Matsunaga, N.; Nguyen, K.A.; Su, S.; et al. General Atomic and Molecular Electronic Structure System. *J Comput Chem* **1993**, *14*, 1347–1363, doi:https://doi.org/10.1002/jcc.540141112. (Same as manuscript's reference 84).

2. Gordon, M.S.; Schmidt, M.W. Chapter 41 - Advances in Electronic Structure Theory: GAMESS a Decade Later. In *Theory and Applications of Computational Chemistry*; Dykstra, C.E., Frenking, G., Kim, K.S., Scuseria, G.E., Eds.; Elsevier: Amsterdam, 2005; pp. 1167–1189 ISBN 978-0-444-51719-7. (Same as manuscript's reference 85).

3. Becke, A.D. Density-functional Thermochemistry. III. The Role of Exact Exchange. *J Chem Phys* **1993**, *98*, 5648–5652, doi:10.1063/1.464913. (Same as manuscript's reference 86).

4. Pople, J.A.; Gill, P.M.W.; Johnson, B.G. Kohn—Sham Density-Functional Theory within a Finite Basis Set. *Chem Phys Lett* **1992**, *199*, 557–560, doi:https://doi.org/10.1016/0009-2614(92)85009-Y. (Same as manuscript's reference 87)

5. Hertwig, R.H.; Koch, W. On the Parameterization of the Local Correlation Functional. What Is Becke-3-LYP? *Chem Phys Lett* **1997**, *268*, 345–351, doi:https://doi.org/10.1016/S0009-2614(97)00207-8. (Same as manuscript's reference 88).

6. Meyer, J.D.; Manning, M.C.; Vander Velde, D.G. Characterization of the Solution Conformations of Leuprolide. *J. Pept. Res.* **2002**, *60*, 159–168. (Same as manuscript's reference 31).

7. Fujino, M.; Fukuda, T.; Shinagawa, S.; Kobayashi, S.; Yamazaki, I.; Nakayama, R.; Seely, J.H.; White, W.F.; Rippel, R.H. Synthetic Analogs of Luteinizing Hormone Releasing Hormone (LH-RH) Substituted in Position 6 and 10. *Biochem Biophys Res Commun* **1974**, *60*, 406–413, doi:https://doi.org/10.1016/0006-291X(74)90219-8. (Same as manuscript's reference 33).

8. Laimou, D.K.; Katsara, M.; Matsoukas, M.T.I.; Apostolopoulos, V.; Troganis, A.N.; Tselios, T. V. Structural Elucidation of Leuprolide and Its Analogues in Solution: Insight into Their Bioactive Conformation. *Amino Acids* **2010**, *39*, 1147–1160, doi:10.1007/s00726-010-0549-8. (Same as manuscript's reference 80).
